# Telomere-to-telomere chromosome-scale genome assemblies of black and golden koi carp variants support construction of an ancient karyotype of Cypriniformes

**DOI:** 10.1093/gigascience/giaf073

**Published:** 2025-07-26

**Authors:** Chao Bian, Rujingwen Huan, Qiong Shi

**Affiliations:** Laboratory of Aquatic Genomics, College of Life Sciences and Oceanography, Shenzhen University, Shenzhen, Guangdong 518057, China; Shenzhen Key Lab of Marine Genomics, BGI Academy of Marine Sciences, BGI Marine, Shenzhen, Guangdong 518081, China; Laboratory of Aquatic Genomics, College of Life Sciences and Oceanography, Shenzhen University, Shenzhen, Guangdong 518057, China; Laboratory of Aquatic Genomics, College of Life Sciences and Oceanography, Shenzhen University, Shenzhen, Guangdong 518057, China; Shenzhen Key Lab of Marine Genomics, BGI Academy of Marine Sciences, BGI Marine, Shenzhen, Guangdong 518081, China

**Keywords:** genetics and genomics, koi carp, ancestral chromosome

## Abstract

**Background:**

Koi carp, a variant of the common carp, is one of the most popular ornamental fish. Its genomic resources can help us better understand chromosome evolution and color phenotypes in cyprinid fish.

**Results:**

We constructed telomere-to-telomere chromosome-level genome assemblies for 2 koi carp variants (black and golden) by integrating MGI, PacBio HiFi, ONT, and Hi-C sequencing technologies. Haplotypic genomes comprised 50 chromosomes with 100 and 99 telomeres, respectively, with BUSCO results showing at least 98.8% completeness. We annotated a total of 55,023 and 54,569 protein-coding genes for black and golden koi carps, respectively, with over 96% assigned functional roles. Repetitive sequences occupy an estimated 636 Mb (41%) of the genomes. With phylogenetic analysis, we predict the koi carp variants to have split 5.3 million years ago, and we constructed an ancient karyotype of 25 ancestral chromosomes to reveal 9 major chromosomal rearrangements.

**Conclusions:**

Our study offers genome assemblies capable of predicting an ancient karyotype of Cypriniformes, with genomic resources available for in-depth investigations into diverse skin coloration in koi and other cypriniforms.

## Introduction

Cypriniforms exhibit distinctive evolutionary characteristics in ecological adaptability and phenotypic diversity, serving as excellent models for polyploid genome evolution. Cyprinid species such as the common carp (*Cyprinus carpio*) and goldfish (*Carassius auratus*) display genomic complexity, originating from a special allotetraploidization event [1, 2], which led to asymmetric evolution between the 2 subgenomes (A and B) [3–5]. This asymmetry manifests as significant differences in gene retention rates, expression patterns, and methylation levels [3–7]. Studies suggest that, during the rediploidization process, allotetraploid cyprinids employ mechanisms—including chromosomal rearrangements, transposon dynamics equilibrium, and coordinated *cis*-/*trans*-regulatory interactions—to maintain subgenome stability and balanced expression [7–9].

Beyond their special genomic structure, coloration in cyprinids holds critical ecological and economic significance. Koi carp (*Cyprinus carpio var. koi*, NCBI Taxonomy ID: 1,499,333), a member of the Cyprinidae family, is one of the most popular ornamental fish species due to its diverse coloration. Recent research on koi carp has revealed the role of microRNA in carotenoid metabolism and pigmentation regulation [10]. Transcriptomic analyses have further identified key genes (such as *pax3, pax7*, and *gch2*) as crucial regulators of pigmentation and neural crest development during early embryogenesis [11].

In this study, we report the first 2 telomere-to-telomere (T2T) genome assemblies for black and golden koi carp variants. Through ancestral chromosome reconstruction for cypriniforms, we reveal that their karyotypes underwent 9 major chromosomal rearrangements over the course of evolution. The genome assemblies of black and golden koi carp variants not only advance our understanding of cyprinid genomic evolution but also are essential resources for deciphering the genetic basis of phenotypic diversification within this family.

## Methods

### Sample collection, DNA extraction, and whole-genome sequencing

Two female koi carp variants (1 black and 1 golden) were collected from a local aquaculture farm in Guangzhou, Guangdong Province, China. We pooled muscle samples separately from both koi carps and extracted genomic DNA (gDNA) from both samples for whole-genome sequencing using MGI to produce short reads, PacBio HiFi and ONT to produce long reads, and high-throughput chromatin conformation capture (Hi-C) technologies [12].

For gDNA sequencing, libraries with an insert size of 500 bp were constructed using a MGIEasy UDB Universal Library Prep Set, following the manufacturer’s instructions (MGI Tech Co. Ltd.; RRID:SCR_017981). These libraries were subsequently sequenced on a DNBSEQ-T7 machine (MGI). The HiFi long-read libraries were prepared using a SMRTbell Express Template Prep Kit 2.0 (Pacific Biosciences), following manufacturer protocols. HiFi long-read data were generated through sequencing on a PacBio Sequel II platform (Pacific Biosciences; RRID:SCR_017990) [13]. Next, CCS software (SMRT Link v9.0) was employed to generate consensus sequences [14]. Both ONT ultra-long libraries were built using an Oxford Nanopore SQK-ULK001 kit according to manufacturer’s instructions (Oxford Nanopore Technologies), which were then sequenced on a PromethION flow cell (Oxford Nanopore Technologies; RRID:SCR_017987). The ONT reads were corrected by NECAT v200221 software (RRID: SCR_025,350) with default parameters [15]. Both Hi-C libraries were constructed using a GrandOmics Hi-C Kit and *DpnII* enzyme according to the manufacturer’s standard protocol (GrandOmics). Sequencing was conducted on an Illumina NovaSeq platform (Illumina; RRID: SCR_016,387). All sequencing reads were integrated for genome assembling and chromosome anchoring.

Total RNA samples were prepared from muscle, skin, liver, and heart tissues of the koi carps using a TRIZOL Kit (Invitrogen) in accordance with the manufacturer’s instructions. The integrity and quality of extracted RNA were evaluated using an Agilent 2100 Bioanalyzer (Agilent Technologies; RRID: SCR_018,043), and only those samples with an RNA integrity number (RIN) over 7.0 were selected for subsequent library preparation. Next, cDNA libraries were constructed using DNA nanoballs in accordance with the manufacturer’s protocol for a DNBSEQ platform (MGI; RRID:SCR_017981). These libraries were sequenced on a MGISEQ-2000 platform (MGI) with a paired-end model (150 bp long).

### Genome size prediction, genome assembling, telomere identification, and assembly quality evaluation

Genome sizes of the black and golden koi carps were estimated using a 17-mer frequency distribution analysis [16] of cleaned MGI data (with an insert size of 500 bp) and calculated according to the following equation: genome size = *k*-mer number / the expectation of *k*-mer depth.

Initial genome assemblies were completed using Hifiasm v0.19.8 (detailed parameters: -t 16 –n-hap 4 –hg-size 1520 m; RRID: SCR_021,069) with PacBio HiFi and ONT reads [17]. The Hi-C sequencing reads were aligned onto the above assembled contigs using Bowtie 2 (parameters: –very-sensitive -L 30–score-min L, −0.6, −0.2–end-to-end –reorder; RRID:SCR_016368) [18], and YaHs v1.0 (RRID: SCR_022,965) [19] was employed with default parameters to compute the chromosomal linkage information, based on alignment results. These alignments were subsequently used with Juicer v1.5 (parameters: chr_num 30; RRID:SCR_017226) [20] and 3D-DNA v170123 (parameters: -m haploid -r 2; RRID:SCR_017227) [21] to anchor contigs onto primary chromosomes. Juicebox v1.11.08 (RRID:SCR_021172) [22] was used to refine the assembly. The primary chromosome-level genome assemblies of black and golden koi carps contained 7 and 8 gaps, respectively. To achieve a gap-free and T2T level, we sequentially applied LR_GapCloser v1.0 (parameters: -t 35 -m 1,000,000 -v 10,000; RRID:SCR_016194) [23] and TGS-GapCloser v1.0.1 (parameter: -min_match 2000; RRID:SCR_017633) [24] to fill gaps within both genome assemblies. Centromere and telomere sequences were identified using QuarTeT software (RRID:SCR_025258) [25].

For quality evaluation of both assemblies, a BUSCO (RRID:SCR015008) [26] evaluation was performed to predict completeness. We mapped PacBio HiFi and ONT reads onto the genome assemblies using Minimap2 (RRID:SCR018550) [27] to conduct more assembling correction. Quality value (QV) was estimated using Merqury-20,200,430 software (RRID: SCR_022,964) [28], with the recommended *k*-mer of 20.

### Repeat element annotation

Repeat elements in both genomes were identified through a combination of *de novo* and homology-based methods. For *de novo* prediction, RepeatModeler v1.0.8 (RRID:SCR_015027) [29] and LTR_Finder v1.0.6 (RRID:SCR_015247) [30] were applied to detect different types of repeat elements. Then we generated both new repeat libraries by integrating RepeatMasker v4.0.623 (RRID:SCR_012954) [31] and Repbase TE v21.01 (RRID:SCR_021169) [32]. Tandem repeats were detected using Tandem Repeats Finder (parameters: 2 7 7 80 10 50 2000 -d -h; RRID:SCR_022193) [33]. With the new repeat libraries, we employed RepeatProteinMask v4.0.623 [31] and RepeatMasker v4.0.623 (RRID:SCR_012954) [31] to identify repetitive sequences.

### Gene prediction and functional annotation

To annotate protein-coding genes, we integrated homology alignment and transcriptome data to generate nonredundant sets of protein-coding genes for both black and golden koi carps. For homology-based annotation, protein sequences of 5 representative species—zebrafish (*Danio rerio*), medaka (*Oryzias latipes*), grass carp (*Ctenopharyngodon idella*), common carp (*Cyprinus carpio var. wuyuanensis*), and golden-line barbel fish (*Sinocyclocheilus anophthalmus*)—were downloaded from the NCBI (RRID:SCR_006472) database and aligned to our assembled genomes using TBLASTN (E-value 10^–5^; RRID:SCR_011822) [34]. Based on TBLASTN alignments, GeneWise v2.2.0 (parameters: –blast_eval 1e-5 –align_rate 0.5 –extend_len 500; RRID:SCR_015054) [35] predicted gene structures. Transcriptome reads were mapped onto the genomic components using HISAT2 (RRID:SCR_015530) [36] and the transcriptome annotation sets generated using Cufflinks v2.2.1 (RRID:SCR_014597) [37].

MAKER (max_dna_len=300,000, min_contig=500, pred_flank=500, AED_threshold=1, split_hit=30,000, single_exon=1, single_length=250, tries=2; RRID:SCR_005309) [38] was then utilized to integrate gene sets generated from the 2 methods, to produce final, nonredundant protein-coding gene sets. Functional annotation was conducted by aligning results with 5 public databases: SwissProt (RRID:SCR_021164) [39], TrEMBL [40], Kyoto Encyclopedia of Genes and Genomes (KEGG; RRID:SCR_012773) [41], Gene Ontology (GO; RRID:SCR_002811) [42], and InterPro (RRID:SCR_005829) [43].

### Gene families, phylogenetic tree construction, and divergence time estimation

Chromosome-level genomes of 5 representative species of Cypriniformes (*D. rerio, C. idella, Gobiocypris rarus, S. anophthalmus, C. carpio var. wuyuanensis*, and *O. latipes*) were downloaded from NCBI for phylogenetic and divergence time analyses. For the phylogenetic analysis, BLASTP (RRID:SCR_001010) [44] and OrthoMCL(RRID:SCR_007839) [45] were performed for protein sequence alignment and gene-family clustering. All single-copy orthologous genes were aligned using MUSCLE v3.8.31(RRID:SCR_011812) [46] for all examined genomes. Then, the Gblocks (RRID:SCR_015945) [47] program was used to obtain conservative multisequence alignments. Finally, we employed PhyML v4.9 (RRID:SCR014932) [48] to construct a phylogenetic tree using the maximum likelihood method. Species divergence times were estimated using MCMCTREE in PAML v4.9 (RRID:SCR014932) [49]. We used a divergence time point from TimeTree data (RRID:SCR021162), at 140–170 million years ago (Mya) between *O. latipes* and *D. rerio* to calibrate divergence times.

### Construction of ancestral chromosomes

From the protein sequences of 7 representative species, Proteinortho v6.0.36 (RRID:SCR_024177) [50] was employed to obtain a single-copy protein set, which was then concatenated into a supergene. A phylogenetic tree was constructed as above. Several previous studies reconstructed the 13 pairs of ancestral chromosomes (a~m) of teleosts, concluding that 8 major rearrangements occurred after the third round of whole-genome duplication (WGD) [51–53]. Thus, the most recent common ancestor of teleosts was putatively considered to harbor 24 ancestral chromosomes [51–53].

Protein datasets of each representative species of Cypriniformes were aligned to the protein set of the predicted ancestral teleosts using BLASTP (with an E-value threshold of 1e-10; RRID:SCR_001010). We filtered out matching regions with same color—fewer than 20 per chromosome. Finally, we predicted chromosome rearrangements, including chromosome fissions, fusions, and translocations. We applied SVG in Perl programming language to visualize the predicted karyotype of the ancestor of Cypriniformes. Gene sequence fragments homologous to the ancestral chromosomes were marked with corresponding colors.

## Results

### Summary of the sequencing reads and both genome assemblies

**Figure 1:**
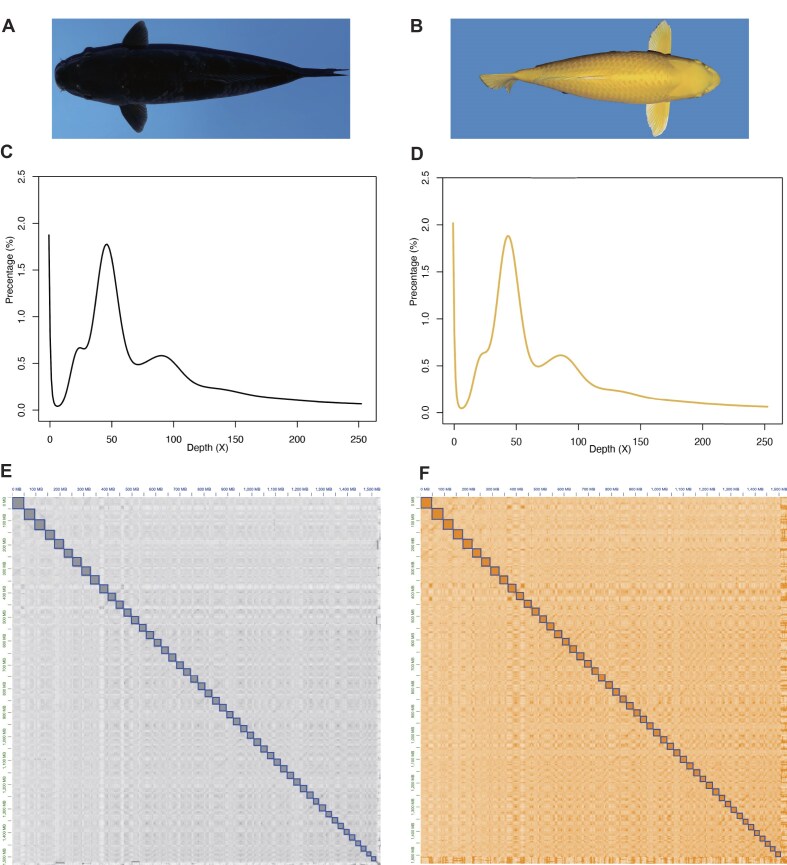
Black and golden koi carps subject to whole-genome sequencing. (A, B) Images of the sequenced black and golden variants. (C, D) The *k*-mer distribution for both genome sequences. (E, F) Chromosome heatmaps of Hi-C data for black and golden koi carps, respectively.

Through the *k*-mer analysis of MGI data (78.6 and 73.7 Gb, respectively; Supplementary Table S1), we estimated the genome sizes of black and golden koi carp variants to be around 1.53 Gb (Fig. 1, Supplementary Fig. S2). We also sequenced both koi genomes using PacBio Sequel II and ONT platforms, obtaining a total of 99.5 and 108.9 Gb of PacBio Sequel long reads and 29.1 and 36.6 Gb of ONT ultra-long reads, respectively (Supplementary Table S3). The initial genomes of assembled black and golden koi carps were 1.57 Gb and 1.55 Gb in length, respectively (Table 1), with their contig N50 reaching 30.0 Mb. We also obtained 204.7 Gb and 215.6 Gb of Hi-C reads for black and golden koi carps, respectively (Supplementary Table S4). Through Hi-C read mapping and contig anchoring, we constructed 50 chromosomes with 7 to 8 gaps in the assembled genomes.

**Table 1:** Statistical analysis of genome assemblies and annotation results for black and golden koi carps

Parameter	Black variant	Golden variant
MGI reads (Gb)	78.6	73.7
HiFi reads (Gb)	99.5	108.9
ONT reads (Gb)	29.1	36.6
Hi-C reads (Gb)	204.7	215.6
Genome size (Mb)	1,568.0	1,553.7
Chromosome N50 (Mb)	30.0	30.0
Gap number	0	0
Telomere number	100	99
Merqury QV	46.8	46.8
BUSCO value	98.9%	98.8%
Repeat ratio	40.6	40.9
Gene number	55,023	54,569
Average gene length (bp)	15,771.5	16,005.4
Functional gene number	52,855	52,975

### After gap closing, both final assemblies reached a high-quality chromosome level

**Figure 2:**
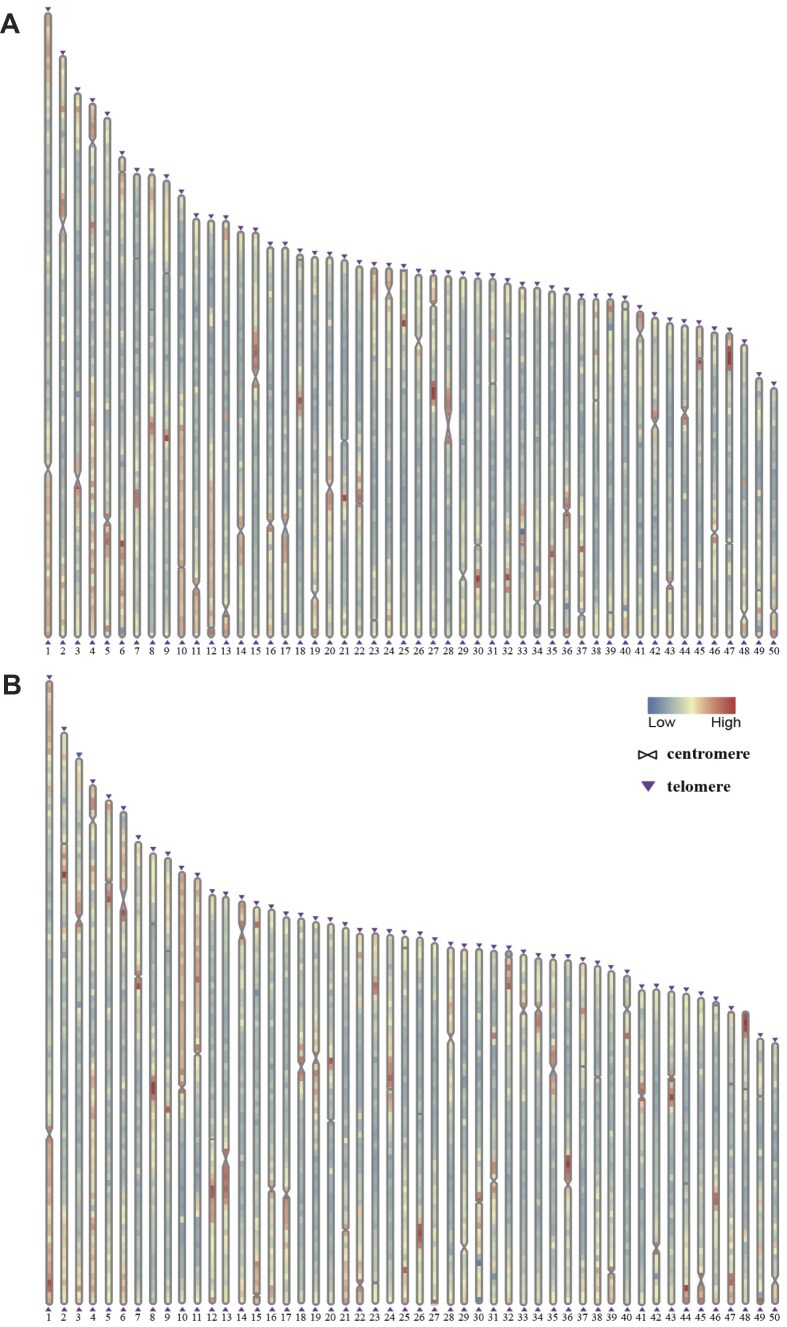
Genome-wide localization of centrosomes and telomeres. Details on each chromosome of black (A) and golden (B) koi carps for comparison.

The total of 50 chromosome sequences for black and golden koi carps comprised up to 1.55 and 1.54 Gb, respectively (see the detailed chromosome lengths corresponding to each of the black and golden koi carps in Supplementary Table S5), accounting for about 98.9% and 99.3% of assembled contigs. The black koi carp genome contained a total of 100 telomeres, and the golden koi carp genome contained 99 (only 1 of its chr48_A10 was not identified; Fig. 2B). Our BUSCO results showed 98.9% and 98.8% completeness (Table 1), respectively. Mapping ONT and HiFi reads onto both assembled genomes, the MGI reads showed high mapped rates (97.0% and 97.6%, respectively). The Merqury program indicated that the quality values of both assemblies were 46.8, equating to a per-base accuracy rate of 0.9999988. The above evaluations confirmed the high quality and completeness of our genome assemblies for black and golden koi carps.

### Repeat elements and gene annotation data

Repeat annotations were performed using both *de novo* and homology-based methods. We predicted that the black and golden koi carp genomes contain 636.21 and 636.24 Mb of repetitive sequences, accounting for 40.6% and 40.9% of their genomes, respectively (Supplementary Table S7).

Gene prediction was carried out using an integrated approach of homology-based and transcriptome-based annotations. We annotated a total of 55,023 and 54,569 protein-coding genes, with an average mRNA length of 15.8 and 16.0 kb, respectively (Supplementary Table S8 and S9). Among them, over 96.1% and 97.1% of predicted genes were assigned with at least 1 functional role from among 5 databases (SwissProt, TrEMBL, KEGG, GO, and InterPro; Supplementary Table S10).

### Predicted divergence times and ancestral chromosomes of Cypriniformes

**Figure 3:**
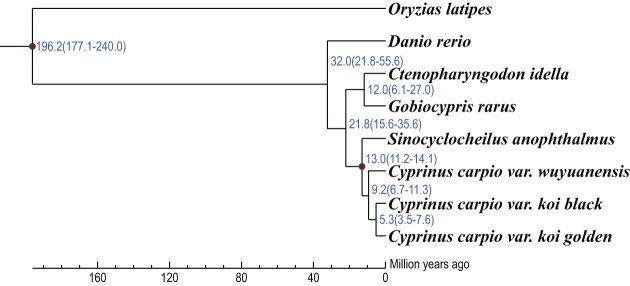
Divergence time tree of 7 representative cypriniforms. Medaka (*Oryzias latipes*) was set as the outgroup. Blue numbers represent the estimated periods of divergence times. The red dot indicates the reference period for time divergence from TimeTree data (http://www.timetree.org/).

**Figure 4:**
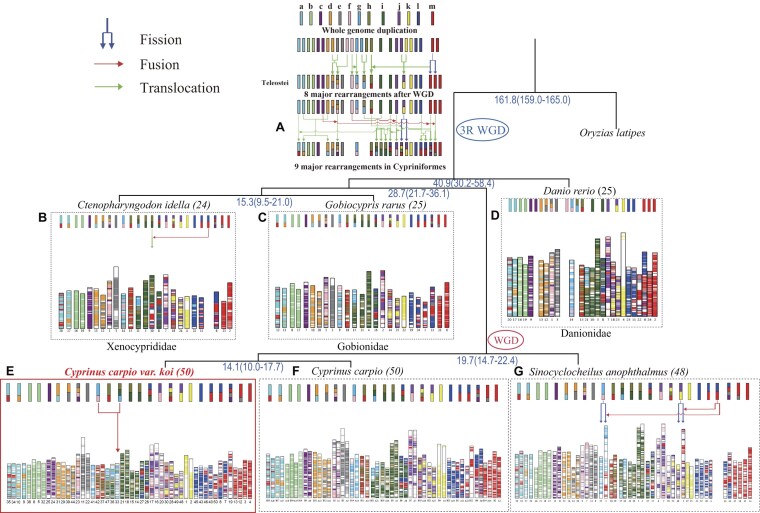
Evolution of chromosome karyotypes in Cypriniformes. Thirteen color bars represent teleost ancestor chromosomes a–m. Blue arrows indicate fission events, red arrows fusion, and green arrows chromosomal translocation events. Red boxes mark new chromosomes after chromosomal fusions. Examined species include ancestors of Cypriniformes (A), *Ctenopharyngodon idella* (B), *Gobiocypris rarus* (C), *Danio rerio* (D), *Cyprinus carpio var. koi* (E), *Cyprinus carpio var. wuyuanensis* (F), and *Sinocyclocheilus maitianheensis* (G).

We predicted the divergence time between *C. carpio var. wuyuanensis* and *C. carpio var. koi* to be approximately 9.2 Mya, and our 2 koi carp variants split from each other about 5.3 Mya (Fig. 3). A total of 25 ancestral chromosomes were predicted with 9 major chromosomal rearrangements in Cypriniformes after divergence from the teleosts’ common ancestor—including 2 fusions, 1 fission, 4 chromosomal translocations, and 2 complex chromosomal rearrangements (see more details in Fig. 4A). We also constructed a phylogenetic tree for 6 representative cypriniform species with different karyotypes (*n* = 24, 25, 48, and 50; Supplementary Table S11) and analyzed detailed variances within these examined species (Fig. 4B–G). For comparison, we summarized total numbers of the best-hit gene pairs between the predicted ancestor and 7 representative cypriniforms, including 2 koi carps (Supplementary Table S12).

These chromosomal rearrangements were conserved in the examined cypriniforms with hyplotypic 25 or 50 chromosomes. Two diploid fishes with a total of 25 chromosomes, including rare minnow (*G. rarus*; Fig. 4C) and zebrafish (*D. rerio*; Fig. 4D), have a similar chromosome structure to the ancestral Cypriniformes chromosomes. Two tetraploid fishes with a total of 50 chromosomes—both purse red carp (*C. carpio wuyuanensis*) and koi carp (*C. carpio var. koi*)—experienced an additional WGD event, but their karyotypes were mostly conserved (Fig. 4E–F). Only a small chromosomal fusion was found in koi carp (Fig. 4E). Some special chromosomal fusions occurred in fishes with 24 and 48 chromosomes; for instance, the diploid grass carp (*C. idella; n* = 24) experienced 1 chromosome fusion (Fig. 4B), while the tetraploid *Sinocyclocheilus* species (*n* = 48) experienced 2 chromosome fusions (Fig. 4G) after the *Sinocyclocheilus-*lineage WGD. Two fusion events and 2 fission events occurred in chromosomes of *Sinocyclocheilus* species (Fig. 4G). We therefore suspect that the ancestors of diploid and tetraploid Cypriniformes were completely separated before independent fusion events.

## Conclusion

The species from Cyprininae contain diploids (2n = 2X = 50 or 48), tetraploids (2n = 4X = 100), hexaploids (2n = 6X ≈ 150) [54], and higher polyploids (2n: 417–470) [55]. The koi carps in the Cyprininae superfamily, Cyprinidae family, Cyprinoidei suborder are tetraploid (2n = 4X = 100). The chromosome-level genome assemblies of Cyprininae fish of different ploidies can largely help to study the chromosome evolution in Cyprininae fish with diverse ploidies. Our study demonstrates the potential for advanced sequencing technologies, particularly third-generation long-read sequencing, to produce high-fidelity genome assemblies, including centromeric regions and numerous telomeres. Compared with previously published common carp genomes, our investigation enhanced accuracy and robustness, yielding more comprehensive telomere-to-telomere representation. The 50 chromosome sequences of black and golden koi carps are up to 1.55 and 1.54 Gb, respectively (see detailed length of subgenome chromosome in Supplementary Table S5), accounting for about 98.9% and 99.3% of the assembled contigs. The contig N50 of both black and golden koi carp genome assemblies achieved 30.0 Mb, representing a dramatic increase over earlier assemblies (0.068 Mb [1] and 1.55 Mb [7]; Supplementary Table S6). Notably, our assemblies are entirely gap-free and achieve complete coverage from one telomere to another, in contrast to earlier versions that still contained unresolved regions. To our knowledge, these represent the first T2T chromosome-level assemblies generated for the common carp. BUSCO analysis confirmed near-complete gene representation (~99%), exceeding that of previous research (Supplementary Table S6).

We successfully resolved nearly all telomeres of the koi genomes. The black koi genome contains a total of 100 telomeres, while the golden koi genome contains 99, with only 1 telomere (on chr48) unresolved. The ability to elucidate telomeric and sub-telomeric regions across koi chromosomes can provide more complete genome assemblies. Through comprehensive genome annotation, we identified 55,023 and 54,569 protein-coding genes in black and golden koi, respectively. We further reconstructed 25 ancestral chromosomes for Cypriniformes and revealed 9 major chromosomal rearrangements after their divergence from a common ancestor of teleosts.

Overall, these high-quality assemblies will facilitate further investigation into the molecular basis of pigmentation and morphological diversity in koi development. They serve as a comprehensive reference for future large-scale resequencing of koi with different body colors and for single-cell transcriptomic profiling of skin tissues. Moreover, we anticipate that these genome resources will inform future efforts in marker-assisted selection and precision breeding of ornamental carp.

## Supplementary Material

giaf073_Supplemental_File

giaf073_Authors_Response_To_Reviewer_Comments_original_submission

giaf073_GIGA-D-24-00549_original_submission

giaf073_GIGA-D-24-00549_Revision_1

giaf073_Reviewer_1_Report_Original_Submission Yoshihiro Omori -- 1/23/2025

giaf073_Reviewer_1_Report_Revision_1 Yoshihiro Omori -- 5/3/2025

giaf073_Reviewer_2_Report_Original_Submission Costas Tsigenopoulos -- 2/6/2025

giaf073_Reviewer_2_Report_Revision_1 Costas Tsigenopoulos -- 5/9/2025

## Data Availability

Genome assemblies of black and golden koi carps in this study have been deposited in the NCBI database (BioProject IDs: PRJNA1191445 and PRJNA1191556). All additional supporting data are available in the *GigaScience* repository, GigaDB [56], with individual datasets for black koi [57] and golden koi [58].

## Additional Files

**Supplementary Table S1**. Statistical analysis of MGI reads for *k*-mer analysis.

**Supplementary Table S2**. Genome size estimation of black and golden koi carps using *k*-mer analysis.

**Supplementary Table S3**. Statistics of sequencing reads from PacBio and ONT sequencing platforms.

**Supplementary Table S4**. Statistical analysis of sequencing reads from Hi-C libraries.

**Supplementary Table S5**. Statistical analysis of the chromosome (Chr) details from available *C. carpio* genome assemblies.

**Supplementary Table S6**. Statistics of genome assemblies for 2 koi carps and 2 published common carps.

**Supplementary Table S7**. Statistical analysis of repeat sequences in the assembled genomes.

**Supplementary Table S8**. Statistics of predicted gene structures in the black koi carp genome.

**Supplementary Table S9**. Statistics of predicted gene structures in the golden koi carp genome.

**Supplementary Table S10**. Summary of functional annotations.

**Supplementary Table S11**. NCBI accession numbers of 6 representative fish genomes for phylogenetic analysis.

**Supplementary Table S12**. Summary of best-hit gene pairs between a predicted ancestor and 7 representative cypriniforms, including 2 koi carps.

## Abbreviations

BUSCO: Benchmarking Universal Single-Copy Orthologs; Gb: gigabyte bases; gDNA: genomic DNA; Hi-C: high-throughput chromatin conformation capture; Mb: million bases; Mya: million years ago; NCBI: National Center for Biotechnology Information; T2T: telomere-to-telomere; WGD: whole-genome duplication.
