# Supplementary material for: Telomere-to-telomere chromosome-scale genome assemblies of black and golden koi carp variants support construction of an ancient karyotype of Cypriniformes
Source: Gigascience. 2025 Jul 26;14:giaf073. doi: 10.1093/gigascience/giaf073 (PMC13223729; doi:10.1093/gigascience/giaf073)
Supplement: giaf073_Supplemental_File [file giaf073_supplemental_file.docx]

**Supplementary Table S1.** Statistical analysis of MGI reads for the *k*-mer analysis.

| **Variant** | **Insert Size (bp)** | **Read Length (bp)** | **Total Data (Gb)** |
| --- | --- | --- | --- |
| black | 500 | 150 | 78.6 |
| golden | 500 | 150 | 73.7 |

**Supplementary Table S2.** Genome size estimation of black and golden koi carps by using a *k*-mer analysis.

| **Variant** | **kmer** | **kmer num** | **Kmer depth** | **genome size (Mb)** | **used base** | **used read** |
| --- | --- | --- | --- | --- | --- | --- |
| black | 17 | 70,198,861,936 | 46 | 1,526.0 | 78,580,815,600 | 523,872,104 |
| golden | 17 | 65,832,088,160 | 43 | 1,531.0 | 73,692,636,000 | 491,284,240 |

**Supplementary Table S3.** Statistics of sequencing reads from the PacBio and ONT sequencing platforms.

| **Variant** | **Sequence platform** | **Total bases (Gb)** | **Total reads** | **Maxlength (bp)** | **N50 (bp)** |
| --- | --- | --- | --- | --- | --- |
| black | PacBio HiFi | 99.5 | 5,979,285‬ | 62,247 | 17,006 |
|  | ONT | 29.1 | 306,916 | 855,730 | 100,000 |
| golden | PacBio HiFi | 108.9 | 6,318,412 | 63,181 | 17,661 |
|  | ONT | 36.6 | 432,149 | 993,358 | 100,000 |

**Supplementary Table S4.** Statistical analysis of sequencing reads from Hi-C libraries.

| **Variant** | **Reads Length (bp)** | **Total Data (Gb)** |
| --- | --- | --- |
| black | 150 | 204.7 |
| golden | 150 | 215.6 |

**Supplementary Table S5.** Statistical analysis of the chromosome (Chr) details from available *C. carpio* genome assemblies

| **Black koi** | | | **Golden koi** | | | 1. ***carpio*（2014）[1]** | | ***C. carpio*（2021）[2]** | |
| --- | --- | --- | --- | --- | --- | --- | --- | --- | --- |
| **ID** | **Length (bp)** | **Telomere** | **ID** | **Length (bp)** | **Telomere** | **ID** | **Length (bp)** | **ID** | **Length (bp)** |
| Chr1_B3 | 50,413,000 | both | Chr1_B3 | 50,442,261 | both | NC_031697.1 | 15,297,981 | A1 | 39,504,823 |
| Chr2_A7 | 46,968,762 | both | Chr2_A7 | 46,235,364 | both | NC_031698.1 | 18,998,363 | A2 | 28,433,155 |
| Chr3_B7 | 43,952,210 | both | Chr3_B7 | 44,157,276 | both | NC_031699.1 | 20,567,433 | A3 | 39,349,182 |
| Chr4_B22 | 43,118,187 | both | Chr4_B22 | 42,004,672 | both | NC_031700.1 | 11,347,241 | A4 | 36,279,972 |
| Chr5_A5 | 41,970,081 | both | Chr5_A5 | 40,772,338 | both | NC_031701.1 | 20,922,272 | A5 | 39,981,485 |
| Chr6_A3 | 38,815,000 | both | Chr6_A3 | 39,872,000 | both | NC_031702.1 | 19,716,050 | A6 | 34,149,625 |
| Chr7_B5 | 37,447,714 | both | Chr7_B5 | 37,429,208 | both | NC_031703.1 | 14,157,913 | A7 | 47,223,169 |
| Chr8_A1 | 37,402,090 | both | Chr8_A1 | 36,475,294 | both | NC_031704.1 | 19,797,740 | A8 | 27,612,319 |
| Chr9_B1 | 36,901,822 | both | Chr9_B1 | 36,097,087 | both | NC_031705.1 | 24,322,175 | A9 | 32,520,448 |
| Chr10_A4 | 35,714,000 | both | Chr10_A4 | 34,919,000 | both | NC_031706.1 | 22,665,327 | A10 | 20,928,587 |
| Chr11_A9 | 33,821,014 | both | Chr11_B4 | 34,444,018 | both | NC_031707.1 | 19,054,100 | A11 | 26,868,042 |
| Chr12_B4 | 33,684,488 | both | Chr12_B16 | 33,064,571 | both | NC_031708.1 | 12,725,232 | A12 | 27,140,183 |
| Chr13_B9 | 33,647,563 | both | Chr13A2 | 32,942,991 | both | NC_031709.1 | 25,164,623 | A13 | 30,664,011 |
| Chr14_B16 | 32,784,000 | both | Chr14_B9 | 32,562,629 | both | NC_031710.1 | 18,393,167 | A14 | 30,475,081 |
| Chr15_A2 | 32,704,000 | both | Chr15_A9 | 32,185,841 | both | NC_031711.1 | 8,518,344 | A15 | 29,545,497 |
| Chr16_B2 | 31,521,000 | both | Chr16_B2 | 31,930,167 | both | NC_031712.1 | 20,606,094 | A16 | 23,572,709 |
| Chr17_A16 | 31,493,000 | both | Chr17_A16 | 31,354,801 | both | NC_031713.1 | 15,849,037 | A17 | 28,879,634 |
| Chr18_A6 | 30,934,006 | both | Chr18_A6 | 31,155,208 | both | NC_031714.1 | 22,437,665 | A18 | 30,158,143 |
| Chr19_B8 | 30,736,369 | both | Chr19_B18 | 30,978,080 | both | NC_031715.1 | 7,320,473 | A19 | 24,372,310 |
| Chr20_A18 | 30,716,200 | both | Chr20_B6 | 30,822,094 | both | NC_031716.1 | 16,766,033 | A20 | 27,589,077 |
| Chr21_B6 | 30,488,875 | both | Chr21_A13 | 30,482,000 | both | NC_031717.1 | 12,985,561 | A21 | 25,358,796 |
| Chr22_B18 | 29,983,000 | both | Chr22_B19 | 29,997,655 | both | NC_031718.1 | 8,395,091 | A22 | 22,237,253 |
| Chr23_A13 | 29,832,461 | both | Chr23_B13 | 29,981,361 | both | NC_031719.1 | 18,435,467 | A23 | 25,516,351 |
| Chr24_B19 | 29,827,000 | both | Chr24_A18 | 29,824,386 | both | NC_031720.1 | 12,449,181 | A24 | 25,855,823 |
| Chr25_B13 | 29,668,238 | both | Chr25_B8 | 29,746,740 | both | NC_031721.1 | 8,535,575 | A25 | 21,510,850 |
| Chr26_B17 | 29,297,142 | both | Chr26_B17 | 29,705,278 | both | NC_031722.1 | 16,349,257 | B1 | 40,474,758 |
| Chr27_A8 | 29,273,381 | both | Chr27_B20 | 29,128,730 | both | NC_031723.1 | 16,295,563 | B2 | 32,409,921 |
| Chr28_B12 | 29,187,518 | both | Chr28_B15 | 28,938,663 | both | NC_031724.1 | 22,269,040 | B3 | 48,440,497 |
| Chr29_B14 | 29,086,059 | both | Chr29_B14 | 28,724,817 | both | NC_031725.1 | 17,515,369 | B4 | 37,472,334 |
| Chr30_A20 | 29,005,768 | both | Chr30_A15 | 28,699,056 | both | NC_031726.1 | 18,521,859 | B5 | 38,117,132 |
| Chr31_B20 | 28,969,743 | both | Chr31_A12 | 28,637,671 | both | NC_031727.1 | 24,603,915 | B6 | 30,208,039 |
| Chr32_A17 | 28,600,000 | both | Chr32_B23 | 28,603,342 | both | NC_031728.1 | 26,100,072 | B7 | 47,159,336 |
| Chr33_B21 | 28,282,275 | both | Chr33_A20 | 28,373,762 | both | NC_031729.1 | 24,142,229 | B8 | 29,221,401 |
| Chr34_A11 | 28,253,490 | both | Chr34_A17 | 28,044,058 | both | NC_031730.1 | 18,891,512 | B9 | 34,063,200 |
| Chr35_B15 | 27,970,073 | both | Chr35_B21 | 27,926,853 | both | NC_031731.1 | 29,126,390 | B10 | 24,790,803 |
| Chr36_A12 | 27,771,507 | both | Chr36_B12 | 27,829,999 | both | NC_031732.1 | 22,739,634 | B11 | 24,701,381 |
| Chr37_A15 | 27,369,449 | both | Chr37_A14 | 27,612,537 | both | NC_031733.1 | 11,248,262 | B12 | 26,437,327 |
| Chr38_A14 | 27,349,779 | both | Chr38_A8 | 27,430,430 | both | NC_031734.1 | 27,918,166 | B13 | 31,841,649 |
| Chr39_B23 | 27,310,588 | both | Chr39_A19 | 26,911,978 | both | NC_031735.1 | 16,255,708 | B14 | 27,457,767 |
| Chr40_A19 | 27,134,710 | both | Chr40_A11 | 26,628,152 | both | NC_031736.1 | 23,490,330 | B15 | 29,072,104 |
| Chr41_A23 | 26,317,629 | both | Chr41_B24 | 25,481,683 | both | NC_031737.1 | 15,851,267 | B16 | 30,612,910 |
| Chr42_B24 | 25,811,403 | both | Chr42_B11 | 25,467,475 | both | NC_031738.1 | 13,570,270 | B17 | 28,741,886 |
| Chr43_B11 | 25,370,058 | both | Chr43_A21 | 25,327,000 | both | NC_031739.1 | 14,329,178 | B18 | 27,906,327 |
| Chr44_A21 | 25,185,100 | both | Chr44_A23 | 25,077,000 | both | NC_031740.1 | 15,901,873 | B19 | 32,052,705 |
| Chr45_B10 | 25,118,819 | both | Chr45_B10 | 24,756,280 | both | NC_031741.1 | 17,310,619 | B20 | 30,677,930 |
| Chr46_A24 | 24,624,792 | both | Chr46_A24 | 24,438,172 | both | NC_031742.1 | 7,828,959 | B21 | 20,763,676 |
| Chr47_A10 | 24,559,000 | both | Chr47_B25 | 23,667,752 | both | NC_031743.1 | 16,759,857 | B22 | 36,154,487 |
| Chr48_B25 | 23,630,748 | both | Chr48_A10 | 23,653,000 | left | NC_031744.1 | 22,470,700 | B23 | 27,667,832 |
| Chr49_A22 | 20,939,413 | both | Chr49_A22 | 21,489,453 | both | NC_031745.1 | 11,140,812 | B24 | 24,375,737 |
| Chr50_A25 | 20,120,747 | both | Chr50_A25 | 21,103,973 | both | NC_031746.1 | 7,653,411 | B25 | 24,466,304 |

**Supplementary Table S6.** Statistics of genome assemblies for two koi carps and two published common carps.

|  | **Black koi** | **Golden koi** | ***C. carpio* (2014) [1]** | ***C. carpio* (2021) [2]** |
| --- | --- | --- | --- | --- |
| Contig N50 (Mb) | 30.0 | 30.0 | 0.068 | 1.55 |
| Chromosome number | 50 | 50 | 50 | 50 |
| Gap number | 0 | 0 | 57,940 | 26,351 |
| BUSCO | 98.9% | 98.8% | 83.0% | 97.8% |

**Supplementary Table S7.** Statistical analysis of repeat sequences in both assembled genomes.

| **Combined REs** | | | | |
| --- | --- | --- | --- | --- |
|  | **black variant** | | **golden variant** | |
| **Type** | **Length (bp)** | **% in genome** | **Length (bp)** | **% in genome** |
| DNA | 413,466,352 | 26.4 | 400,246,721 | 25.8 |
| LINE | 88,292,667 | 5.6 | 92,069,318 | 5.9 |
| SINE | 12,834,464 | 0.8 | 16,058,846 | 1.0 |
| LTR | 80,898,909 | 5.2 | 80,151,619 | 5.2 |
| Other | 59,509 | 0.004 | 58,811 | 0.004 |
| Unknown | 94,108,686 | 6.0 | 98,020,412 | 6.3 |
| Total | 636,212,973 | 40.6 | 636,245,466 | 40.9 |

**Supplementary Table S8.** Statistics of predicted gene structures in the black koi carp genome.

| **Gene Set** | | **Gene Number** | **Average Transcript Length (bp)** | **Average CDS Length (bp)** | **Average Exons per Gene** | **Average Exon Length (bp)** | **Average Intron Length (bp)** |
| --- | --- | --- | --- | --- | --- | --- | --- |
| **Homolog** |  |  |  |  |  |  |  |
| *Danio rerio* | | 63,656 | 8,969.7 | 1,404.1 | 6.3 | 222.7 | 1,425.8 |
| *Oryzias latipes* | | 67,133 | 6,587.3 | 1,151.6 | 4.9 | 233.7 | 1,383.6 |
| *Ctenopharyngodon idellus* | | 64,544 | 9,598.2 | 1,359.1 | 6.5 | 208.5 | 1,492.8 |
| *Cyprinus carpio* | | 43,961 | 14,696.6 | 1,638.3 | 9.5 | 172.6 | 1,537.7 |
| *Sinocyclocheilus anophthalmus* | | 33,523 | 22,332.0 | 1,614.2 | 9.1 | 176.8 | 2,548.1 |
| **Transcriptome** |  | 60,959 | 4,964.9 | 1,506.4 | 4.5 | 331.5 | 975.8 |
| **MAKER** | | 55,023 | 15,771.5 | 1,834.7 | 9.5 | 193.8 | 1,571.9 |

**Supplementary Table S9.** Statistics of predicted gene structures in the golden koi carp genome.

| **Gene Set** | | **Gene Number** | **Average Transcript Length (bp)** | **Average CDS Length (bp)** | **Average Exons per Gene** | **Average Exon Length (bp)** | **Average Intron Length (bp)** |
| --- | --- | --- | --- | --- | --- | --- | --- |
| **Homolog** |  |  |  |  |  |  |  |
| *Danio rerio* | | 63,233 | 9,013.5 | 1,408.0 | 6.3 | 223.6 | 1,436.2 |
| *Oryzias latipes* | | 66,447 | 6,596.6 | 1,149.2 | 4.9 | 234.7 | 1,397.8 |
| *Ctenopharyngodon idellus* | | 50,586 | 9,412.6 | 1,362.8 | 6.4 | 214.3 | 1,502.1 |
| *Cyprinus carpio* | | 43,926 | 14,668.6 | 1,639.3 | 9.5 | 172.6 | 1,533.4 |
| *Sinocyclocheilus anophthalmus* | | 33,288 | 22,452.4 | 1,611.4 | 9.1 | 177.3 | 2,576.8 |
| **Transcriptome** |  | 67,899 | 6,226.8 | 1,942.8 | 5.3 | 370.2 | 1,008.4 |
| **MAKER** | | 54,569 | 16,005.4 | 1,847.7 | 9.5 | 194.5 | 1,574.0 |

**Supplementary Table S10.** Summary of functional annotations.

|  | **black variant** | | **golden variant** | |
| --- | --- | --- | --- | --- |
| **Database** | **Number** | **Percentage (%)** | **Number** | **Percentage (%)** |
| Total | 55,023 | 100% | 54,569 | 100% |
| InterPro | 45,900 | 83.42% | 41,567 | 76.17% |
| KEGG | 49,336 | 89.66% | 46,165 | 84.60% |
| SwissProt | 52,324 | 95.09% | 52,850 | 96.85% |
| TrEMBL | 45,052 | 81.88% | 48,888 | 89.59% |
| Overall | 52,855 | 96.06% | 52,975 | 97.08% |

**Supplementary Table S11.** NCBI accession numbers of the six representative genomes for phylogenetic analysis.

| **Species** | **accession number** | **Haploidic Chromosome Number** |
| --- | --- | --- |
| *Danio rerio* | GCF_000002035.6 | 25 |
| *Ctenopharyngodon idella* | GCA_019924925.1 | 24 |
| *Gobiocypris rarus* | GCA_018491645.1 | 25 |
| *Sinocyclocheilus anophthalmus* | GCA_018155175.1 | 48 |
| *Cyprinus carpio var. Songpu* | GCA_018340385.1 | 50 |
| *Oryzias latipes* | GCF_002234675.1 | 24 |

**Supplementary Table S12.** Summary of the best-hit gene pairs between a predicted ancestor and seven representative Cypriniformes (including two koi carps).

| **Species** | **best-hit gene pairs** |
| --- | --- |
| *Danio rerio* | 8,587 |
| *Gobiocypris rarus* | 10,630 |
| *Ctenopharyngodon idella* | 12,383 |
| *Cyprinus carpio var. Songpu* | 13,823 |
| *Sinocyclocheilus anophthalmus* | 11,248 |
| *Cyprinus carpio var. koi black* | 12,328 |
| *Cyprinus carpio var. koi golden* | 12,260 |

1. Xu P, Zhang X, Wang X, Li J, Liu G, Kuang Y, et al. Genome sequence and genetic diversity of the common carp, Cyprinus carpio. Nat Genet. 2014;46 11:1212-9. doi:10.1038/ng.3098.

2. Li JT, Wang Q, Huang Yang MD, Li QS, Cui MS, Dong ZJ, et al. Parallel subgenome structure and divergent expression evolution of allo-tetraploid common carp and goldfish. Nat Genet. 2021;53 10:1493-503. doi:10.1038/s41588-021-00933-9.
