# Supplementary material for: Telomere-to-telomere chromosome-scale genome assemblies of black and golden koi carp variants support construction of an ancient karyotype of Cypriniformes
Source: Gigascience. 2025 Jul 26;14:giaf073. doi: 10.1093/gigascience/giaf073 (PMC13223729; doi:10.1093/gigascience/giaf073)
Supplement: giaf073_GIGA-D-24-00549_original_submission [file giaf073_giga-d-24-00549_original_submission.pdf]

GigaScience

Telomere-to-telomere chromosome-scale genome assemblies of black and golden koi carp variants support construction of an ancient karyotype of Cypriniformes

--Manuscript Draft--

**Manuscript Number:**	GIGA-D-24-00549
**Full Title:**	Telomere-to-telomere chromosome-scale genome assemblies of black and golden koi carp variants support construction of an ancient karyotype of Cypriniformes
**Article Type:**	Data Note
**Funding Information:**	Key Technologies Research and Development Program (2023YFE0205100) — professor Chao Bian
**Abstract:**	Background<br><br>Koi carps (Cyprinus carpio var. koi) has served as an important model for studying fish pigmentation, due to its precious variability in skin color. It is also an excellent model to study chromosome changes after undergoing whole genome duplication.<br><br>Results<br><br>We constructed two telomere-to-telomere chromosome-level genome assemblies for black and golden koi carp variants by integration of MGI, PacBio HiFi, ONT, and Hi-C sequencing technologies. Haplotypic genomes of the black and golden koi carps covered 50 chromosomes with 100 and 99 telomeres, respectively. BUSCO results validated 98.9% and 98.8% completeness of both genomes. A total of 55,023 and 54,569 protein-coding genes were respectively annotated in the assembled genomes, with 96.06% and 97.08% were assigned with functional roles. We also estimated that the black and golden koi carp genomes contain 636.2 Mb and 636.2 Mb of repetitive sequences, accounting for 40.6% and 40.9% of their whole genomes. Based on a phylogenetic analysis and divergence time estimation, we predicted that the two koi carps split from each other about 8.1 million years ago. By analyzing chromosome sequences of seven representative Cypriniformes species, we constructed an ancient karyotype of 25 ancestral chromosomes, which revealed nine major chromosomal rearrangements in the examined species.<br><br>Conclusions<br><br>These extremely high-quality data offer valuable genomics resources, which support an ancient karyotype of Cypriniformes. Both T2T genome assemblies can also be used as good references for in-depth genetic and evolutionary investigations on diverse skin coloration in koi and other Cypriniformes species.
**Corresponding Author:**	Chao Bian, ph.D<br>Shenzhen University<br>shenzhen, CHINA
**Corresponding Author Secondary Information:**	
**Corresponding Author's Institution:**	Shenzhen University
**Corresponding Author's Secondary Institution:**	
**First Author:**	Chao Bian, ph.D
**First Author Secondary Information:**	
**Order of Authors:**	Chao Bian, ph.D
	Rujingwen Huan
	Qiong Shi

Powered by Editorial Manager® and ProduXion Manager® from Aries Systems Corporation

Order of Authors Secondary Information:	
Additional Information:	
Question	Response
Are you submitting this manuscript to a special series or article collection?	No
**Experimental design and statistics**<br><br>Full details of the experimental design and statistical methods used should be given in the Methods section, as detailed in our Minimum Standards Reporting Checklist. Information essential to interpreting the data presented should be made available in the figure legends.<br><br>Have you included all the information requested in your manuscript?	Yes
**Resources**<br><br>A description of all resources used, including antibodies, cell lines, animals and software tools, with enough information to allow them to be uniquely identified, should be included in the Methods section. Authors are strongly encouraged to cite Research Resource Identifiers (RRIDs) for antibodies, model organisms and tools, where possible.<br><br>Have you included the information requested as detailed in our Minimum Standards Reporting Checklist?	Yes
**Availability of data and materials**<br><br>All datasets and code on which the conclusions of the paper rely must be either included in your submission or deposited in publicly available repositories (where available and ethically appropriate), referencing such data using a unique identifier in the references and in the "Availability of Data and Materials" section of your manuscript.	Yes

Have you have met the above requirement as detailed in our Minimum Standards Reporting Checklist?	
GigaScience has policies and guidelines in place for the use of generative AI-writing tools such as ChatGPT. If you have used such writing tools to assist with writing the manuscript this must be declared and cited in the text. Authors should not list AI-writing tools and other AI-assisted technologies as an author or co-author and should acknowledge that they are fully responsible for text generated or refined by AI-writing tools.<p><br><br>A summary of use (particularly in the introduction or among methods) needs to be included at the end of the paper, and the outputs should also be included as a supplementary file hosted in GigaDB or other open repositories. Please read our guidelines for more information. <p><br><br>By submitting to GigaScience, you are aware of the journal's AI-writing tools policy, and if you have declared use of such tools below, you have acknowledged this where appropriate in your manuscript and have made a summary of use and outputs available. <p><br><b>AI-assisted writing tools have been used in the preparation of this manuscript?	No

Manuscript Click here to access/download;Manuscript;manuscript 20241204.docx

Telomere-to-telomere chromosome-scale genome assemblies of black and golden koi carp variants support construction of an ancient karyotype of Cypriniformes

Chao Bian[1,2,*], Rujingwen Huan[1], Qiong Shi[1,2,*]

[1]Laboratory of Aquatic Genomics, College of Life Sciences and Oceanography, Shenzhen University, Shenzhen, Guangdong 518057, China

[2]Shenzhen Key Lab of Marine Genomics, BGI Academy of Marine Sciences, BGI Marine, Shenzhen, Guangdong 518081, China

* Correspondence address: Qiong Shi, Bldg A6 Room 324, College of Life Sciences and Oceanography, Shenzhen University, Shenzhen, Guangdong 518057, China. E-mail: shiqiong@szu.edu.cn or shiqiong@genomics.cn;

Chao Bian, Bldg A6 Room 510, College of Life Sciences and Oceanography, Shenzhen University, Shenzhen, Guangdong 518057, China. E-mail: bianchao@szu.edu.cn

Abstract

Background: Koi carp (*Cyprinus carpio var. koi*) has served as an important model for studying fish pigmentation, due to its precious variability in skin color. It is also an excellent model to study chromosome changes after undergoing whole genome duplication.

Results: We constructed two telomere-to-telomere chromosome-level genome assemblies for black and golden koi carp variants by integration of MGI, PacBio HiFi, ONT, and Hi-C sequencing technologies. Haplotypic genomes of the black and golden koi carps covered 50 chromosomes with 100 and 99 telomeres, respectively. BUSCO results validated 98.9% and 98.8% completeness of both genomes. A total of 55,023 and 54,569 protein-coding genes were respectively annotated in the assembled genomes. Of them, 96.1% and 97.1% were assigned with functional roles. We also estimated that the black and golden koi carp genomes contain 636.2 Mb and 636.3 Mb of repetitive sequences, accounting for 40.6% and 40.9% of their whole genomes.

Based on a phylogenetic analysis and divergence time estimation, we predicted that these two koi carps split from each other about 8.1 million years ago. By analyzing chromosome sequences of seven representative Cypriniformes species, we constructed an ancient karyotype of 25 ancestral chromosomes, which revealed nine major chromosomal rearrangements in the examined species.

Conclusions: These extremely high-quality data offer valuable genomics resources, which support to predict an ancient karyotype of Cypriniformes. Both T2T genome assemblies can also be used as good references for in-depth genetic and evolutionary investigations on diverse skin coloration in koi and other Cypriniformes species.

Key words: Koi carp variant, whole genome sequencing, telomere-to-telomere, genome assembly, ancient karyotype

Data Description

Background information

Koi carp (*Cyprinus carpio var. koi,* NCBI Taxonomy ID: 1,499,333), a member of the Cyprinidae family, has been one of the most popular ornamental fish species due to its diverse coloration. It also serves as an important model for studying the skin coloration and evolutionary adaptation of fish. As we know, the coloration in fish, especially in ornamental species, plays a crucial role for camouflage, signaling, mate selection, and overall market value [1], making it a key trait for selective breeding and aquaculture improvement. Compared to other vertebrates, fishes have more categories of pigment cells, resulting in complex skin coloration patterns. However, this makes it more challenging for researchers to investigate related pigmentation mechanisms.

In recent years, lots of studies have focused on understanding the genetic and molecular mechanisms behind koi fish coloration. For example, miR-430 was proved to regulate genes involved in carotenoid metabolism and pigmentation in koi carp, revealing important impacts on coloration by modulating chromatophore density and pigment synthesis [2]. Deep transcriptome analyses of koi carp have also provided valuable insights into the early developmental processes. Several key genes, such as *pax3, pax7* and *gch2*, were validated to play important roles in pigmentation and

neural crest development during early embryogenesis [3].

On the other hand, previous studies have reported that common carp is an allotetraploid species, and two diploid progenitors split about 23 million years ago (Mya) and merged about 12.4 Mya [2]. However, many important issues, such as how the ancestral chromosomes of teleost fishes evolved into those of Cypriniformes, and what changes occurred after the whole-genome duplication of ancestral chromosomes of Cypriniformes, remain largely unknown at present.

In our current study, we presented two telomere-to-telomere (T2T) chromosome-level genome assemblies of black and golden koi carp variants, which were fulfilled through a combination of MGI, PacBio HiFi, Oxford Nanopore ultra-long (ONT), and Hi-C sequencing technologies. Both assembled genomes contained 50 chromosomes with 100 and 99 telomeres, respectively. They exhibited extremely high completeness. We further investigated the chromosomal evolutionary history of the Cypriniformes lineage by conducting an ancestral chromosome analysis, which led to identification of 25 ancestral chromosomes and 9 major chromosomal rearrangement events. In summary, our present work contributed to understanding the chromosome evolution of Cypriniformes and laid a solid foundation for in-depth investigations on molecular characteristics of koi pigmentation and coloration. Both valuable genome assemblies will serve as important references for practical breeding (such as development of molecular markers for gender identification) and future theory research in diverse colored variants.

Methods

Sample collection, DNA extraction, and whole genome sequencing

One female black and one female golden koi carps were collected from a local aquaculture farm in Guangzhou city, Guangdong Province, China. We pooled muscle samples separately from both koi carps, and extracted genomic DNA (gDNA) from both samples for whole genome sequencing by MGI, Pacbio HiFi, ONT and Hi-C technologies. Our study protocol was approved by the Laboratory Animal Ethics

Committee of Shenzhen University.

For the gDNA sequencing, libraries with an insert size of 500 bp were constructed by using a MGIEasy UDB Universal Library Prep Set following the manufacturer's instructions (MGI Tech Co. Ltd., China, RRID:SCR_017981). These libraries were subsequently sequenced on a DNBSEQ-T7 machine (MGI). The HiFi long-read libraries were prepared by using a SMRTbell Express Template Prep Kit 2.0 (Pacific Biosciences, USA), following the standard protocols as the manufacturer recommended. And then HiFi long-read data we generated through sequencing on a PacBio Sequel II platform (Pacific Biosciences, RRID:SCR_017990) [4]. CCS software (SMRT Link v9.0) was employed to generate consensus sequences [4]. Both ONT ultra-long libraries were built by an Oxford Nanopore SQK-ULK001 kit according to the instructions from the manufacturer (Oxford Nanopore Technologies, UK), which were then sequenced on a PromethION flow cell (Oxford Nanopore Technologies, RRID:SCR_017987). These ONT reads were corrected by NECAT v200221 software (RRID: SCR_025350) with default parameters [5]. Both Hi-C libraries were constructed using a GrandOmics Hi-C Kitand DpnII enzyme according to the manufacturer's standard protocol (GrandOmics, China). Sequencing was conducted on an Illumina NovaSeq platform (Illumina, USA; RRID: SCR_016387). All these sequencing reads were integrated for genome assembling and chromosome anchoring.

Total RNA samples were prepared from muscle, skin, liver and heart tissues of both koi carps using a TRIZOL Kit (Invitrogen, USA) in accordance with the manufacturer's instructions. Integrity and quality of the extracted RNA were evaluated using an Agilent 2100 Bioanalyzer (Agilent Technologies, USA; RRID: SCR_018043), and only those samples with an RNA Integrity Number (RIN) over 7.0 were selected for subsequent library preparation. cDNA libraries were constructed using DNA nanoballs (DNBs) in accordance with the manufacturer's protocol for a DNBSEQ platform (MGI; RRID:SCR_017981). These libraries were sequenced on a MGISEQ-2000 platform (MGI) with a paired-end model (150 bp in length).

Genome size prediction, genome assembling, telomere identification, and assembly quality evaluation

Genome sizes of the black and golden koi carps were estimated by using a 17-mer frequency distribution analysis [6] of cleaned MGI data with the insert size of 500 bp. They were calculated according to the following equation: genome size=k-mer number/the expectation of k-mer depth.

The initial genome assemblies were completed by using Hifiasm v0.19.8 (detailed parameters: -t 16 --n-hap 4 --hg-size 1520m; RRID: SCR_021069) with the PacBio HiFi and ONT reads [7]. The Hi-C sequencing reads were aligned onto the above assembled contigs by using Bowtie 2 (parameters:–very-sensitive -L 30–score-min L, −0.6, −0.2–end-to-end –reorder; RRID:SCR_016368) [8]. YaHs v1.0 (RRID: SCR_022965) [9] was then employed with default parameters to compute the chromosomal linkage information based on these alignment results. These alignments were subsequently used with Juicer v1.5 (parameters: chr_num 30;RRID:SCR_017226) [10] and 3D-DNA v170123 (parameters: -m haploid -r 2; RRID:SCR_017227) [11] to anchor the contigs onto primary chromosomes. Juicebox v1.11.08 (RRID:SCR_021172) [12] was further utilized for refining the assembly. Furthermore, the primary chromosome-level genome assemblies of black and golden koi carps contain 7 and 8 gaps, respectively. To achieve a gap-free and T2T level, LR_GapCloser v1.0 (parameters: -t 35 -m 1000000 -v 10000; RRID:SCR_016194) [13] and TGS-GapCloser v1.0.1 (parameter:-min_match 2000; RRID:SCR_017633) [14] were sequentially applied to fill those gaps within both genome assemblies. Centromere and telomere sequences were identified using the QuarTeT software (RRID:SCR_025258) [15].

For quality evaluation of both assemblies, a Benchmarking Universal Single-Copy Orthologs (BUSCO; RRID:SCR015008) [16] evaluation was performed to predict completeness. We also mapped PacBio HiFi and ONT reads onto both genome assemblies by using Minimap2 (RRID:SCR018550) [17] to conduct more assembling correction. The quality value (QV) was estimated by Merqury-20200430 software (RRID: SCR_022964) [18] with recommended 20 kmer.

Repeat element annotation

Repeat elements (REs) in both genomes were identified through combination of *de novo* and homology-based methods. For the *de novo* prediction, RepeatModeler v1.0.8 (RRID:SCR_015027) [19] and LTR_Finder v1.0.6 (RRID:SCR_015247) [20] were applied to detect different types of REs. Then both of the new repeat libraries were generated by integrating RepeatMasker v4.0.623 (RRID:SCR_012954) [21] and Repbase TE v21.01 (RRID:SCR_021169) [22]. Tandem repeats were detected by using Tandem Repeats Finder (parameters: 2 7 7 80 10 50 2000 -d -h; RRID:SCR_022193) [23]. With the new repeat libraries, RepeatProteinMask v4.0.623 [21] and RepeatMasker v4.0.623 (RRID:SCR_012954) [21] were employed to identify repetitive sequences.

Gene prediction and functional annotation

To annotate protein-coding genes, we integrated homology alignment and transcriptome data to generate non-redundant sets of protein-coding genes for both black and golden koi carps. For the homology-based annotation, protein sequences of five representative species, including zebrafish (*Danio rerio*), medaka (*Oryzias latipes*), grass carp (*Ctenopharyngodon idellus),* common carp (*Cyprinus carpio var. Songpu),* golden-line barbel fish *(Sinocyclocheilus anophthalmus)*, were downloaded from the NCBI (RRID:SCR_006472) database and aligned them to our assembled genomes using TBLASTN (e-value 10^{-5}; RRID:SCR_011822) [24]. Based on these TBLASTN alignments, GeneWise v2.2.0 (parameters: --blast_eval 1e-5 --align_rate 0.5 --extend_len 500; RRID:SCR_015054) [25] was employed to predict gene structures. Transcriptome reads were mapped onto the genomic components using HISAT2 (RRID:SCR_015530) [26], and the transcriptome annotation sets were generated using Cufflinks v2.2.1 (RRID:SCR_014597)[27].

MAKER (max_dna_len=300000, min_contig=500, pred_flank=500, AED_threshold=1, split_hit=30000, single_exon=1, single_length=250, tries=2; RRID:SCR_005309) [28] was then utilized to integrate gene sets generated from the two methods to produce the final non-redundant protein-coding gene sets. Functional

annotation was conducted by aligning them with five public databases, including SwissProt (RRID:SCR_021164) [29], TrEMBL [30], KEGG (RRID:SCR_012773) [31], Gene Ontology (GO, RRID:SCR_002811) [32] and InterPro (RRID:SCR_005829) [33].

Gene families, phylogenetic tree construction, and divergence time estimation

Chromosome-level genomes of five representative species of Cypriniformes (including *Danio rerio, Ctenopharyngodon Idella, Gobiocypris rarus, Sinocyclocheilus anophthalmus, Cyprinus carpio var. Songpu*) and *Oryzias latipes* were downloaded from NCBI for phylogenetic and divergence time analyses. For the phylogenetic analysis, BLASTP (RRID:SCR_001010) [34] and OrthoMCL(RRID:SCR_007839) [35] were performed for protein sequence alignment and gene family clustering. All the single-copy orthologous genes were aligned by using MUSCLE v3.8.31(RRID:SCR_011812) [36] for all examined genomes. Then, Gblocks (RRID:SCR_015945) [37] was used to obtain conservative multi-sequence alignments. Finally, we employed PhyML v4.9 (RRID:SCR014932) [38] to construct a phylogenetic tree using the maximum likelihood method. Species divergence times were estimated using MCMCTREE in PAML v4.9 (RRID:SCR014932) [38]. A divergence time point from the TimeTree website (http://www.timetree.org/, RRID:SCR021162), 140–170 Mya between *Oryzias latipes* and *Danio rerio*, was employed to calibrate divergence times.

Construction of ancestral chromosomes

Proteinortho v6.0.36 (RRID:SCR_024177) [39] was employed to obtain a single-copy protein set from the protein sequences of seven representative species. This single-copy protein set was then concatenated into a supergene. A phylogenetic tree was constructed using the same strategy as described above. Several previous studies reconstructed the13 pairs of ancestral chromosomes (a~m) of teleost, and concluded that eight major rearrangements occurred after the third round of whole genome duplication (3R WGD) [40-42]. Thus, the most recent common ancestor (TMRCA) of teleost was putatively considered with 24 ancestral chromosomes [40-42].

Protein dataset of each representative species of Cypriniformes was aligned to the protein set of predicted ancestral teleost by using BLASTP (with an E-value threshold of 1e-10; RRID:SCR_001010). Those identified matching regions with same color number less than 20 per chromosome were filtered out. Finally, chromosome rearrangements, including chromosome fissions, fusions and translocations, were predicted. We finally applied SVG in Perl to visualize the predicted karyotype of the ancestor of Cypriniformes. These gene sequence fragments that were homologous to the ancestral chromosomes were marked correspondingly with the same color type.

Results

Summary of the sequencing reads and both genome assemblies

Through the *k*-mer analysis of MGI data (78.6 and 73.7 Gb, respectively; **Supplementary Table S1**), we estimated genome sizes of both black and golden koi carp variants were around 1.53 Gb (**Figure 1, Supplementary S2**). We also sequenced both koi genomes using the PacBio Sequel II and the ONT platforms. A total of 99.5 and 108.9 Gb of PacBio Sequel long reads, and 29.1 and 36.6 Gb of ONT ultra-long reads (**Supplementary Tables S3**) were respectively obtained. The initial genomes of black and golden koi carps were assembled to be 1.57 Gb and 1.55 Gb in length (**Table 1**), and their contig N50 reached 30.1 Mb and 30.0 Mb, respectively. Meanwhile, we obtained 204.7 Gb and 215.6 Gb of Hi-C reads for black and golden koi carps (**Supplementary Tables S4**). Through Hi-C read mapping and contig anchoring, we constructed 50 chromosomes with 7-8 gaps in the assembled genomes. After gap closing, both final assemblies reached a high-quality gap-free chromosome level.

The total 50 chromosome sequences of black and golden koi carps are up to 1.55 and 1.54 Gb, respectively (see detailed length of each chromosome in **Supplementary Tables S5**), accounting for about 98.9% and 99.3% of the assembled contigs. Interestingly, the black koi carp genome contained completely 100 telomeres,

while golden koi carp genome has 99 telomeres (only one of its chr48 was not identified; **Figure 2b**). The BUSCO results showed 98.9% and 98.8% completeness (**Table 1**), respectively. The ONT and HiFi reads were completely mapped onto both assembled genomes, and the MGI reads showed high mapped rates (97.0% and 97.6%, respectively). Merqury indicated that the quality values of both assemblies are 46.8. All above evaluations confirmed the high quality and completeness of our genome assemblies for both black and golden koi carps.

Repeat element and gene annotation data

Repeat annotation was performed using both *de novo* and homology-based methods. Eventually, we predicted that the black and golden koi carp genomes contain 636.2 and 636.3 Mb of repetitive sequences, accounting for 40.6% and 40.9% of their genomes respectively (**Supplementary Tables S6)**.

Gene prediction was carried out using an integrated approach of homology-based and transcriptome-based annotations. A total of 55,023 and 54,569 protein-coding genes, with an average of 15.8 and 16.0 kb in mRNA length (**Supplementary Tables S7 and S8**), were annotated respectively. Among them, over 96.1% and 97.1% of the predicted genes were assigned with at least one functional role from the five searched databases (including SwissProt, TrEMBL, KEGG, GO, and InterPro; **Supplementary Table S9**).

Predicted divergence times and ancestral chromosomes of Cypriniformes

We predicted that the divergence time between *C. carpio var. Songpu* and *C. carpio var. koi* was approximately 14.1 Mya, and both koi carps split from each other about 8.1 Mya (**Figure 3**). A total of 25 ancestral chromosomes were predicted with nine major chromosomal rearrangements in Cypriniformes after divergence from the common ancestor of teleost, including 2 fusions, 1 fission, 4 chromosomal translocations and 2 complex chromosomal rearrangements (see more details in **Figure 4A**). We also constructed a phylogenetic tree for six representative Cypriniformes species with different karyotypes (n = 24, 25, 48 and 50), and analyzed detailed variances within these examined species (**Figure 4B-G**). Total numbers of the

best-hit gene pairs between the predicted ancestor and seven representative Cypriniformes species (including two koi carps) were summarized for comparison (**Supplementary Table S11**).

These chromosomal rearrangements are conserved in the examined Cypriniformes species with hyplotypic 25 or 50 chromosomes. Two diploid fishes with a total number of 25 chromosomes, including rare minnow (*Gobiocypris rarus*; **Figure 4C**) and zebrafish (*Danio rerio*; **Figure 4D**), have similar chromosome structure to the ancestral Cypriniformes chromosomes. Two tetraploid fishes with a total number of 50 chromosomes, both purse red carp (*Cyprinus carpio wuyuanensis*) and koi carp (*Cyprinus carpio var. koi*), experienced one more WGD event, but their karyotypes were majorly conserved (**Figure 4E-F**). Only a small chromosomal fusion was found in koi carp (**Figure 4E**). Some special chromosomal fusions occurred in fishes with 24 and 48 chromosomes. For instance, the diploid grass carp (*Ctenopharyngodon Idella*; n=24) experienced one chromosome fusion (**Figure 4B**), while the tetraploid *Sinocyclocheilus* species (n=48) experienced two chromosome fusions (**Figure 4G**) after the *Sinocyclocheilus*- Lineage WGD. Interestingly, one paired ancestral chromosomes were fused into two other chromosomes from different origins (**Figure 4G**). It seems that the ancestors of diploid and tetraploid Cypriniformes were completely separated before their independent fusion events.

Conclusion

We at the first time reported two gap-free T2T genome assemblies for black and golden koi carps, by assembling the MGI, HiFi, ONT, and Hi-C sequencing reads. In total, 50 chromosomes (n=50) with 99-100 telomeres were constructed. We annotated 55,023 and 54,569 protein-coding genes in both assembled genomes. We also predicted the divergence time among seven representative Cypriniformes species, and reconstructed 25 ancestral chromosomes with nine major chromosomal rearrangements for Cypriniformes after divergence from the common ancestor of teleost, including 2 fusions, 1 fission, 4 chromosomal translocations, and 2 complex

chromosomal rearrangements. These findings offer valuable genetic resources for in-depth investigations on genetic and evolutionary mechanisms underlying diverse skin coloration in koi carps and other Cypriniformes species.

Table 1. Statistics of the genome assembly and annotation results of black and golden koi carps.

**Parameter**	**Black variant**	**Golden variant**
MGI reads (Gb)	78.6	73.7
HiFi reads (Gb)	99.5	108.9
ONT reads (Gb)	29.1	36.6
Hi-C reads (Gb)	204.7	215.6
Genome size (Mb)	1,568.0	1,553.7
Chromosome N50 (Mb)	30.0	30.0
Gap number	0	0
Telomere number	100	99
Merqury QV	46.8	46.8
BUSCO value	98.9%	98.8%
Repeat ratio	40.6	40.9
Gene number	55,023	54,569
Average gene length (bp)	15,771.5	16,005.4
Functional gene number	52,855	52,975

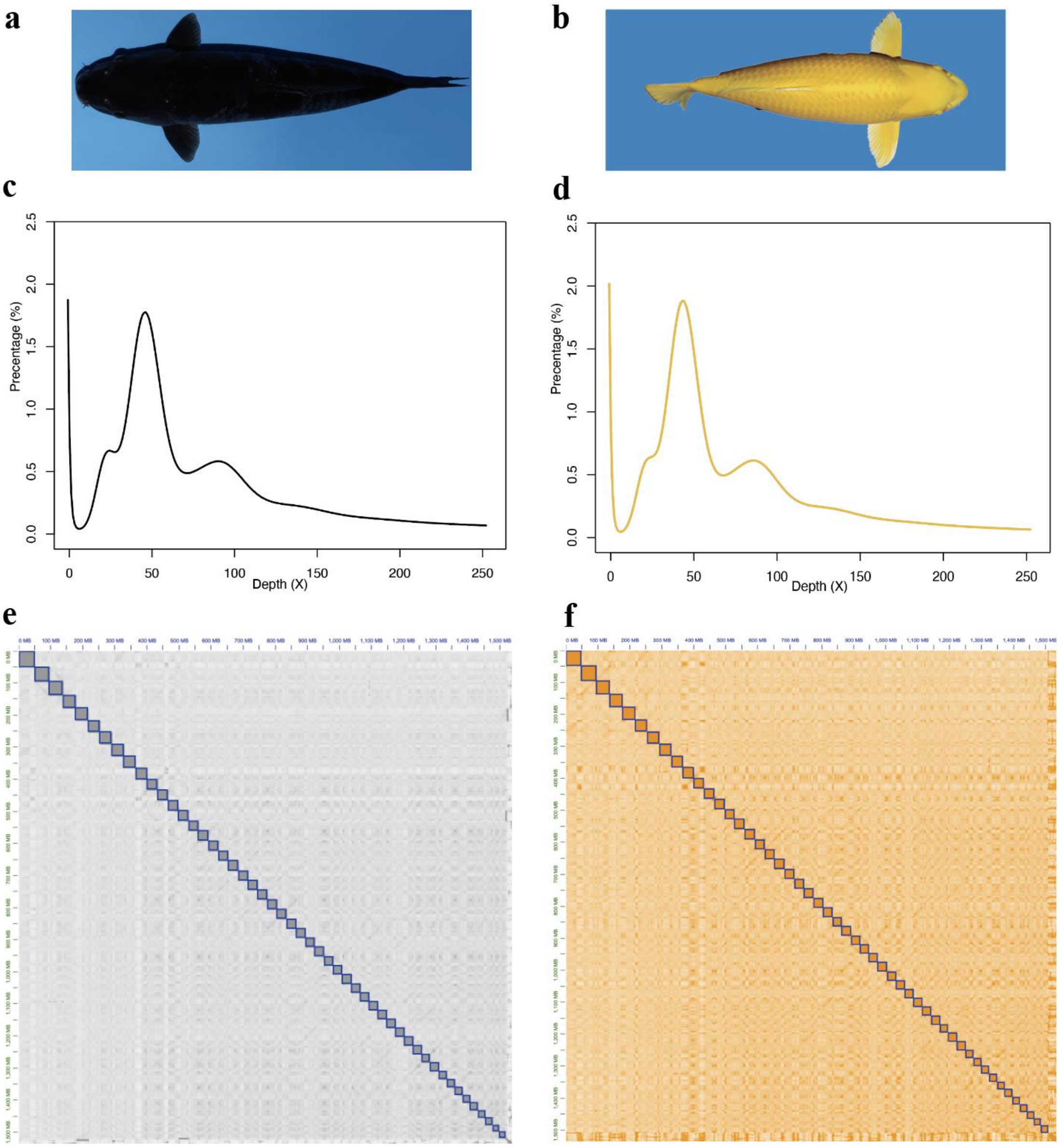

Figure 1. The black and golden koi carps and their whole-genome sequencing. (**a-b**) Images of the sequenced black and golden variants. (**c-d**) *k*-mer distribution for both genome sequencing. (**e-f**) chromosome heatmaps of Hi-C data for black and golden koi carps, respectively.

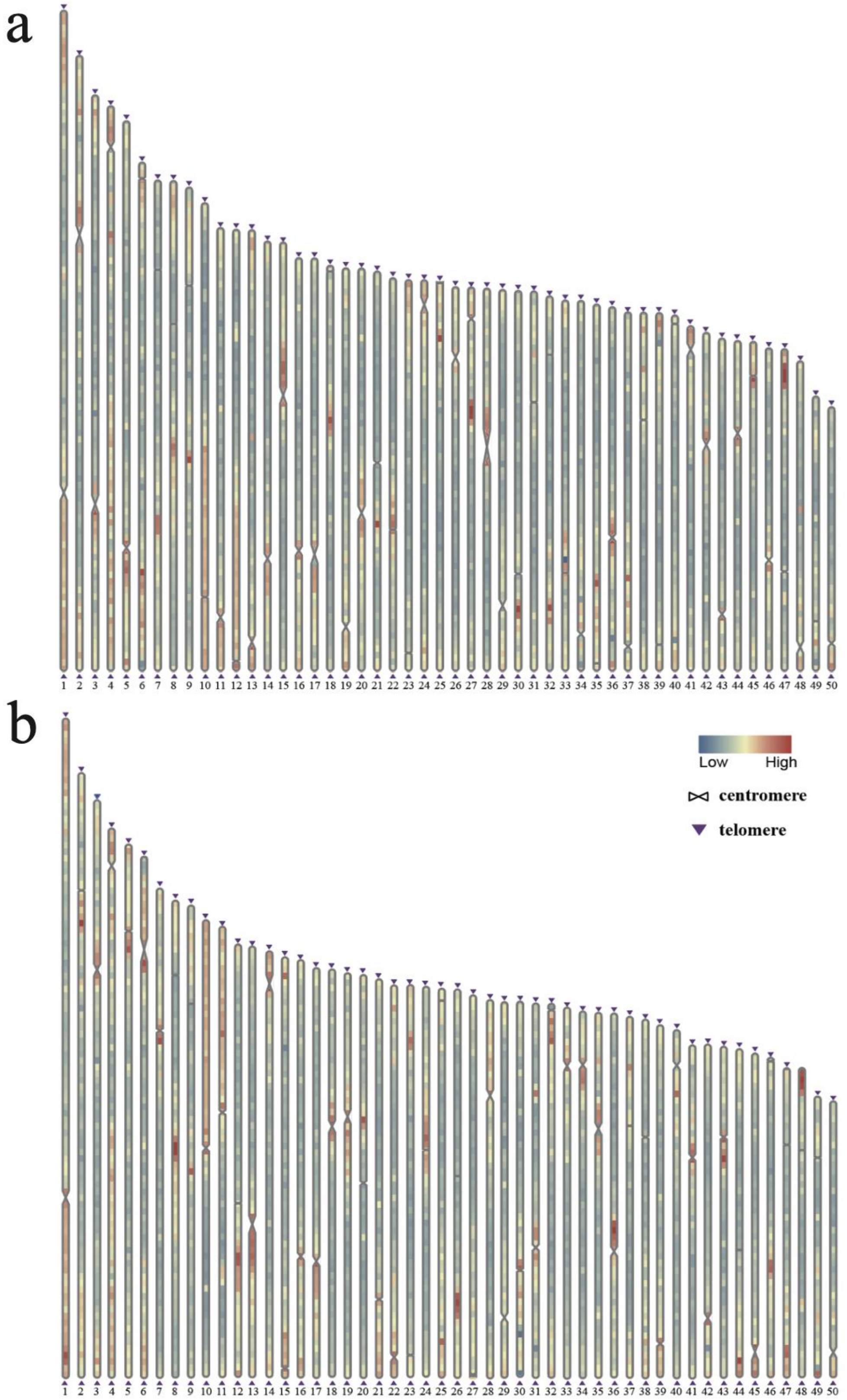

Figure 2. Genome-wide localization of centrosomes and telomeres. Details on each chromosome of black (**a**) and golden (**b**) koi carps are provided for comparison.

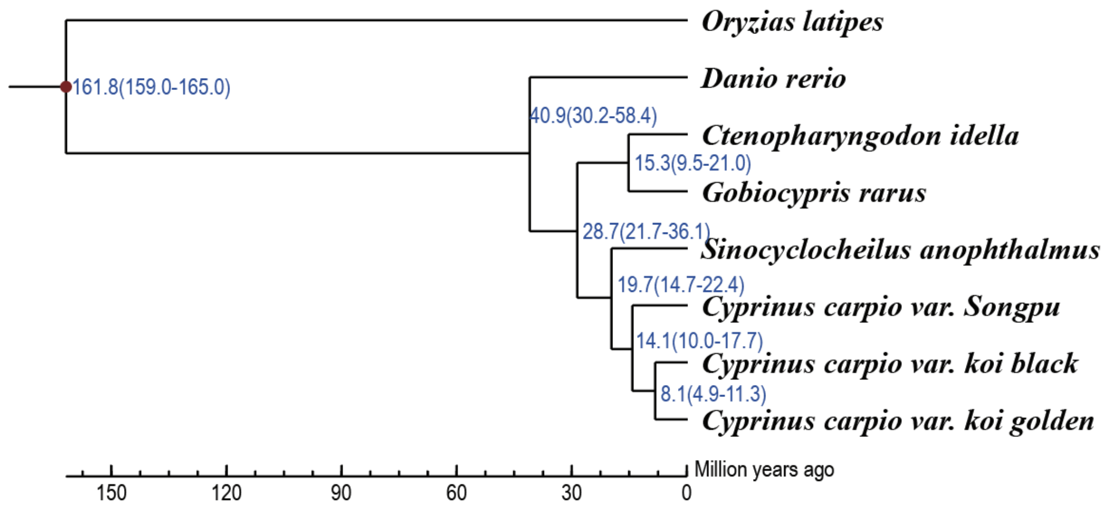

Figure 3. Divergence time tree of seven representative Cypriniformes species. Medaka (*Oryzias latipes*) was set as the outgroup. Blue numbers represent the estimated periods of divergence times. The red dot marks the reference period of divergence time from the TimeTree (http://www.timetree.org/).

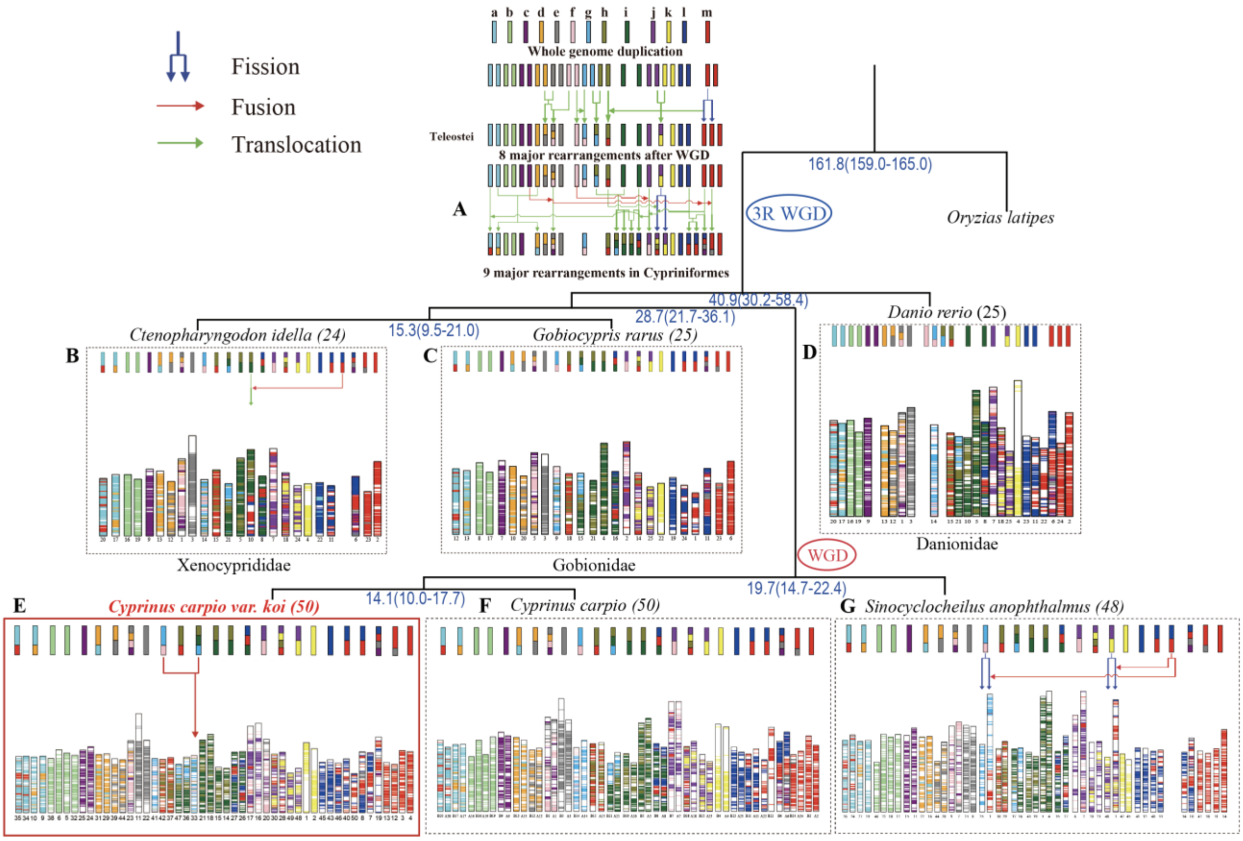

Figure 4. Evolution of chromosome karyotypes in Cypriniformes. Thirteen color bars represent teleost ancestor chromosomes a~m. The blue arrows indicate fission events, the red arrows indicate fusion events, and the green arrows indicate chromosomal translocation events. Red boxes mark new chromosomes after chromosomal fusions. Examined species include the ancestor of Cypriniformes (**A**), *Ctenopharyngodon Idella* (**B**), *Gobiocypris rarus* (**C**), *Danio rerio* (**D**), *Cyprinus carpio var. koi* (**E**), *Cyprinus carpio var. Songpu* (**F**), and *Sinocyclocheilus maitianheensis* (**G**).

Additional Files

Supplementary Table S1. Statistics of MGI reads for the *k*-mer analysis.

Supplementary Table S2. Genome size estimation of black and golden koi carps by using a *k*-mer analysis.

Supplementary Table S3. Statistics of sequencing reads from the PacBio and ONT sequencing platforms.

Supplementary Table S4. Statistics of sequencing reads from the Hi-C libraries.

Supplementary Table S5. Statistics of the 50 chromosomes (Chr) for both koi variants.

Supplementary Table S6. Statistics of repeat sequences in the assembled genomes.

Supplementary Table S7. Statistics of predicted gene structures in the black koi carp genome.

Supplementary Table S8. Statistics of predicted gene structures in the golden koi carp genome.

Supplementary Table S9. Summary of functional annotations.

Supplementary Table S10. NCBI accession numbers of the six representative fish genomes for phylogenetic analysis.

Supplementary Table S11. Summary of the best-hit gene pairs between the predicted ancestor and seven representative Cypriniformes species (including two koi carps).

Abbreviations

BUSCO: Benchmarking Universal Single-Copy Orthologs; Gb: gigabyte bases; Hi-C: high-throughput chromatin conformation capture; Mb: million bases; Mya: million years ago; NCBI: National Center for Biotechnology Information; T2T: telomere-to-telomere; TMRCA: the most recent common ancestor; WGD: whole genome duplication.

Acknowledgements

This study was supported by National Key Research and Development Program of China (nos. 2023YFE0205100 and 2022YFE0139700).

Author Contributions

Qiong Shi and Chao Bian conceived this study; Chao Bian and Rujingwen Huan performed sample collection and data analyses; Chao Bian and Rujingwen Huan wrote the manuscript; Qiong Shi and Chao Bian revised the manuscript.

Data Availability

Genome assemblies of black and golden koi carps in this study have been deposited in the NCBI database (BioProject IDs: PRJNA1191445 and PRJNA1191556). All supporting data and materials are available in the GigaScience database, GigaDB. Results of gene structure annotation are available in the Figshare repository: 10.6084/m9.figshare.27950550.

Competing Interests

The authors do not have competing interests related to this work.

References

1. Protas ME and Patel NH. Evolution of coloration patterns. Annu Rev Cell Dev Biol. 2008;24:425-46. doi:10.1146/annurev.cellbio.24.110707.175302.
2. Tian X, Peng N-n, Ma X, Wu L-m, Shi X, Liu H-f, et al. microRNA-430b targets scavenger receptor class B member 1 (scarb1) and inhibits coloration and carotenoid synthesis in koi carp (Cyprinus carpio L.). Aquaculture. 2021;546:737334. doi:10.1016/j.aquaculture.2021.737334.
3. Luo MK, Shi XL, Guo J, Lin K, Zhu WB, Fu JJ, et al. Deep spatiotemporal transcriptome analysis provides new insights into early development of koi carp (Cyprinus carpio var. koi). Aquaculture. 2023;575 doi:ARTN 739767
10.1016/j.aquaculture.2023.739767.
4. Rhoads A and Au KF. PacBio Sequencing and Its Applications. Genomics Proteomics Bioinformatics. 2015;13 5:278-89. doi:10.1016/j.gpb.2015.08.002.
5. Chen Y, Nie F, Xie SQ, Zheng YF, Dai Q, Bray T, et al. Efficient assembly of nanopore reads via highly accurate and intact error correction. Nat Commun. 2021;12 1:60. doi:10.1038/s41467-020-20236-7.
6. Marçais G and Kingsford C. A fast, lock-free approach for efficient parallel counting of occurrences of k-mers. Bioinformatics. 2011;27 6:764-70.
7. Ruan J and Li H. Fast and accurate long-read assembly with wtdbg2. Nat Methods. 2020;17 2:155-8. doi:10.1038/s41592-019-0669-3.
8. Langmead B and Salzberg SL. Fast gapped-read alignment with Bowtie 2. Nature methods. 2012;9 4:357.

9. Zhou C, McCarthy SA and Durbin R. YaHS: yet another Hi-C scaffolding tool. Bioinformatics. 2022;39 1 doi:10.1093/bioinformatics/btac808.
10. Durand NC, Shamim MS, Machol I, Rao SS, Huntley MH, Lander ES, et al. Juicer provides a one-click system for analyzing loop-resolution Hi-C experiments. Cell systems. 2016;3 1:95-8.
11. Dudchenko O, Batra SS, Omer AD, Nyquist SK, Hoeger M, Durand NC, et al. De novo assembly of the Aedes aegypti genome using Hi-C yields chromosome-length scaffolds. Science. 2017;356 6333:92-5.
12. Durand NC, Robinson JT, Shamim MS, Machol I, Mesirov JP, Lander ES, et al. Juicebox provides a visualization system for Hi-C contact maps with unlimited zoom. Cell systems. 2016;3 1:99-101.
13. Xu GC, Xu TJ, Zhu R, Zhang Y, Li SQ, Wang HW, et al. LR_Gapcloser: a tiling path-based gap closer that uses long reads to complete genome assembly. Gigascience. 2019;8 1 doi:10.1093/gigascience/giy157.
14. Xu M, Guo L, Gu S, Wang O, Zhang R, Peters BA, et al. TGS-GapCloser: A fast and accurate gap closer for large genomes with low coverage of error-prone long reads. Gigascience. 2020;9 9 doi:10.1093/gigascience/giaa094.
15. Lin Y, Ye C, Li X, Chen Q, Wu Y, Zhang F, et al. quarTeT: a telomere-to-telomere toolkit for gap-free genome assembly and centromeric repeat identification. Hortic Res. 2023;10 8:uhad127. doi:10.1093/hr/uhad127.
16. Waterhouse RM, Seppey M, Simao FA, Manni M, Ioannidis P, Klioutchnikov G, et al. BUSCO Applications from Quality Assessments to Gene Prediction and Phylogenomics. Mol Biol Evol. 2018;35 3:543-8. doi:10.1093/molbev/msx319.
17. Li H. Minimap2: pairwise alignment for nucleotide sequences. Bioinformatics. 2018;34 18:3094-100. doi:10.1093/bioinformatics/bty191.
18. Rhie A, Walenz BP, Koren S and Phillippy AM. Merqury: reference-free quality, completeness, and phasing assessment for genome assemblies. Genome Biology. 2020;21.
19. Abrusán G, Grundmann N, DeMester L and Makalowski W. TEclass—a tool for automated classification of unknown eukaryotic transposable elements. Bioinformatics. 2009;25 10:1329-30.
20. Xu Z and Wang H. LTR_FINDER: an efficient tool for the prediction of full-length LTR retrotransposons. Nucleic acids research. 2007;35 suppl_2:W265-W8.
21. Tarailo - Graovac M and Chen N. Using RepeatMasker to identify repetitive elements in genomic sequences. Current protocols in bioinformatics. 2009;25 1:4.10. 1-4.. 4.
22. Jurka J, Kapitonov VV, Pavlicek A, Klonowski P, Kohany O and Walichiewicz J. Repbase Update, a database of eukaryotic repetitive elements. Cytogenetic and genome research. 2005;110 1-4:462-7.
23. Benson G. Tandem repeats finder: a program to analyze DNA sequences. Nucleic acids research. 1999;27 2:573-80.
24. Mount DW. Using the basic local alignment search tool (BLAST). Cold Spring Harbor Protocols. 2007;2007 7:pdb. top17.
25. Birney E, Clamp M and Durbin R. GeneWise and genomewise. Genome research. 2004;14 5:988-95.
26. Kim D, Paggi JM, Park C, Bennett C and Salzberg SL. Graph-based genome alignment and genotyping with HISAT2 and HISAT-genotype. Nat Biotechnol. 2019;37 8:907-15.

doi:10.1038/s41587-019-0201-4.
27. Pollier J, Rombauts S and Goossens A. Analysis of RNA-Seq data with TopHat and Cufflinks for genome-wide expression analysis of jasmonate-treated plants and plant cultures. Methods Mol Biol. 2013;1011:305-15. doi:10.1007/978-1-62703-414-2_24.
28. Cantarel BL, Korf I, Robb SM, Parra G, Ross E, Moore B, et al. MAKER: an easy-to-use annotation pipeline designed for emerging model organism genomes. Genome research. 2008;18 1:188-96.
29. Boeckmann B, Bairoch A, Apweiler R, Blatter M-C, Estreicher A, Gasteiger E, et al. The SWISS-PROT protein knowledgebase and its supplement TrEMBL in 2003. Nucleic acids research. 2003;31 1:365-70.
30. Kulikova T, Aldebert P, Althorpe N, Baker W, Bates K, Browne P, et al. The EMBL nucleotide sequence database. Nucleic Acids Research. 2004;32 suppl_1:D27-D30.
31. Ogata H, Goto S, Sato K, Fujibuchi W, Bono H and Kanehisa M. KEGG: Kyoto encyclopedia of genes and genomes. Nucleic acids research. 1999;27 1:29-34.
32. Ashburner M, Ball CA, Blake JA, Botstein D, Butler H, Cherry JM, et al. Gene ontology: tool for the unification of biology. The Gene Ontology Consortium. Nat Genet. 2000;25 1:25-9. doi:10.1038/75556.
33. Hunter S, Apweiler R, Attwood TK, Bairoch A, Bateman A, Binns D, et al. InterPro: the integrative protein signature database. Nucleic acids research. 2009;37 suppl_1:D211-D5.
34. McGinnis S and Madden TL. BLAST: at the core of a powerful and diverse set of sequence analysis tools. Nucleic acids research. 2004;32 Web Server issue:W20-5. doi:10.1093/nar/gkh435.
35. Fischer S, Brunk BP, Chen F, Gao X, Harb OS, Iodice JB, et al. Using OrthoMCL to assign proteins to OrthoMCL-DB groups or to cluster proteomes into new ortholog groups. Current protocols in bioinformatics. 2011;Chapter 6:Unit 6.12.1-9. doi:10.1002/0471250953.bi0612s35.
36. Edgar RC. MUSCLE: multiple sequence alignment with high accuracy and high throughput. Nucleic Acids Res. 2004;32 5:1792-7. doi:10.1093/nar/gkh340.
37. Castresana J. Selection of conserved blocks from multiple alignments for their use in phylogenetic analysis. Molecular biology and evolution. 2000;17 4:540-52. doi:10.1093/oxfordjournals.molbev.a026334.
38. Yang Z. PAML 4: phylogenetic analysis by maximum likelihood. Mol Biol Evol. 2007;24 8:1586-91. doi:10.1093/molbev/msm088.
39. Lechner M, Findeiß S, Steiner L, Marz M, Stadler PF and Prohaska SJ. Proteinortho: Detection of (Co-)orthologs in large-scale analysis. BMC bioinformatics. 2011;12 1:124. doi:10.1186/1471-2105-12-124.
40. Kasahara M, Naruse K, Sasaki S, Nakatani Y, Qu W, Ahsan B, et al. The medaka draft genome and insights into vertebrate genome evolution. Nature. 2007;447 7145:714-9. doi:10.1038/nature05846.
41. Bian C, Hu Y, Ravi V, Kuznetsova IS, Shen X, Mu X, et al. The Asian arowana (Scleropages formosus) genome provides new insights into the evolution of an early lineage of teleosts. Sci Rep. 2016;6:24501. doi:10.1038/srep24501.
42. Lu Y, Li R, Xia L, Cheng J, Xia H, Zhan Q, et al. A chromosome-level genome assembly of the jade perch (Scortum barcoo). Sci Data. 2022;9 1:408. doi:10.1038/s41597-022-01523-y.

Figure 1

Click here to access/download;Figure;Figure 1.pdf

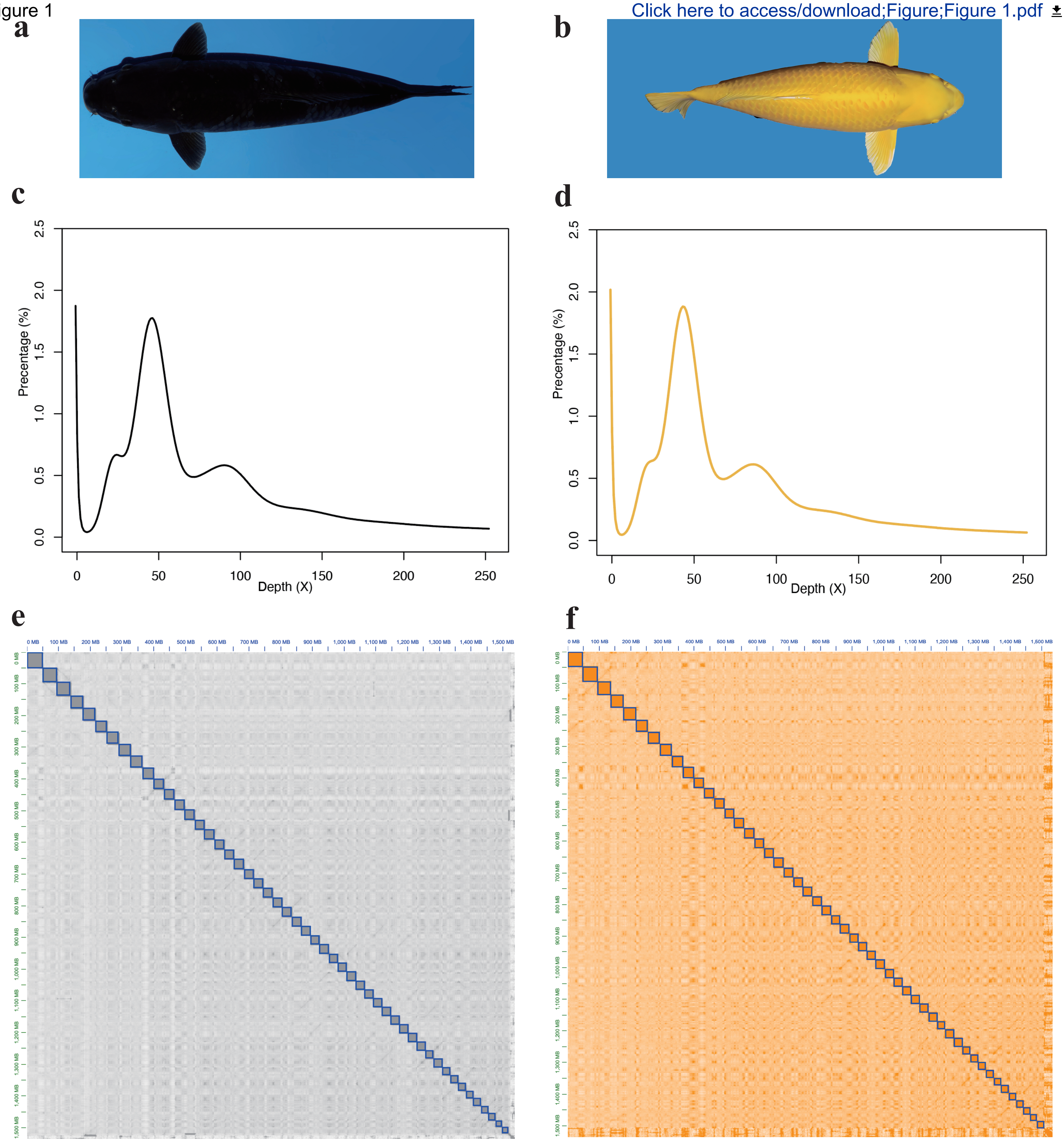

Figure 2

Click here to access/download;Figure;Figure 2.pdf

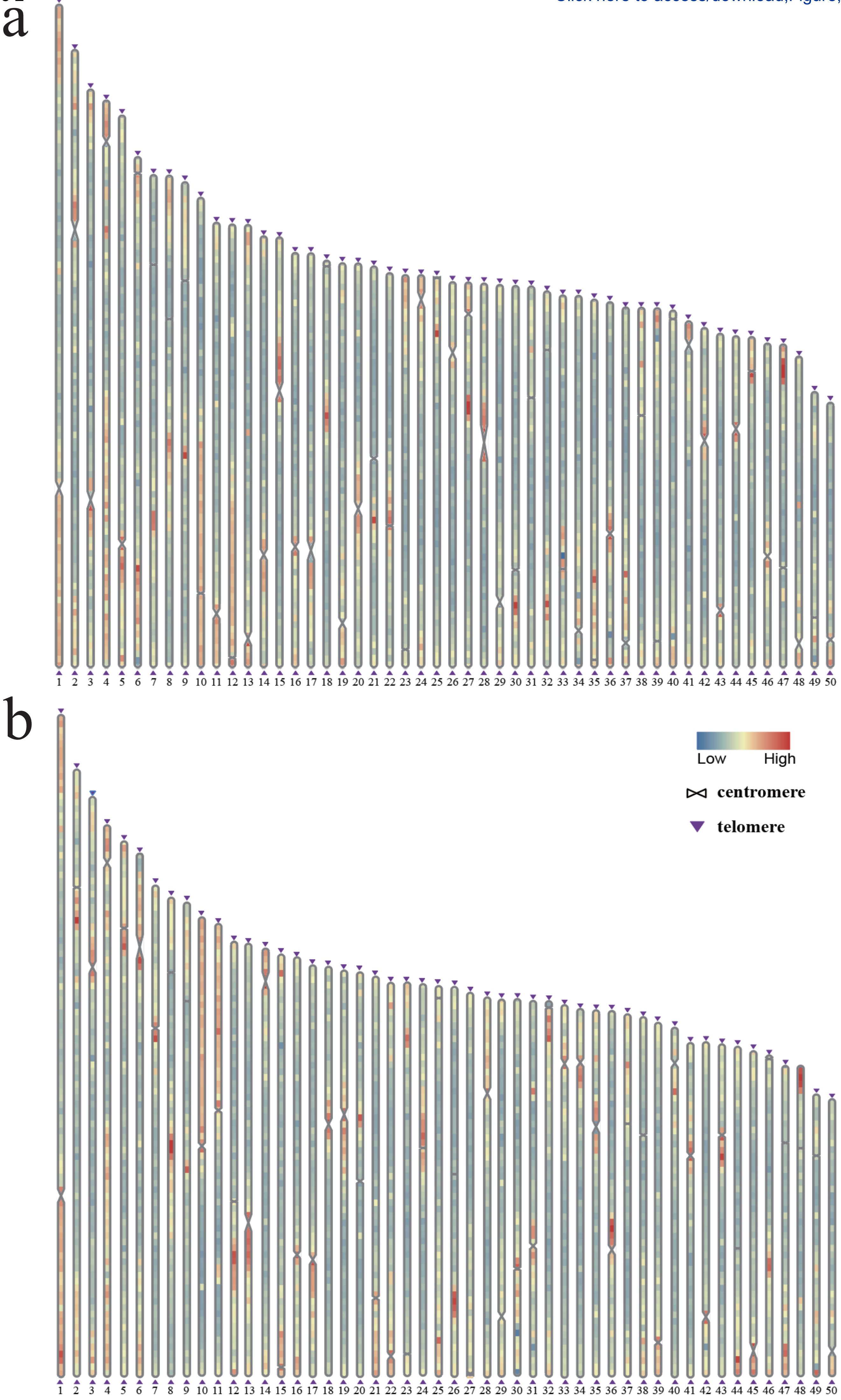

Figure 3

Click here to access/download;Figure;Figure 3.pdf

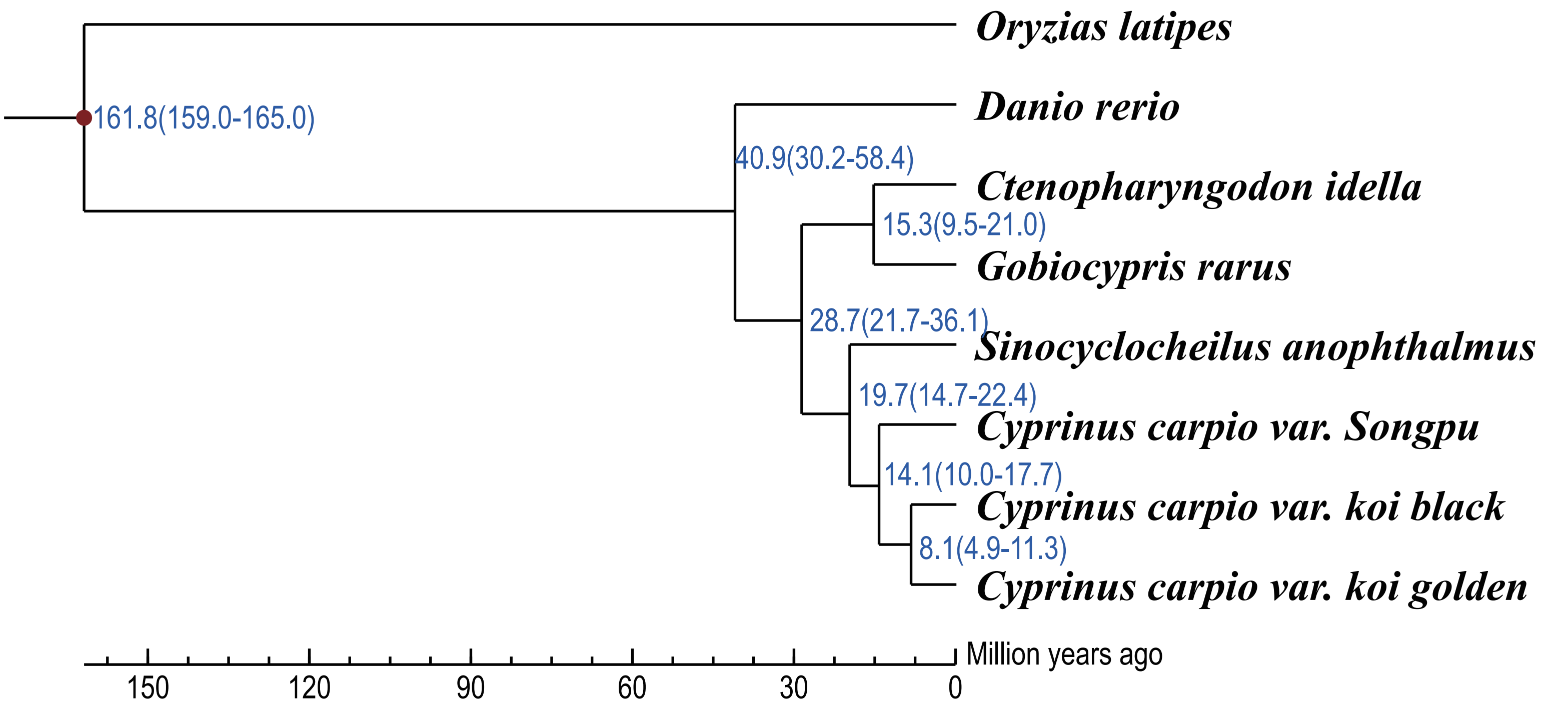

Figure 4

Click here to access/download;Figure;Figure 4.pdf

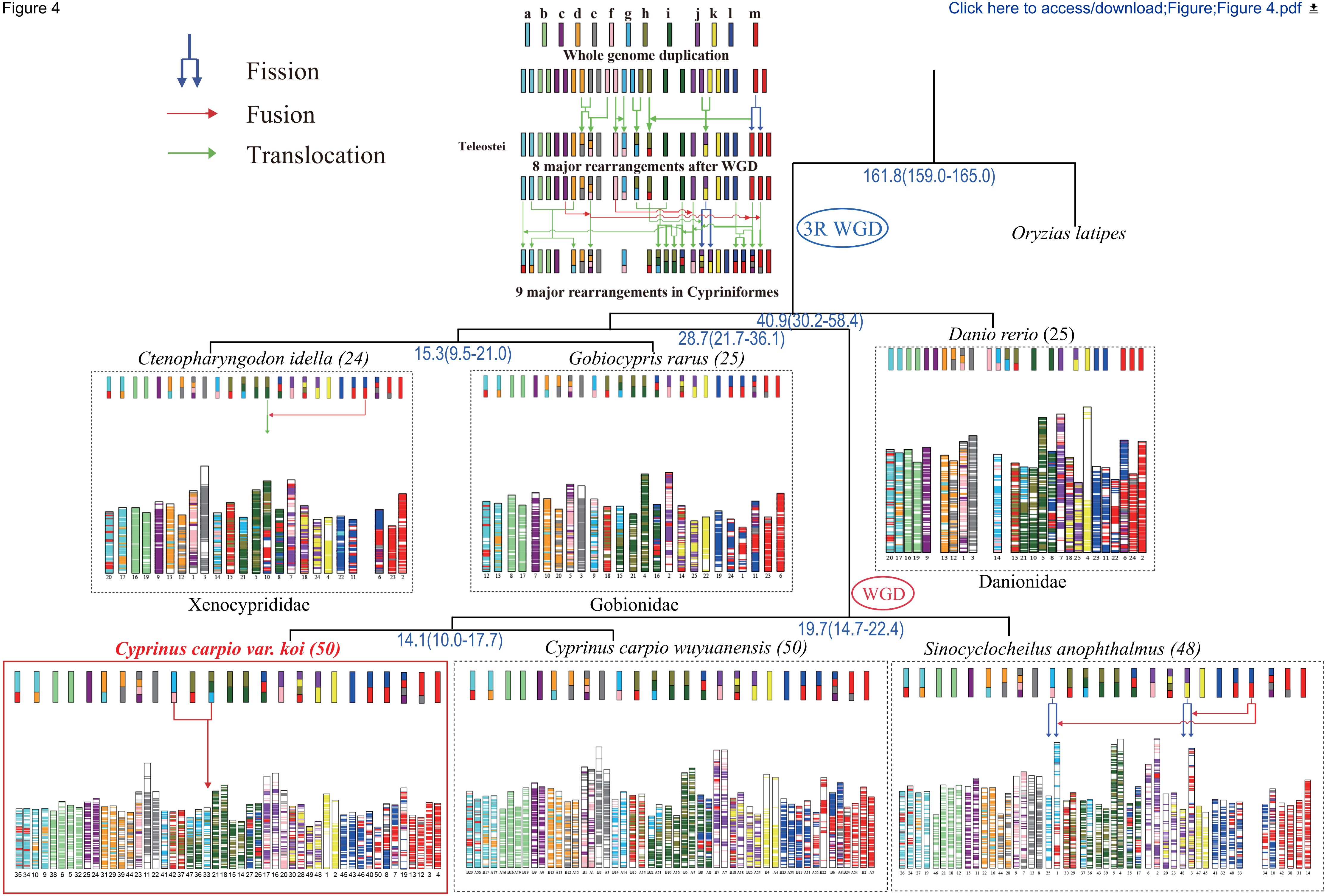

Click here to access/download
Supplementary Material
Supplementary materials 20241204.docx
