# Supplementary material for: Telomere-to-telomere chromosome-scale genome assemblies of black and golden koi carp variants support construction of an ancient karyotype of Cypriniformes
Source: Gigascience. 2025 Jul 26;14:giaf073. doi: 10.1093/gigascience/giaf073 (PMC13223729; doi:10.1093/gigascience/giaf073)
Supplement: giaf073_GIGA-D-24-00549_Revision_1 [file giaf073_giga-d-24-00549_revision_1.pdf]

GigaScience

Telomere-to-telomere chromosome-scale genome assemblies of black and golden koi carp variants support construction of an ancient karyotype of Cypriniformes

--Manuscript Draft--

**Manuscript Number:**	GIGA-D-24-00549R1
**Full Title:**	Telomere-to-telomere chromosome-scale genome assemblies of black and golden koi carp variants support construction of an ancient karyotype of Cypriniformes
**Article Type:**	Data Note
**Funding Information:**	Key Technologies Research and Development Program (2023YFE0205100) — professor Chao Bian
**Abstract:**	Background: Koi carp, a variant of the common carp, is one of the most popular ornamental fish. Its genomic resources can help us better understand chromosome evolution and color phenotypes in cyprinid fish.<br>Results: We constructed telomere-to-telomere chromosome-level genome assemblies for two koi carp variants (black and golden) by integrating MGI, PacBio HiFi, ONT, and Hi-C sequencing technologies. Haplotypic genomes comprised 50 chromosomes with 100 and 99 telomeres, respectively, with BUSCO results showing at least 98.8% completeness. We annotated a total of 55,023 and 54,569 protein-coding genes for black and golden koi carps, respectively, with over 96% assigned functional roles. Repetitive sequences occupy an estimated 636 Mb (41%) of the genomes. With phylogenetic analysis, we predict the koi carp variants to have split 5.3 million years ago, and we constructed an ancient karyotype of 25 ancestral chromosomes to reveal nine major chromosomal rearrangements.<br>Conclusions: Our study offers genome assemblies capable of predicting an ancient karyotype of Cypriniformes, with genomic resources available for in-depth investigations into diverse skin coloration in koi and other cypriniforms.
**Corresponding Author:**	Chao Bian, ph.D<br>Shenzhen University<br>shenzhen, CHINA
**Corresponding Author Secondary Information:**	
**Corresponding Author's Institution:**	Shenzhen University
**Corresponding Author's Secondary Institution:**	
**First Author:**	Chao Bian, ph.D
**First Author Secondary Information:**	
**Order of Authors:**	Chao Bian, ph.D
	Rujingwen Huan
	Qiong Shi
**Order of Authors Secondary Information:**	
**Response to Reviewers:**	Dear editor and reviewers,<br>Thanks for your valuable comments. We have made careful revisions throughout our manuscript accordingly. Changes are highlighted in red in the revised manuscript. Point-by-point responses to your comments are provided as follows for your re-review.<br><br>Reviewer #1: In this paper, the authors conducted two telomere-to-telomere chromosome-level genome assemblies for black and golden koi carp variants of common carp (Cyprinus carpio) using PacBio HiFi, ONT, and Hi-C sequencing technologies. BUSCO results validated 99%. A total of approximately 55,000 genes were annotated. These data show similar results to previous reports. Based on phylogenetic analysis and divergence time estimation, they predicted that the two koi

Powered by Editorial Manager® and ProduXion Manager® from Aries Systems Corporation

carp split from each other about 8.1 million years ago. I think that the data are valuable for the researchers in this field and suitable for publication in this journal after revisions.
Response: We highly appreciate the reviewer’s positive comments.

Several essential concerns should be addressed.
1. In the section introduction the authors mention as follows; "However, many important issues, such as how the ancestral chromosomes of teleost fishes evolved into those of Cypriniformes, and what changes occurred after the whole-genome duplication of ancestral chromosomes of Cypriniformes, remain largely unknown at present."
The changes after the whole-genome duplication of ancestral chromosomes of Cypriniformes were relatively well studied. The authors completely ignore previous genome reports for common carp. They should cite at least three references and discuss their differences and similarities.
Peng Xu et al., Nature Genetics volume 46, pages1212-1219 (2014)
Peng Xu et al., Nature Communications volume 10, Article number: 4625 (2019)
Jiong-Tang Li et al., Nature Genetics volume 53, pages1493-1503 (2021)
Response: Thanks for your nice advice. We removed these sentences “However, many issues, ...”, and rewrote the entire Introduction according to your suggestions. We also added all references you suggested, also more refs we found at the same time, we think it can cover the historical common carp genomes now. We also added some discussion about these researches in lines 42-52.
These references are cited in our revised manuscript, see following:
1.Xu P, Zhang X, Wang X, Li J, Liu G, Kuang Y, et al. Genome sequence and genetic diversity of the common carp, Cyprinus carpio. Nature Genetics. 2014;46 11:1212-9. doi:10.1038/ng.3098.
2.Ma W, Zhu ZH, Bi XY, et al. Allopolyploidization is Not So Simple: Evidence from the Origin of the Tribe Cyprinini (Teleostei: Cypriniformes). Current molecular medicine. 2014;14 doi:10.2174/1566524014666141203101543.
3.Xu P, Xu J, Liu GJ, et al. The allotetraploid origin and asymmetrical genome evolution of the common carp. Nature Communications. 2019;10 doi:ARTN 462510.1038/s41467-019-12644-1.
4.Chen L, Li CY, Li BJ, et al. Evolutionary divergence of subgenomes in common carp provides insights into speciation and allopolyploid success. Fund Res-China. 2024;4 3:589-602. doi:10.1016/j.fmre.2023.06.011.
5.Kon T, Omori Y, Fukuta K, et al. The Genetic Basis of Morphological Diversity in Domesticated Goldfish. Current Biology. 2020;30 12:2260-+. doi:10.1016/j.cub.2020.04.034.
6.Chen ZL, Omori Y, Koren S, et al. De novo assembly of the goldfish genome and the evolution of genes after whole-genome duplication. Science Advances. 2019;5 6 doi:ARTN eaav054710.1126/sciadv.aav0547.
7.Li JT, Wang Q, Yang MD, et al. Parallel subgenome structure and divergent expression evolution of allo-tetraploid common carp and goldfish. Nature Genetics. 2021;53 10:1493-+. doi:10.1038/s41588-021-00933-9.
8.Ren L, Gao X, Cui JL, et al. Symmetric subgenomes and balanced homoeolog expression stabilize the establishment of allopolyploidy in cyprinid fish. Bmc Biol. 2022;20 1 doi:ARTN 20010.1186/s12915-022-01401-4.
9.Luo J, Chai J, Wen YL, et al. From asymmetrical to balanced genomic diversification during rediploidization: Subgenomic evolution in allotetraploid fish. Science Advances. 2020;6 22 doi:ARTN eaaz767710.1126/sciadv.aaz7677.

2. The authors should refer to the following at least three papers that investigated allotetraploid in goldfish, a species very closely related to common carp, and discuss them.
Zelin Chen et al., Science Advances 2019 Vol 5, Issue 6 DOI: 10.1126/sciadv.aav0547
Tetsuo Kon et al., Current Biology 2020 doi.org/10.1016/j.cub.2020.04.034
Jing Luo et al., Science Advances 2020 Vol 6, Issue 22 DOI: 10.1126/sciadv.aaz7677
Response: Thanks for your constructive comment. Yes, we have added these references in the revised manuscript. We also applied the divergence time of 11-13.75 Mya for Cyprinus carpio and Sinocyclocheilus species from the Jing Luo’s study (Science Advances, 2020).
Reference in manuscript:
9.Luo J, Chai J, Wen YL, et al. From asymmetrical to balanced genomic diversification during rediploidization: Subgenomic evolution in allotetraploid fish. Science Advances.

2020;6 22 doi:ARTN eaaz767710.1126/sciadv.aaz7677.

3. The authors simply order the chromosome numbers by size in Supplementary Table S5. This should be adjusted to fit the three previous references (shown in comment 1). The authors should create a new supplementary table comparing the previously reported chromosomes with the chromosomes summarized in this paper.
Response: Many thanks for your good instructions. We added corresponding comparisons among black & golden koi and other two common carp references (Supplementary Table 6). We then obtained the one-to-one best-hit chromosomes between black & golden koi and C. carpio from Jiong-Tang Li's study (Nature Genetics, 2021), and reordered the chromosome numbers to be A and B subgenomes according to the references (Supplementary Tables 5).
Please see related changes in Supplementary Tables 5 and 6.

4. I reviewed the FASTA and GFF files. I recommend that you include gene symbols in the GFF file, otherwise it will be difficult to test the integrity of this reference sequence.
Response: Many thanks for your nice advice. Yes, we added gene symbols in the GFF files for both koi carps, and re-uploaded these data into the GigaScience FTP. The new gff files are named black.final.symbol.gff.gz and yellow.final.symbols.gff.gz.
For example:
scaffold_1 GeneWise mRNA 972449 1048204 62.65 + .
ID=2862at7898-D42;Shift=0; gene_symbol=ZN208;

5. The authors mention that "Based on a phylogenetic analysis and divergence time estimation, we predicted that the two koi carps split from each other about 8.1 million years ago." This is unlikely. Based on the results of six other papers (comments 1 and 2), these two variants appear to be much closer. Please review these six papers and recalculate your results.
Response: Many thanks for your constructive comments. You are right. The divergence time of 8.1 million years between two koi carps could be over-estimated. We reconstructed a divergence tree by using the following calibrated records: 180.0–251.5 million years ago (Mya) for D. rerio and O. latipes from Timetree website (https://timetree.org/home), 11.1-22.8 Mya for Cyprinus carpio and Sinocyclocheilus species [1], and 11-13.75 Mya for Cyprinus carpio and Sinocyclocheilus species [2]. The new divergence time between the two koi variants are about 5.3 Mya. See related change in lines 238-240.
Reference:
1. Li R, Wang X, Bian C, Gao Z, Zhang Y, Jiang W, et al. Whole-Genome Sequencing of Sinocyclocheilus maitianheensis Reveals Phylogenetic Evolution and Immunological Variances in Various Sinocyclocheilus Fishes. Front Genet. 2021;12:736500. doi:10.3389/fgene.2021.736500.
2. Luo J, Chai J, Wen Y, Tao M, Lin G, Liu X, et al. From asymmetrical to balanced genomic diversification during rediploidization: Subgenomic evolution in allotetraploid fish. Sci Adv. 2020;6 22:eaaz7677. doi:10.1126/sciadv.aaz7677.

Reviewer #2: The research paper entitled 'Telomere-to-telomere chromosome-scale genome assemblies of black and golden koi carp variants support construction of an ancient karyotype of Cypriniformes' by Chao Bian and collaborators reports the high-quality construction of genome assemblies for two cyprinid species using the latest sequencing technologies.
However, my opinion is that the manuscript is not adequately written and at the end the usefulness of their research to the scientific community is not explicit.
The length of the paper is acceptable but I am confident that if you had added few sentences more to make your reasoning clearer it would be justified. However, I consider that in this form the paper needs serious restructure and rewriting and some important topics need to be addressed before resubmission.
In brief, the writing of the paper is median, the use of English is not adequate and some sentences are really hard to follow and extract the meaning. The GigaScience journal is not addressed solely to geneticists, evolutionary biologists or ichthyologists but to a much broader research audience which will surely get confused. At the discussion section, which is totally absent, you need to help more the readers to comprehend the information you provide on WGS and genome evolution in cyprinids. For example, the fact that we may also encounter hexaploid cyprinids etc?

	Most manuscript's parts need serious restructuring and you may have to follow the guidelines of the journal, i.e., introduction, M&M, Results and Discussion or a combined "Results & Discussion" section.<br>Response: We would like to thank you for taking the time to give us valuable comments. We have made careful revisions accordingly.<br>1)For the usefulness of our research to the scientific community, we added a few sentences to clarify this in Introduction lines 61-67.<br>2)For the paper structure, since we submitted our manuscript as a Data Note. GigaScience journal recommended a Data Note paper contains the following sections: Context, Methods, Data Validation and quality control, Re-use potential. More details are here: https://academic.oup.com/gigascience/pages/data_note. We also discussed with the editor of GigaScience journal and confirmed that the current structure is ok for publication. If you have further suggestions, please let us know, we will try our best to make it meet the journal's requirements.<br>3)Sorry for our poorly writing, now the manuscript has been edited by the language service from GigaScience Press, and the journal also has another round copy editing before publication, hope you are happy with it now. All changes are highlighted in the revised manuscript for your convenient reviewing.<br>4)For this question: "At the discussion section, which is totally absent, you need to help more the readers to comprehend the information you provide on WGS and genome evolution in cyprinids. For example, the fact that we may also encounter hexaploid cyprinids etc?" We added more paragragh in Conclusion line 268-273:<br>"The species from Cyprininae contain diploids (2n=2X=50 or 48), tetraploids (2n=4X=100), hexaploids (2n=6X≈150) [54] and higher polyploids (2n: 417–470) [55]. The koi carp in Cyprininae superfamily, Cyprinidae family, Cyprinoidei suborder is tetraploid (2n=4X=100). The chromosome-level genome assemblies of Cyprininae fish of different ploidies can largely help to study how the chromosome evolution in Cyprininae fish with diverse ploidies."<br><br>Mainly for the introduction section, it needs to contain the reasoning for the research conducted, past information and knowledge on the topic.<br>Response: Thanks for your nice advice. For the Introduction, we added 4 carp genome studies, such as the common carp (Cyprinus carpio) and goldfish (Carassius auratus), in lines 42-60.<br><br>Do the black and golden variants interbreed? If they split 8 Mya, can they be considered separate species? Obviously not since they still keep the "Cyprinus carpio var. koi" as a Linnean species name. The quantity of the data is huge but the discussion on the results is very poor and superficial with references stopping at line 205!<br>Response: Many thanks for your questions and constructive comments. Black and golden variants can interbreed. We are sorry for pointing out that the predicted divergence time between both koi variants, 8 Mya, could be over-estimated. In fact, we reconstructed a divergence tree by using the following calibrated records: 180.0–251.5 million years ago (Mya) for D. rerio and O. latipes from Timetree website (https://timetree.org/home), 11.1-22.8 Mya for Cyprinus carpio and Sinocyclocheilus species [1], and 11-13.75 Mya for Cyprinus carpio and Sinocyclocheilus species [2]. The new divergence time between the two kois was narrowed to about 5.3 Mya (see Figure 3).<br>For discussion, we focused on elevating quality of the first two T2T genome assemblies of koi carps in this study, filling the gap in the current lack of genome resources in koi carps, and laying a good reference for large-scale resequencing to examine body colors and comparative genomics of koi in the coming future. As a Data Note paper, we majorly exhibited the high quality of both genome assemblies in the result section, the discussion part is really weaker than a Research article, but we have added some more sentences in Conclusion in lines 268-298, hope these are helpful now.<br><br>Reference:<br>1. Li R, Wang X, Bian C, Gao Z, Zhang Y, Jiang W, et al. Whole-Genome Sequencing of Sinocyclocheilus maitianheensis Reveals Phylogenetic Evolution and Immunological Variances in Various Sinocyclocheilus Fishes. Front Genet. 2021;12:736500. doi:10.3389/fgene.2021.736500.<br>2. Luo J, Chai J, Wen Y, Tao M, Lin G, Liu X, et al. From asymmetrical to balanced

	genomic diversification during rediploidization: Subgenomic evolution in allotetraploid fish. Sci Adv. 2020;6 22:eaaz7677. doi:10.1126/sciadv.aaz7677.<br><br>Minor comments:<br>Lines 17-19: it needs a better background explanation. I cannot understand its "precious variability in skin color" neither the "excellent model to study chromosome changes after undergoing WGD".<br>Response: Thanks for your nice advice. We removed "precious variability in skin color" and rewritten this section. See more details in lines 23-25.<br>“Background: Koi carp, a variant of the common carp, is one of the most popular ornamental fish. Its genomic resources can help us better understand chromosome evolution and color phenotypes in cyprinid fish.”<br><br>Line 29: better rephrase "of their whole genomes"<br>Response: Yes, it is done (in line 32). We revised this sentence to be ‘Repetitive sequences occupy an estimated 636 Mb (41%) of the genomes.’.<br><br>Line 36: rephrase "which support to predict an ancient…"<br>Response: Yes, this sentence is rephrased in lines 36-37 as ‘Our study offers genome assemblies capable of predicting an ancient karyotype of Cypriniformes,......’.<br><br>Line 61: why "on the other hand"?<br>Response: It was removed in the revised manuscript.<br><br>Line 62: "allotetraploid species with two diploid progenitors that diverged about 23 million years ago (Mya) and later hybridized about 12.4 Mya". The [2] is not correct at all and you should find the right one.<br>Lines 67-80: This last paragraph at the introduction section needs very serious restructure without duplicating the information you provide also in the abstract and the next M&M sections!<br>Response: Many thanks for your good advice. We thoroughly re-wrote the Introduction section and removed the previously inaccurate conclusion. (see lines 42-67)<br><br>Line 31: delete "from each other", it's by default when they split.<br>Line 32: either "cypriniforms" or "cypriniform species"; see also lines 38, 262, 265, 268, 289, 295, 310, 352.<br>Line 35: "Current data high-quality data…"<br>Line 47: delete "as we know, the"<br>Line 48: better move "especially in ornamental species" after the "overall market value" to make sense.<br>Line 51: rephrase "this makes it"<br>Line 54: better mention that miR-430 is a microRNA which is not obvious to everybody.<br>Line 96: delete "and then"<br>Line 99: "libraries were prepared with an Oxford…"<br>Response: We apologize for these mistakes. They were corrected in our revised manuscript.<br><br>Line 87: better also cite next to the technologies employed and the type of data produced (short vs long reads) to help readers understand why you did all that.<br>Response: Many thanks for your good advice. We cited a Review paper in line 78, entitled ‘Advances in Whole Genome Sequencing: Methods, Tools, and Applications in Population Genomics’. This review comprehensively reported the development and iteration of genome sequencing technologies, and provided detailed comparison of three sequencing technologies of Sanger, short reads and long reads [4].<br>Reference: 4. Lu Y, Li M, Gao Z, Ma H, Chong Y, Hong J, et al. Advances in Whole Genome Sequencing: Methods, Tools, and Applications in Population Genomics. Int J Mol Sci. 2025;26 1 doi:10.3390/ijms26010372.<br><br>Line 104: "Hi-C Kit and DpnII enzyme" with the enzyme name written in italics.<br>Response: Yes, it is done (in line 91).<br><br>Line 124: delete "by' before "using" and correct it all along the document too, e.g., in lines 145, 188, 207<br>Response: Yes, these are all corrected.

Powered by Editorial Manager® and ProduXion Manager® from Aries Systems Corporation

Line 166: "...database and were aligned to our assembled…"
Line 183: change to lower case "idella"; see also line 277
Line 191: for PhyML, it's not the reference [38]
Response: These are all revised. See more details in lines 151, 149, 167, 260 and 175.

Line 194: for TimeTree, better used the respective reference (Kumar et al., 2017). It is also strange but when I also used the website and entered the two species to estimate the divergence time, it showed to me 224 Mya (180-251.5 Mya); can you please explain how you got the 140-170 Mya estimate?
Response: Sorry for the mistake. In fact, we reconstructed a divergence tree by using the following calibrated records: 180.0–251.5 million years ago (Mya) for D. rerio and O. latipes from Timetree website (https://timetree.org/home), 11.1-22.8 Mya for Cyprinus carpio and Sinocyclocheilus species [1], and 11-13.75 Mya for Cyprinus carpio and Sinocyclocheilus species [2]. The new divergence time between two kois are narrowed to about 5.3 Mya (see Figure 3).
Reference:
1. Li R, Wang X, Bian C, Gao Z, Zhang Y, Jiang W, et al. Whole-Genome Sequencing of Sinocyclocheilus maitianheensis Reveals Phylogenetic Evolution and Immunological Variances in Various Sinocyclocheilus Fishes. Front Genet. 2021;12:736500. doi:10.3389/fgene.2021.736500.
2. Luo J, Chai J, Wen Y, Tao M, Lin G, Liu X, et al. From asymmetrical to balanced genomic diversification during rediploidization: Subgenomic evolution in allotetraploid fish. Sci Adv. 2020;6 22:eaaz7677. doi:10.1126/sciadv.aaz7677.

Line 205: "teleosts" in plural; see also line 259
Line 208: better delete "those"
Line 225: I read for both species, N50 is 30.0 Mb in Table 1!
Line 226: delete "Meanwhile"; "we also obtained…"
Response: These are all revised. See more details in lines 186, 188, 191, 242, 321 and 208.

Lines 231-234: you better mention that you followed a synteny criterion for the chromosome numbering in both species, i.e., chromosome 1 in black is closer to chromosome 1 in the golden koi.
Response: Thanks for your nice advice. We added corresponding comparisons among black & golden koi and other two common carp references (Supplementary Table 6). We then obtained the one-to-one best-hit chromosomes between black & golden koi and C. carpio from Jiong-Tang Li's study (Nature Genetics, 2021), and reordered the chromosome numbers to be A and B subgenomes according to the references (Supplementary Tables 5).
Please see related changes in Supplementary Tables 5 and 6.

Lines 239-240: what does the Mercury quality value of 46.8 stand for?
Response: Sorry for not clarifying this clearly. The Merqury can estimate the base-level accuracy by comparing k-mers in the assemblies to those from the HiFi reads. Therefore, the Merqury quality value of 46.8 stands for a per-base accuracy rate for assembly to be 0.9999988. We have provided more information in line 222.
"The Merqury program indicated that the quality values of both assemblies were 46.8, equating to a per-base accuracy rate of 0.9999988."

Lines 244-245: in practice, for the amount of repetitive sequences you may better write "636.21 and 636.24 Mb" (see Table S6)
Response: Yes, it is done, thanks. See the corrections in line 227.
"We predicted that the black and golden koi carp genomes contain 636.21 and 636.24 Mb of repetitive sequences."

Line 270: Figure 4 in the manuscript text (lines 318-326) seems to be not the same with the one uploaded at the end of the pdf file: see for example Cyprinus carpio wuyuanensis et letters A to G missing!
Response: Sorry for our mistake. We submitted a corrected PDF file of Figure 4 in our revised manuscript.

	Line 280: rephrase "one paired ancestral chromosomes were fused into two other chromosomes from different origins"<br>Response: Yes, it is done. We revised this sentence as 'Two fusion events and two fission events were occurred in chromosomes of Sinocyclocheilus species' in lines 262-263.<br><br>Lines 284-295: the Conclusion part needs a serious restructure without duplicating the information provided so far in the abstract, the introduction, and the results sections.<br>Response: Many thanks for your nice advice. Yes, we re-wrote the Conclusion section in lines 268-304 of our revised manuscript.<br><br>Line 303: "both genome sequencings".<br>Supplementary Table 10: there is no such reference in the manuscript; in the last column, better use "Haploid Chromosome Number" as a header.<br>Response: Thanks for your detailed instructions. Yes, we corrected these issues in our revised manuscript. See more details in line 312 and the new Table S11.
**Additional Information:**	
**Question**	**Response**
Are you submitting this manuscript to a special series or article collection?	No
**Experimental design and statistics**<br><br>Full details of the experimental design and statistical methods used should be given in the Methods section, as detailed in our Minimum Standards Reporting Checklist. Information essential to interpreting the data presented should be made available in the figure legends.<br><br>Have you included all the information requested in your manuscript?	Yes
**Resources**<br><br>A description of all resources used, including antibodies, cell lines, animals and software tools, with enough information to allow them to be uniquely identified, should be included in the Methods section. Authors are strongly encouraged to cite Research Resource Identifiers (RRIDs) for antibodies, model organisms and tools, where possible.<br><br>Have you included the information requested as detailed in our Minimum Standards Reporting Checklist?	Yes

**Availability of data and materials**<br><br>All datasets and code on which the conclusions of the paper rely must be either included in your submission or deposited in publicly available repositories (where available and ethically appropriate), referencing such data using a unique identifier in the references and in the “Availability of Data and Materials” section of your manuscript.<br><br>Have you have met the above requirement as detailed in our Minimum Standards Reporting Checklist?	Yes
GigaScience has policies and guidelines in place for the use of generative AI-writing tools such as ChatGPT. If you have used such writing tools to assist with writing the manuscript this must be declared and cited in the text. Authors should not list AI-writing tools and other AI-assisted technologies as an author or co-author and should acknowledge that they are fully responsible for text generated or refined by AI-writing tools.<p><br><br>A summary of use (particularly in the introduction or among methods) needs to be included at the end of the paper, and the outputs should also be included as a supplementary file hosted in GigaDB or other open repositories. Please read our guidelines for more information. <p><br><br>By submitting to GigaScience, you are aware of the journal's AI-writing tools policy, and if you have declared use of such tools below, you have acknowledged this where appropriate in your manuscript and have made a summary of use and outputs available. <p><br><b>AI-assisted writing tools have been used in the preparation of this manuscript?	No

Manuscript Click here to access/download;Manuscript;Manuscript 20250530_fixDOI.docx

Telomere-to-telomere chromosome-scale genome assemblies of black and golden koi carp variants support construction of an ancient karyotype of Cypriniformes

Chao Bian[1,2,*], Rujingwen Huan[1], Qiong Shi[1,2,*]

[1]Laboratory of Aquatic Genomics, College of Life Sciences and Oceanography, Shenzhen University, Shenzhen, Guangdong 518057, China

[2]Shenzhen Key Lab of Marine Genomics, BGI Academy of Marine Sciences, BGI Marine, Shenzhen, Guangdong 518081, China

* Correspondence address: Qiong Shi, Bldg A6 Room 324, College of Life Sciences and Oceanography, Shenzhen University, Shenzhen, Guangdong 518057, China. Email: shiqiong@szu.edu.cn or shiqiong@genomics.cn;

Chao Bian, Bldg A6 Room 510, College of Life Sciences and Oceanography, Shenzhen University, Shenzhen, Guangdong 518057, China. Email: bianchao@szu.edu.cn

ORCID IDs:

Chao Bian, https://orcid.org/0000-0001-9904-721X

Rujingwen Huan, https://orcid.org/0009-0001-6713-7154

Qiong Shi, https://orcid.org/0000-0002-6358-976X

Abstract

Background: Koi carp, a variant of the common carp, is one of the most popular ornamental fish. Its genomic resources can help us better understand chromosome evolution and color phenotypes in cyprinid fish.

Results: We constructed telomere-to-telomere chromosome-level genome assemblies for two koi carp variants (black and golden) by integrating MGI, PacBio HiFi, ONT, and Hi-C sequencing technologies. Haplotypic genomes comprised 50 chromosomes with 100 and 99 telomeres, respectively, with BUSCO results showing at least 98.8%

completeness. We annotated a total of 55,023 and 54,569 protein-coding genes for black and golden koi carps, respectively, with over 96% assigned functional roles. Repetitive sequences occupy an estimated 636 Mb (41%) of the genomes. With phylogenetic analysis, we predict the koi carp variants to have split 5.3 million years ago, and we constructed an ancient karyotype of 25 ancestral chromosomes to reveal nine major chromosomal rearrangements.

Conclusions: Our study offers genome assemblies capable of predicting an ancient karyotype of Cypriniformes, with genomic resources available for in-depth investigations into diverse skin coloration in koi and other cypriniforms.

Key words: Genetics and genomics; Koi carp; Ancestral chromosome

Introduction

Cypriniforms exhibit distinctive evolutionary characteristics in ecological adaptability and phenotypic diversity, serving as excellent models for polyploid genome evolution. Cyprinid species such as the common carp (*Cyprinus carpio*) and goldfish (*Carassius auratus*) display genomic complexity, originating from a special allotetraploidization event [1, 2], which led to asymmetric evolution between the two subgenomes (A and B) [3-5]. This asymmetry manifests as significant differences in gene retention rates, expression patterns, and methylation levels [3-7]. Studies suggest that, during the rediploidization process, allotetraploid cyprinids employ mechanisms – including chromosomal rearrangements, transposon dynamics equilibrium, and coordinated cis-/trans-regulatory interactions – to maintain subgenome stability and balanced expression [7-9].

Beyond their special genomic structure, coloration in cyprinids holds critical ecological and economic significance. Koi carp (*Cyprinus carpio var. koi,* NCBI Taxonomy ID: 1,499,333), a member of the Cyprinidae family, is one of the most popular ornamental fish species due to its diverse coloration. Recent research on koi carp has revealed the role of microRNA in carotenoid metabolism and pigmentation regulation [10]. Transcriptomic analyses have further identified key genes (such as *pax3*, *pax7*, and *gch2*) as crucial regulators of pigmentation and neural crest

development during early embryogenesis [11].

In this study, we report the first two telomere-to-telomere (T2T) genome assemblies for black and golden koi carp variants. Through ancestral chromosome reconstruction for cypriniforms, we reveal that their karyotypes underwent nine major chromosomal rearrangements over the course of evolution. The genome assemblies of black and golden koi carp variants not only advance our understanding of cyprinid genomic evolution, but also are essential resources for deciphering the genetic basis of phenotypic diversification within this family.

Methods

Sample collection, DNA extraction, and whole genome sequencing

Two female koi carp variants (one black and one golden) were collected from a local aquaculture farm in Guangzhou, Guangdong Province, China. We pooled muscle samples separately from both koi carps, and extracted genomic DNA (gDNA) from both samples for whole genome sequencing using MGI to produce short reads, PacBio HiFi and ONT to produce long reads, and high-throughput chromatin conformation capture (Hi-C) technologies [12].

For gDNA sequencing, libraries with an insert size of 500 bp were constructed using a MGIEasy UDB Universal Library Prep Set, following manufacturer's instructions (MGI Tech Co. Ltd., China, RRID:SCR_017981). These libraries were subsequently sequenced on a DNBSEQ-T7 machine (MGI). The HiFi long-read libraries were prepared using a SMRTbell Express Template Prep Kit 2.0 (Pacific Biosciences, USA), following manufacturer protocols. HiFi long-read data were generated through sequencing on a PacBio Sequel II platform (Pacific Biosciences, RRID:SCR_017990) [13]. Next, CCS software (SMRT Link v9.0) was employed to generate consensus sequences [14]. Both ONT ultra-long libraries were built using an Oxford Nanopore SQK-ULK001 kit according to manufacturer instructions (Oxford Nanopore Technologies, UK), which were then sequenced on a PromethION flow cell (Oxford Nanopore Technologies, RRID:SCR_017987). The ONT reads were

corrected by NECAT v200221 software (RRID: SCR_025350) with default parameters [15]. Both Hi-C libraries were constructed using a GrandOmics Hi-C Kit and *DpnII* enzyme according to the manufacturer's standard protocol (GrandOmics, China). Sequencing was conducted on an Illumina NovaSeq platform (Illumina, USA; RRID: SCR_016387). All sequencing reads were integrated for genome assembling and chromosome anchoring.

Total RNA samples were prepared from muscle, skin, liver and heart tissues of the koi carps using a TRIZOL Kit (Invitrogen, USA) in accordance with the manufacturer's instructions. The integrity and quality of extracted RNA were evaluated using an Agilent 2100 Bioanalyzer (Agilent Technologies, USA; RRID: SCR_018043), and only those samples with an RNA Integrity Number (RIN) over 7.0 were selected for subsequent library preparation. Next, cDNA libraries were constructed using DNA nanoballs in accordance with the manufacturer's protocol for a DNBSEQ platform (MGI; RRID:SCR_017981). These libraries were sequenced on a MGISEQ-2000 platform (MGI) with a paired-end model (150 bp long).

Genome size prediction, genome assembling, telomere identification, and assembly quality evaluation

Genome sizes of the black and golden koi carps were estimated using a 17-mer frequency distribution analysis [16] of cleaned MGI data (with an insert size of 500 bp) and calculated according to the following equation: genome size=k-mer number/the expectation of k-mer depth.

Initial genome assemblies were completed using Hifiasm v0.19.8 (detailed parameters: -t 16 --n-hap 4 --hg-size 1520m; RRID: SCR_021069) with PacBio HiFi and ONT reads [17]. The Hi-C sequencing reads were aligned onto the above assembled contigs using Bowtie 2 (parameters:–very-sensitive -L 30–score-min L, −0.6, −0.2–end-to-end –reorder; RRID:SCR_016368) [18], and YaHs v1.0 (RRID: SCR_022965) [19] was employed with default parameters to compute the chromosomal linkage information, based on alignment results. These alignments were subsequently used with Juicer v1.5 (parameters: chr_num 30; RRID:SCR_017226)

[20] and 3D-DNA v170123 (parameters: -m haploid -r 2; RRID:SCR_017227) [21] to anchor contigs onto primary chromosomes. Juicebox v1.11.08 (RRID:SCR_021172) [22] was used to refine the assembly. The primary chromosome-level genome assemblies of black and golden koi carps contained seven and eight gaps, respectively. To achieve a gap-free and T2T level, we sequentially applied LR_GapCloser v1.0 (parameters: -t 35 -m 1000000 -v 10000; RRID:SCR_016194) [23] and TGS-GapCloser v1.0.1 (parameter:-min_match 2000; RRID:SCR_017633) [24] to fill gaps within both genome assemblies. Centromere and telomere sequences were identified using QuarTeT software (RRID:SCR_025258) [25].

For quality evaluation of both assemblies, a Benchmarking Universal Single-Copy Orthologs (BUSCO; RRID:SCR015008) [26] evaluation was performed to predict completeness. We mapped PacBio HiFi and ONT reads onto the genome assemblies using Minimap2 (RRID:SCR018550) [27] to conduct more assembling correction. Quality value (QV) was estimated using Merqury-20200430 software (RRID: SCR_022964) [28], with the recommended k-mer of 20.

Repeat element annotation

Repeat elements in both genomes were identified through combination of *de novo* and homology-based methods. For *de novo* prediction, RepeatModeler v1.0.8 (RRID:SCR_015027) [29] and LTR_Finder v1.0.6 (RRID:SCR_015247) [30] were applied to detect different types of repeat elements. Then we generated both new repeat libraries by integrating RepeatMasker v4.0.623 (RRID:SCR_012954) [31] and Repbase TE v21.01 (RRID:SCR_021169) [32]. Tandem repeats were detected using Tandem Repeats Finder (parameters: 2 7 7 80 10 50 2000 -d -h; RRID:SCR_022193) [33]. With the new repeat libraries, we employed RepeatProteinMask v4.0.623 [31] and RepeatMasker v4.0.623 (RRID:SCR_012954) [31] to identify repetitive sequences.

Gene prediction and functional annotation

To annotate protein-coding genes, we integrated homology-alignment and transcriptome data to generate non-redundant sets of protein-coding genes for both

black and golden koi carps. For homology-based annotation, protein sequences of five representative species – zebrafish (*Danio rerio*), medaka (*Oryzias latipes*), grass carp (*Ctenopharyngodon idella*), common carp (*Cyprinus carpio var. wuyuanensis*) and golden-line barbel fish (*Sinocyclocheilus anophthalmus*) – were downloaded from the NCBI (RRID:SCR_006472) database and aligned to our assembled genomes using TBLASTN (e-value 10^{-5}; RRID:SCR_011822) [34]. Based on TBLASTN alignments, GeneWise v2.2.0 (parameters: --blast_eval 1e-5 --align_rate 0.5 --extend_len 500; RRID:SCR_015054) [35] predicted gene structures. Transcriptome reads were mapped onto the genomic components using HISAT2 (RRID:SCR_015530) [36] and the transcriptome annotation sets generated using Cufflinks v2.2.1 (RRID:SCR_014597) [37].

MAKER (max_dna_len=300000, min_contig=500, pred_flank=500, AED_threshold=1, split_hit=30000, single_exon=1, single_length=250, tries=2; RRID:SCR_005309) [38] was then utilized to integrate gene sets generated from the two methods, to produce final, non-redundant protein-coding gene sets. Functional annotation was conducted by aligning results with five public databases: SwissProt (RRID:SCR_021164) [39], TrEMBL [40], KEGG (RRID:SCR_012773) [41], Gene Ontology (GO, RRID:SCR_002811) [42], and InterPro (RRID:SCR_005829) [43].

Gene families, phylogenetic tree construction, and divergence time estimation

Chromosome-level genomes of five representative species of Cypriniformes (*Danio rerio, Ctenopharyngodon idella, Gobiocypris rarus, Sinocyclocheilus anophthalmus, Cyprinus carpio var. wuyuanensis*, and *Oryzias latipes*) were downloaded from NCBI for phylogenetic and divergence time analyses. For the phylogenetic analysis, BLASTP (RRID:SCR_001010) [44] and OrthoMCL(RRID:SCR_007839) [45] were performed for protein sequence alignment and gene-family clustering. All single-copy orthologous genes were aligned using MUSCLE v3.8.31(RRID:SCR_011812) [46] for all examined genomes. Then, the Gblocks (RRID:SCR_015945) [47] program was used to obtain conservative multi-sequence alignments. Finally, we employed PhyML v4.9 (RRID:SCR014932) [48] to construct a phylogenetic tree using the maximum

likelihood method. Species divergence times were estimated using MCMCTREE in PAML v4.9 (RRID:SCR014932) [49]. We used a divergence time point from TimeTree data (RRID:SCR021162), at 140–170 million years ago (Mya) between *Oryzias latipes* and *Danio rerio*, to calibrate divergence times.

Construction of ancestral chromosomes

From the protein sequences of seven representative species, Proteinortho v6.0.36 (RRID:SCR_024177) [50] was employed to obtain a single-copy protein set which was then concatenated into a supergene. A phylogenetic tree was constructed as above. Several previous studies reconstructed the 13 pairs of ancestral chromosomes (a~m) of teleosts, concluding that eight major rearrangements occurred after the third round of whole-genome duplication (WGD) [51-53]. Thus, the most recent common ancestor of teleosts was putatively considered to harbor 24 ancestral chromosomes [51-53].

Protein datasets of each representative species of Cypriniformes were aligned to the protein set of the predicted ancestral teleosts using BLASTP (with an E-value threshold of 1e-10; RRID:SCR_001010). We filtered out matching regions with same color – less than 20 per chromosome. Finally, we predicted chromosome rearrangements, including chromosome fissions, fusions and translocations. We applied SVG in Perl programming language to visualize the predicted karyotype of the ancestor of Cypriniformes. Gene sequence fragments homologous to the ancestral chromosomes were marked with corresponding colors.

Results

Summary of the sequencing reads and both genome assemblies

Through the *k*-mer analysis of MGI data (78.6 and 73.7 Gb, respectively; **Supplementary Table S1**), we estimated the genome sizes of black and golden koi carp variants to be around 1.53 Gb (**Figure 1, Supplementary S2**). We also sequenced both koi genomes using PacBio Sequel II and ONT platforms, obtaining a total of 99.5 and 108.9 Gb of PacBio Sequel long reads, and 29.1 and 36.6 Gb of

ONT ultra-long reads, respectively (**Supplementary Tables S3**). The initial genomes of assembled black and golden koi carps were 1.57 Gb and 1.55 Gb in length, respectively (**Table 1**), with their contig N50 reaching 30.0 Mb. We also obtained 204.7 Gb and 215.6 Gb of Hi-C reads for black and golden koi carps, respectively (**Supplementary Tables S4**). Through Hi-C read mapping and contig anchoring, we constructed 50 chromosomes with seven to eight gaps in the assembled genomes. After gap closing, both final assemblies reached a high-quality chromosome level.

The total of 50 chromosome sequences for black and golden koi carps comprised up to 1.55 and 1.54 Gb, respectively (see the detailed chromosome lengths corresponding to each of the black and golden koi carps in **Supplementary Tables S5**), accounting for about 98.9% and 99.3% of assembled contigs. The black koi carp genome contained a total of 100 telomeres, and the golden koi carp genome, 99 (only one of its chr48_A10 was not identified; **Figure 2b**). Our BUSCO results showed 98.9% and 98.8% completeness (**Table 1**), respectively. Mapping ONT and HiFi reads onto both assembled genomes, the MGI reads showed high mapped rates (97.0% and 97.6%, respectively). The Merqury program indicated that the quality values of both assemblies were 46.8, equating to a per-base accuracy rate of 0.9999988. The above evaluations confirmed the high quality and completeness of our genome assemblies for black and golden koi carps.

Repeat elements and gene annotation data

Repeat annotations were performed using both *de novo* and homology-based methods. We predicted that the black and golden koi carp genomes contain 636.21 and 636.24 Mb of repetitive sequences, accounting for 40.6% and 40.9% of their genomes, respectively (**Supplementary Tables S7)**.

Gene prediction was carried out using an integrated approach of homology-based and transcriptome-based annotations. We annotated a total of 55,023 and 54,569 protein-coding genes, with an average mRNA length of 15.8 and 16.0 kb, respectively (**Supplementary Tables S8 and S9**). Among them, over 96.1% and 97.1% of predicted genes were assigned with at least one functional role from among five

databases (SwissProt, TrEMBL, KEGG, GO, and InterPro; **Supplementary Table S10**).

Predicted divergence times and ancestral chromosomes of Cypriniformes

We predicted the divergence time between *C. carpio var. wuyuanensis* and *C. carpio var. koi* to be approximately 9.2 Mya, and our two koi carp variants split from each other about 5.3 Mya (**Figure 3**). A total of 25 ancestral chromosomes were predicted with nine major chromosomal rearrangements in Cypriniformes after divergence from the teleosts' common ancestor – including two fusions, one fission, four chromosomal translocations, and two complex chromosomal rearrangements (see more details in **Figure 4A**). We also constructed a phylogenetic tree for six representative cypriniform species with different karyotypes (n = 24, 25, 48 and 50; **Supplementary Table S11)**, and analyzed detailed variances within these examined species (**Figure 4B–G**). For comparison, we summarized total numbers of the best-hit gene pairs between the predicted ancestor and seven representative cypriniforms, including two koi carps (**Supplementary Table S12**).

These chromosomal rearrangements were conserved in the examined cypriniforms with hyplotypic 25 or 50 chromosomes. Two diploid fishes with a total of 25 chromosomes, including rare minnow (*Gobiocypris rarus*; **Figure 4C**) and zebrafish (*Danio rerio*; **Figure 4D**), have similar chromosome structure to the ancestral Cypriniformes chromosomes. Two tetraploid fishes with a total of 50 chromosomes – both purse red carp (*Cyprinus carpio wuyuanensis*) and koi carp (*Cyprinus carpio var. koi*) – experienced an additional WGD event, but their karyotypes were mostly conserved (**Figure 4E–F**). Only a small chromosomal fusion was found in koi carp (**Figure 4E**). Some special chromosomal fusions occurred in fishes with 24 and 48 chromosomes; for instance, the diploid grass carp (*Ctenopharyngodon idella*; n=24) experienced one chromosome fusion (**Figure 4B**), while the tetraploid *Sinocyclocheilus* species (n=48) experienced two chromosome fusions (**Figure 4G**) after the *Sinocyclocheilus*- Lineage WGD. Two fusion events and two fission events were occurred in chromosomes of *Sinocyclocheilus* species (**Figure**

4G). We therefore suspect that the ancestors of diploid and tetraploid Cypriniformes were completely separated before independent fusion events.

Conclusion

The species from Cyprininae contain diploids (2n=2X=50 or 48), tetraploids (2n=4X=100), hexaploids (2n=6X≈150) [54] and higher polyploids (2n: 417–470) [55]. The koi carp in Cyprininae superfamily, Cyprinidae family, Cyprinoidei suborder is tetraploid (2n=4X=100). The chromosome-level genome assemblies of Cyprininae fish of different ploidies can largely help to study how the chromosome evolution in Cyprininae fish with diverse ploidies. Our study demonstrates the potential for advanced sequencing technologies, particularly third-generation long-read sequencing, to produce high-fidelity genome assemblies including centromeric regions and numerous telomeres. Compared with previously published common carp genomes, our investigation enhanced accuracy and robustness, yielding more comprehensive telomere-to-telomere representation. The total 50 chromosome sequences of black and golden koi carps are up to 1.55 and 1.54 Gb, respectively (see detailed length of subgenome chromosome in **Supplementary Tables S5**), accounting for about 98.9% and 99.3% of the assembled contigs. The contig N50 of both black and golden koi carp genome assemblies achieved 30.0 Mb, representing a dramatic increase over earlier assemblies (0.068 Mb [1] and 1.55 Mb [7], **Supplementary Table S6**). Notably, our assemblies are entirely gap-free and achieve complete coverage from one telomere to another, in contrast to earlier versions that still contained unresolved regions. To our knowledge, these represent the first T2T chromosome-level assemblies generated for the common carp. BUSCO analysis confirmed near-complete gene representation (~99%), exceeding that of previous research (**Supplementary Table S6**).

We successfully resolved nearly all telomeres of the koi genomes. The black koi genome contains a total of 100 telomeres; while the golden koi genome contains 99, with only one telomere (on chr48) unresolved. The ability to elucidate telomeric and

sub-telomeric regions across koi chromosomes can provide more complete genome assemblies. Through comprehensive genome annotation, we identified 55,023 and 54,569 protein-coding genes in black and golden koi, respectively. We further reconstructed 25 ancestral chromosomes for Cypriniformes and revealed nine major chromosomal rearrangements after their divergence from a common ancestor of teleosts.

Overall, these high-quality assemblies will facilitate further investigation into the molecular basis of pigmentation and morphological diversity in koi development. They serve as a comprehensive reference for future large-scale resequencing of koi with different body colors and for single-cell transcriptomic profiling of skin tissues. Moreover, we anticipate that these genome resources will inform future efforts in marker-assisted selection and precision breeding of ornamental carp.

Table 1. Statistical analysis of genome assemblies and annotation results for black and golden koi carps.

Parameter	Black variant	Golden variant
MGI reads (Gb)	78.6	73.7
HiFi reads (Gb)	99.5	108.9
ONT reads (Gb)	29.1	36.6
Hi-C reads (Gb)	204.7	215.6
Genome size (Mb)	1,568.0	1,553.7
Chromosome N50 (Mb)	30.0	30.0
Gap number	0	0
Telomere number	100	99
Merqury QV	46.8	46.8
BUSCO value	98.9%	98.8%
Repeat ratio	40.6	40.9
Gene number	55,023	54,569
Average gene length (bp)	15,771.5	16,005.4

Functional gene number	52,855	52,975

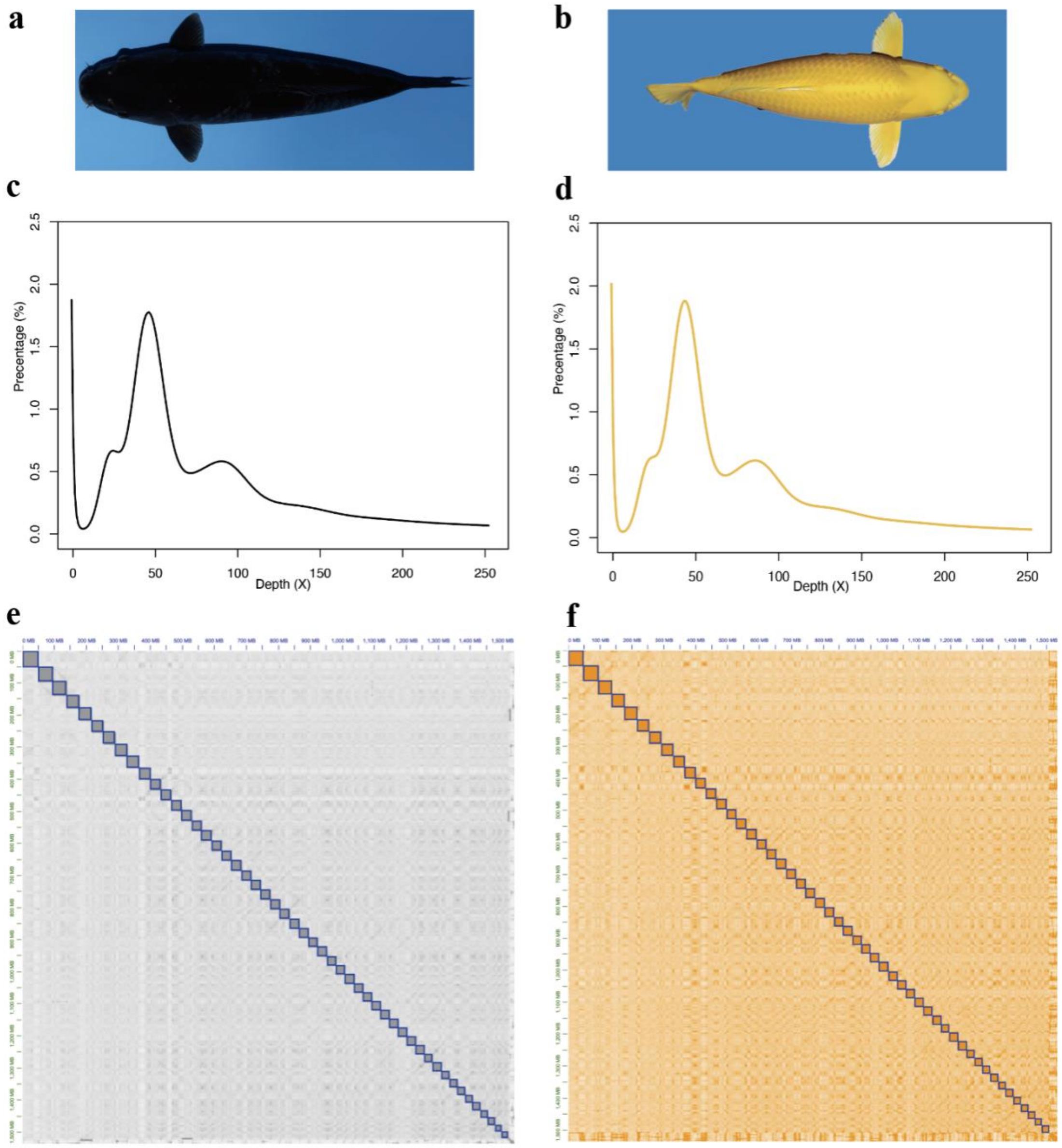

Figure 1. Black and golden koi carps subject to whole-genome sequencing. (**a-b**) images of the sequenced black and golden variants. (**c-d**) *k*-mer distribution for both genome sequences. (**e-f**) chromosome heatmaps of Hi-C data for black and golden koi carps, respectively.

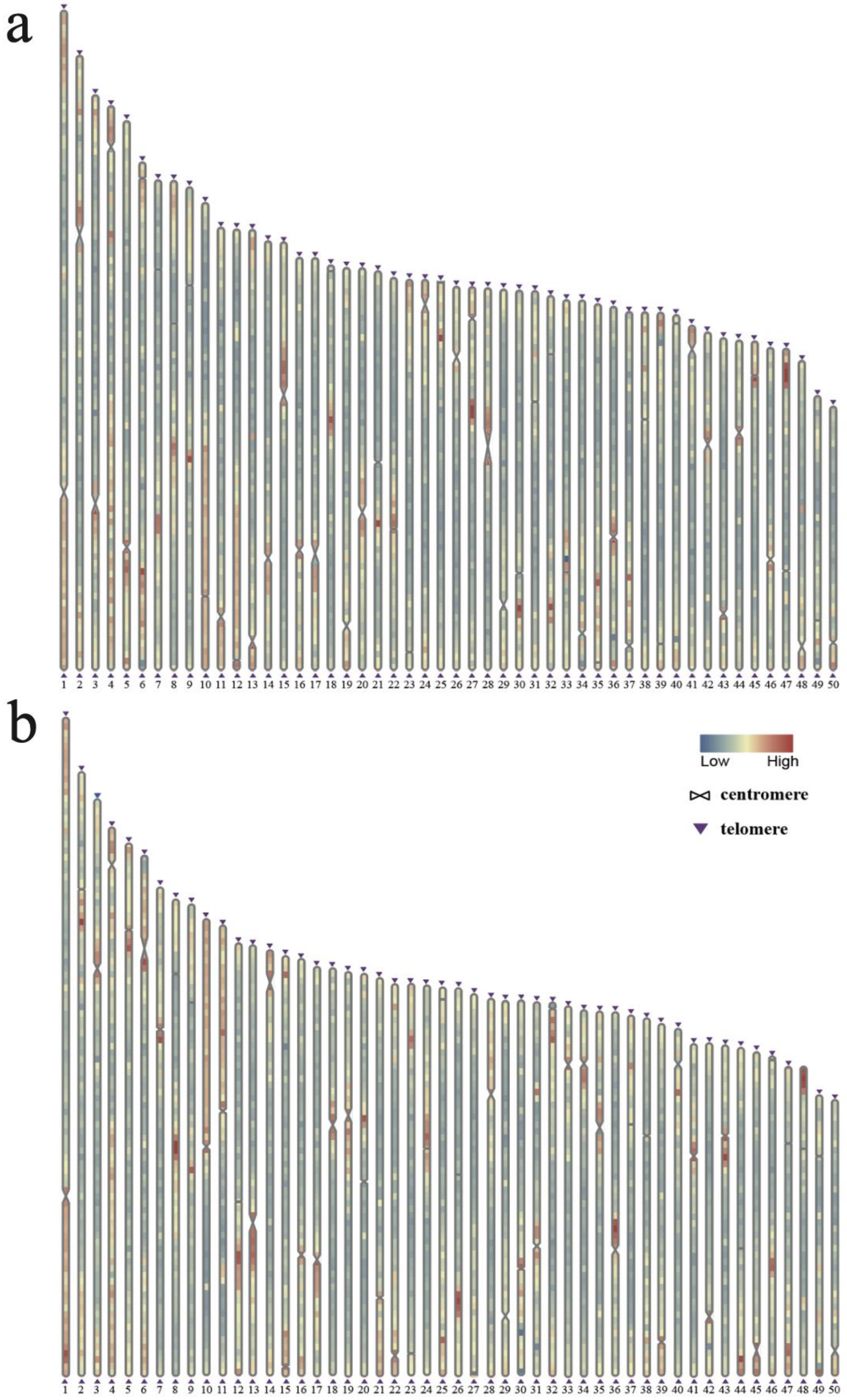

Figure 2. Genome-wide localization of centrosomes and telomeres. Details on each chromosome of black (**a**) and golden (**b**) koi carps for comparison.

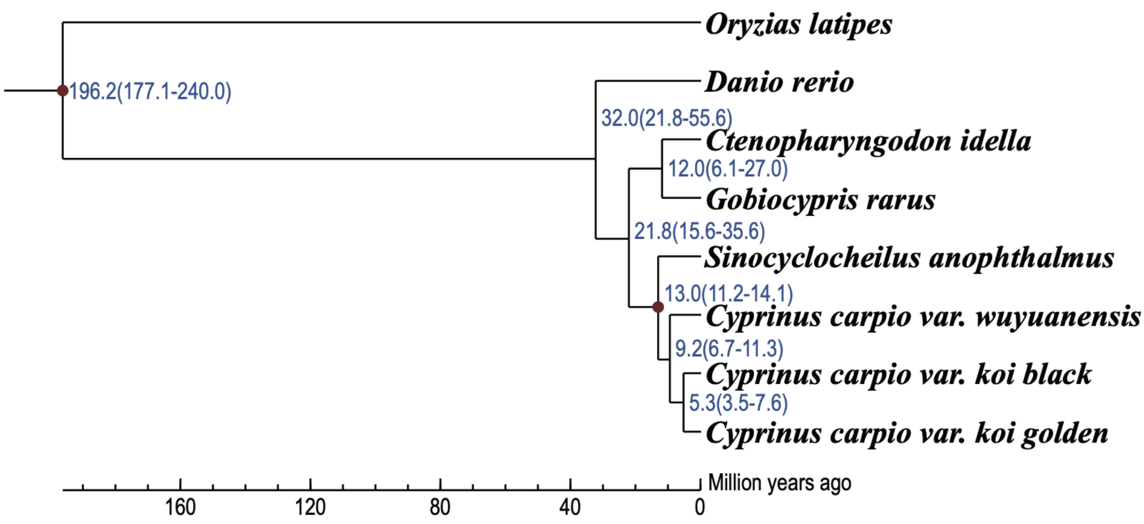

Figure 3. Divergence time tree of seven representative cypriniforms. Medaka (*Oryzias latipes*) was set as the outgroup. Blue numbers represent the estimated periods of divergence times. The red dot indicates the reference period for time divergence from TimeTree data (http://www.timetree.org/).

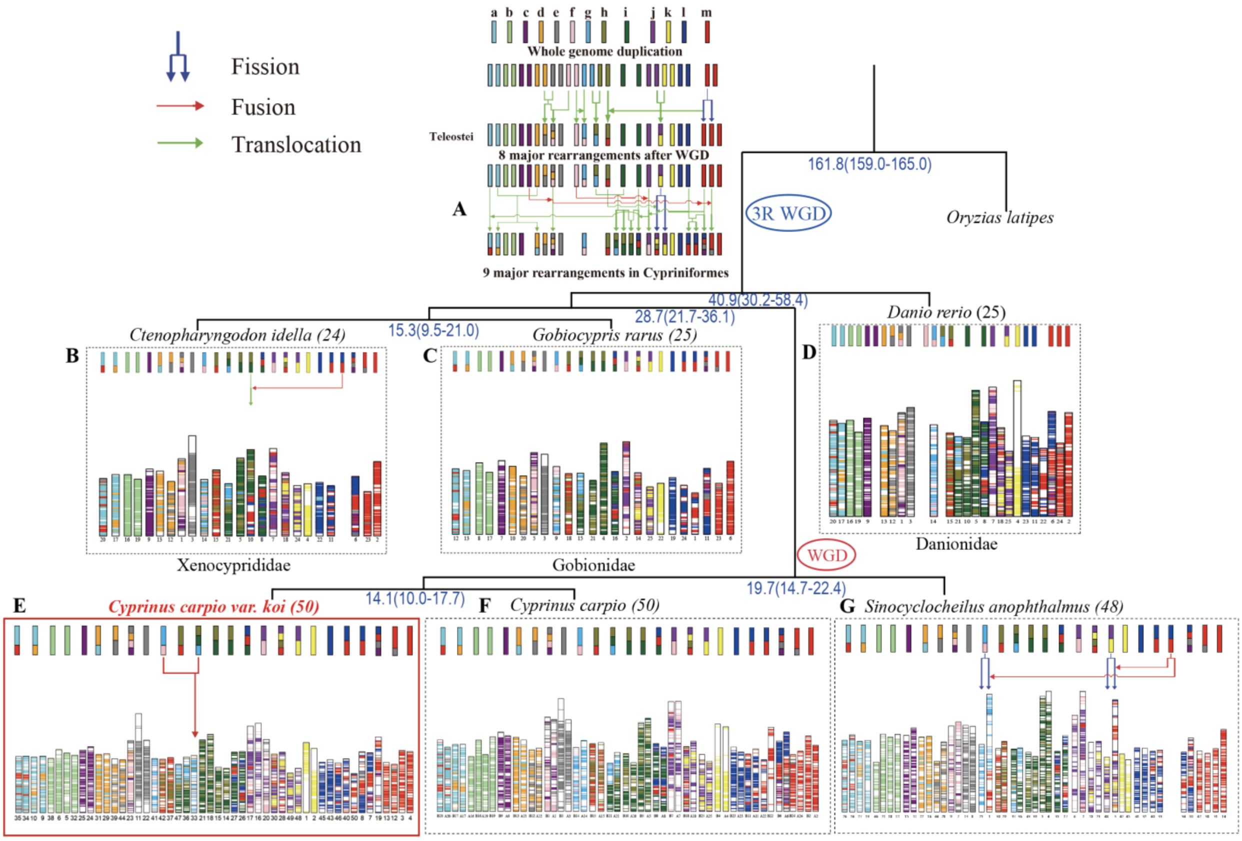

Figure 4. Evolution of chromosome karyotypes in Cypriniformes. Thirteen color bars represent teleost ancestor chromosomes a–m. Blue arrows indicate fission events, red arrows fusion, and green arrows, chromosomal translocation events. Red boxes mark new chromosomes after chromosomal fusions. Examined species include ancestors of Cypriniformes (**A**), *Ctenopharyngodon idella* (**B**), *Gobiocypris rarus* (**C**), *Danio rerio* (**D**), *Cyprinus carpio var. koi* (**E**), *Cyprinus carpio var. wuyuanensis* (**F**), and *Sinocyclocheilus maitianheensis* (**G**).

Additional Files

Supplementary Table S1. Statistical analysis of MGI reads for *k*-mer analysis.

Supplementary Table S2. Genome size estimation of black and golden koi carps using *k*-mer analysis.

Supplementary Table S3. Statistics of sequencing reads from PacBio and ONT sequencing platforms.

Supplementary Table S4. Statistical analysis of sequencing reads from Hi-C libraries.

Supplementary Table S5. Statistical analysis of the chromosome (Chr) details from available *C. carpio* genome assemblies.

Supplementary Table S6. Statistics of genome assemblies for two koi carps and two published common carps.

Supplementary Table S7. Statistical analysis of repeat sequences in the assembled genomes.

Supplementary Table S8. Statistics of predicted gene structures in the black koi carp genome.

Supplementary Table S9. Statistics of predicted gene structures in the golden koi carp genome.

Supplementary Table S10. Summary of functional annotations.

Supplementary Table S11. NCBI accession numbers of six representative fish genomes for phylogenetic analysis.

Supplementary Table S12. Summary of best-hit gene pairs between a predicted ancestor and seven representative cypriniforms, including two koi carps.

Abbreviations

BUSCO: Benchmarking Universal Single-Copy Orthologs; Gb: gigabyte bases; gDNA: genomic DNA; Hi-C: high-throughput chromatin conformation capture; Mb: million bases; Mya: million years ago; NCBI: National Center for Biotechnology Information; T2T: telomere-to-telomere; WGD: whole genome duplication.

Declarations

Ethics approval

The study protocol was approved by the Laboratory Animal Ethics Committee of Shenzhen University.

Competing Interests

The authors declare they have no competing interests.

Author Contributions

QS and CB conceived this study; CB and RH performed sample collection and data analyses; CB and RH wrote the manuscript; QS and CB revised the manuscript.

Funding

This study was supported by National Key Research and Development Program of China (no. 2023YFE0205100 and no. 2022YFE0139700) and Research Initiation Fund for Young Faculty Members at Shenzhen University (no. 000001032215).

Data Availability

Genome assemblies of black and golden koi carps in this study have been deposited in the NCBI database (BioProject IDs: PRJNA1191445 and PRJNA1191556). All additional supporting data are available in the GigaScience repository, GigaDB [56] with individual datasets for black koi [57] and golden koi [58].

References

1. Xu P, Zhang X, Wang X, Li J, Liu G, Kuang Y, et al. Genome sequence and genetic diversity of the common carp, *Cyprinus carpio*. Nature Genetics. 2014;46 11:1212-9. doi:10.1038/ng.3098.
2. Ma W, Zhu ZH, Bi XY, et al. Allopolyploidization is Not So Simple: Evidence from the Origin of the Tribe Cyprinini (Teleostei: Cypriniformes). Current molecular medicine. 2014;14 doi:10.2174/1566524014666141203101543.
3. Xu P, Xu J, Liu GJ, et al. The allotetraploid origin and asymmetrical genome evolution of the common carp. Nature Communications. 2019;10 doi:10.1038/s41467-019-12644-1.
4. Chen L, Li CY, Li BJ, et al. Evolutionary divergence of subgenomes in common carp provides insights into speciation and allopolyploid success. Fund Res-China. 2024;4 3:589-602.

doi:10.1016/j.fmre.2023.06.011.
5. Kon T, Omori Y, Fukuta K, et al. The Genetic Basis of Morphological Diversity in Domesticated Goldfish. Current Biology. 2020;30 12:2260-+. doi:10.1016/j.cub.2020.04.034.
6. Chen ZL, Omori Y, Koren S, et al. De novo assembly of the goldfish genome and the evolution of genes after whole-genome duplication. Science Advances. 2019;5 6 doi: 10.1126/sciadv.aav0547.
7. Li JT, Wang Q, Yang MD, et al. Parallel subgenome structure and divergent expression evolution of allo-tetraploid common carp and goldfish. Nature Genetics. 2021;53 10:1493-+. doi:10.1038/s41588-021-00933-9.
8. Ren L, Gao X, Cui JL, et al. Symmetric subgenomes and balanced homoeolog expression stabilize the establishment of allopolyploidy in cyprinid fish. Bmc Biol. 2022;20 1 doi: 10.1186/s12915-022-01401-4.
9. Luo J, Chai J, Wen YL, et al. From asymmetrical to balanced genomic diversification during rediploidization: Subgenomic evolution in allotetraploid fish. Science Advances. 2020;6 22 doi: 10.1126/sciadv.aaz7677.
10. Tian X, Peng N-n, Ma X, et al. microRNA-430b targets scavenger receptor class B member 1 (scarb1) and inhibits coloration and carotenoid synthesis in koi carp (Cyprinus carpio L.). Aquaculture. 2021;546:737334. doi:10.1016/j.aquaculture.2021.737334.
11. Luo MK, Shi XL, Guo J, et al. Deep spatiotemporal transcriptome analysis provides new insights into early development of koi carp (Cyprinus carpio var. koi). Aquaculture. 2023;575 doi:10.1016/j.aquaculture.2023.739767.
12. Lu Y, Li M, Gao Z, et al. Advances in Whole Genome Sequencing: Methods, Tools, and Applications in Population Genomics. Int J Mol Sci. 2025;26 1 doi:10.3390/ijms26010372.
13. Rhoads A and Au KF. PacBio Sequencing and Its Applications. Genomics Proteomics Bioinformatics. 2015;13 5:278-89. doi:10.1016/j.gpb.2015.08.002.
14. Chin CS, Alexander DH, Marks P, et al. Nonhybrid, finished microbial genome assemblies from long-read SMRT sequencing data. Nature Methods. 2013;10 6:563-+. doi:10.1038/Nmeth.2474.
15. Chen Y, Nie F, Xie SQ, et al. Efficient assembly of nanopore reads via highly accurate and intact error correction. Nat Commun. 2021;12 1:60. doi:10.1038/s41467-020-20236-7.
16. Marçais G and Kingsford C. A fast, lock-free approach for efficient parallel counting of occurrences of k-mers. Bioinformatics. 2011;27 6:764-70. doi:10.1093/bioinformatics/btr011.
17. Ruan J and Li H. Fast and accurate long-read assembly with wtdbg2. Nat Methods. 2020;17 2:155-8. doi:10.1038/s41592-019-0669-3.
18. Langmead B and Salzberg SL. Fast gapped-read alignment with Bowtie 2. Nature methods. 2012;9 4:357. doi:10.1038/nmeth.1923.
19. Zhou C, McCarthy SA and Durbin R. YaHS: yet another Hi-C scaffolding tool. Bioinformatics. 2022;39 1 doi:10.1093/bioinformatics/btac808.
20. Durand NC, Shamim MS, Machol I, et al. Juicer provides a one-click system for analyzing loop-resolution Hi-C experiments. Cell systems. 2016;3 1:95-8. doi:10.1016/j.cels.2016.07.002.
21. Dudchenko O, Batra SS, Omer AD, et al. De novo assembly of the Aedes aegypti genome using Hi-C yields chromosome-length scaffolds. Science. 2017;356 6333:92-5. doi:10.1126/science.aal3327.
22. Durand NC, Robinson JT, Shamim MS, et al. Juicebox provides a visualization system for Hi-C contact maps with unlimited zoom. Cell systems. 2016;3 1:99-101.

doi:10.1016/j.cels.2015.07.012.
23. Xu GC, Xu TJ, Zhu R, et al. LR_Gapcloser: a tiling path-based gap closer that uses long reads to complete genome assembly. Gigascience. 2019;8 1 doi:10.1093/gigascience/giy157.
24. Xu M, Guo L, Gu S, et al. TGS-GapCloser: A fast and accurate gap closer for large genomes with low coverage of error-prone long reads. Gigascience. 2020;9 9 doi:10.1093/gigascience/giaa094.
25. Lin Y, Ye C, Li X, et al. quarTeT: a telomere-to-telomere toolkit for gap-free genome assembly and centromeric repeat identification. Hortic Res. 2023;10 8:uhad127. doi:10.1093/hr/uhad127.
26. Waterhouse RM, Seppey M, Simao FA, et al. BUSCO Applications from Quality Assessments to Gene Prediction and Phylogenomics. Mol Biol Evol. 2018;35 3:543-8. doi:10.1093/molbev/msx319.
27. Li H. Minimap2: pairwise alignment for nucleotide sequences. Bioinformatics. 2018;34 18:3094-100. doi:10.1093/bioinformatics/bty191.
28. Rhie A, Walenz BP, Koren S, et al. Merqury: reference-free quality, completeness, and phasing assessment for genome assemblies. Genome Biology. 2020;21. doi:10.1186/s13059-020-02134-9.
29. Abrusán G, Grundmann N, DeMester L, et al. TEclass—a tool for automated classification of unknown eukaryotic transposable elements. Bioinformatics. 2009;25 10:1329-30. doi:10.1093/bioinformatics/btp084.
30. Xu Z and Wang H. LTR_FINDER: an efficient tool for the prediction of full-length LTR retrotransposons. Nucleic acids research. 2007;35 suppl_2:W265-W8. doi:10.1093/nar/gkm286.
31. Tarailo‐Graovac M and Chen N. Using RepeatMasker to identify repetitive elements in genomic sequences. Current protocols in bioinformatics. 2009;25 1:4.10. 1-4.. 4. doi:10.1002/0471250953.bi0410s25.
32. Jurka J, Kapitonov VV, Pavlicek A, et al. Repbase Update, a database of eukaryotic repetitive elements. Cytogenetic and genome research. 2005;110 1-4:462-7. doi:10.1159/000084979.
33. Benson G. Tandem repeats finder: a program to analyze DNA sequences. Nucleic acids research. 1999;27 2:573-80. doi:10.1093/nar/27.2.573.
34. Mount DW. Using the basic local alignment search tool (BLAST). Cold Spring Harbor Protocols. 2007;2007 7:pdb. top17. doi:10.1101/pdb.top17.
35. Birney E, Clamp M and Durbin R. GeneWise and genomewise. Genome research. 2004;14 5:988-95. doi:10.1101/gr.1865504.
36. Kim D, Paggi JM, Park C, et al. Graph-based genome alignment and genotyping with HISAT2 and HISAT-genotype. Nat Biotechnol. 2019;37 8:907-15. doi:10.1038/s41587-019-0201-4.
37. Pollier J, Rombauts S and Goossens A. Analysis of RNA-Seq data with TopHat and Cufflinks for genome-wide expression analysis of jasmonate-treated plants and plant cultures. Methods Mol Biol. 2013;1011:305-15. doi:10.1007/978-1-62703-414-2_24.
38. Cantarel BL, Korf I, Robb SM, et al. MAKER: an easy-to-use annotation pipeline designed for emerging model organism genomes. Genome research. 2008;18 1:188-96. doi:10.1101/gr.6743907.
39. Boeckmann B, Bairoch A, Apweiler R, et al. The SWISS-PROT protein knowledgebase and its supplement TrEMBL in 2003. Nucleic acids research. 2003;31 1:365-70. doi:10.1093/nar/gkg095.
40. Kulikova T, Aldebert P, Althorpe N, et al. The EMBL nucleotide sequence database. Nucleic

Acids Research. 2004;32 suppl_1:D27-D30. doi:10.1093/nar/gkh120.
41. Ogata H, Goto S, Sato K, et al. KEGG: Kyoto encyclopedia of genes and genomes. Nucleic acids research. 1999;27 1:29-34. doi:10.1093/nar/27.1.29.
42. Ashburner M, Ball CA, Blake JA, et al. Gene ontology: tool for the unification of biology. The Gene Ontology Consortium. Nat Genet. 2000;25 1:25-9. doi:10.1038/75556.
43. Hunter S, Apweiler R, Attwood TK, et al. InterPro: the integrative protein signature database. Nucleic acids research. 2009;37 suppl_1:D211-D5. doi:10.1093/nar/gkn785.
44. McGinnis S and Madden TL. BLAST: at the core of a powerful and diverse set of sequence analysis tools. Nucleic acids research. 2004;32 Web Server issue:W20-5. doi:10.1093/nar/gkh435.
45. Fischer S, Brunk BP, Chen F, et al. Using OrthoMCL to assign proteins to OrthoMCL-DB groups or to cluster proteomes into new ortholog groups. Current protocols in bioinformatics. 2011;Chapter 6:Unit 6.12.1-9. doi:10.1002/0471250953.bi0612s35.
46. Edgar RC. MUSCLE: multiple sequence alignment with high accuracy and high throughput. Nucleic Acids Res. 2004;32 5:1792-7. doi:10.1093/nar/gkh340.
47. Castresana J. Selection of conserved blocks from multiple alignments for their use in phylogenetic analysis. Molecular biology and evolution. 2000;17 4:540-52. doi:10.1093/oxfordjournals.molbev.a026334.
48. Guindon S, Dufayard JF, Lefort V, et al. New algorithms and methods to estimate maximum-likelihood phylogenies: assessing the performance of PhyML 3.0. Syst Biol. 2010;59 3:307-21. doi:10.1093/sysbio/syq010.
49. Yang Z. PAML 4: phylogenetic analysis by maximum likelihood. Mol Biol Evol. 2007;24 8:1586-91. doi:10.1093/molbev/msm088.
50. Lechner M, Findeiß S, Steiner L, et al. Proteinortho: Detection of (Co-)orthologs in large-scale analysis. BMC bioinformatics. 2011;12 1:124. doi:10.1186/1471-2105-12-124.
51. Kasahara M, Naruse K, Sasaki S, et al. The medaka draft genome and insights into vertebrate genome evolution. Nature. 2007;447 7145:714-9. doi:10.1038/nature05846.
52. Bian C, Hu Y, Ravi V, et al. The Asian arowana (Scleropages formosus) genome provides new insights into the evolution of an early lineage of teleosts. Sci Rep. 2016;6:24501. doi:10.1038/srep24501.
53. Lu Y, Li R, Xia L, et al. A chromosome-level genome assembly of the jade perch (Scortum barcoo). Sci Data. 2022;9 1:408. doi:10.1038/s41597-022-01523-y.
54. Gui J and Zhou L. Genetic basis and breeding application of clonal diversity and dual reproduction modes in polyploid Carassius auratus gibelio. Sci China Life Sci. 2010;53 4:409-15. doi:10.1007/s11427-010-0092-6.
55. Leggatt R and Iwama G. Occurrence of polyploidy in fishes. Reviews in Fish Biology and Fisheries. 2003;13:237-46. doi:10.1023/B:RFBF.0000033049.00668.fe.
56. Bian C; Huan R; Shi Q (2025): Supporting data for "Telomere-to-telomere chromosome-scale genome assemblies of black and golden koi carp variants support construction of an ancient karyotype of Cypriniformes" GigaScience Database. https://doi.org/10.5524/102715
57. Bian C; Huan R; Shi Q (2025): Chromosome-scale genome assembly of the black koi carp, Cyprinus carpio 'koi' GigaScience Database. https://doi.org/10.5524/102716
58. Bian C; Huan R; Shi Q (2025): Chromosome-scale genome assembly of the golden koi carp, Cyprinus carpio 'koi' GigaScience Database. https://doi.org/10.5524/102717

Figure 1

Click here to access/download;Figure;Figure 1.pdf

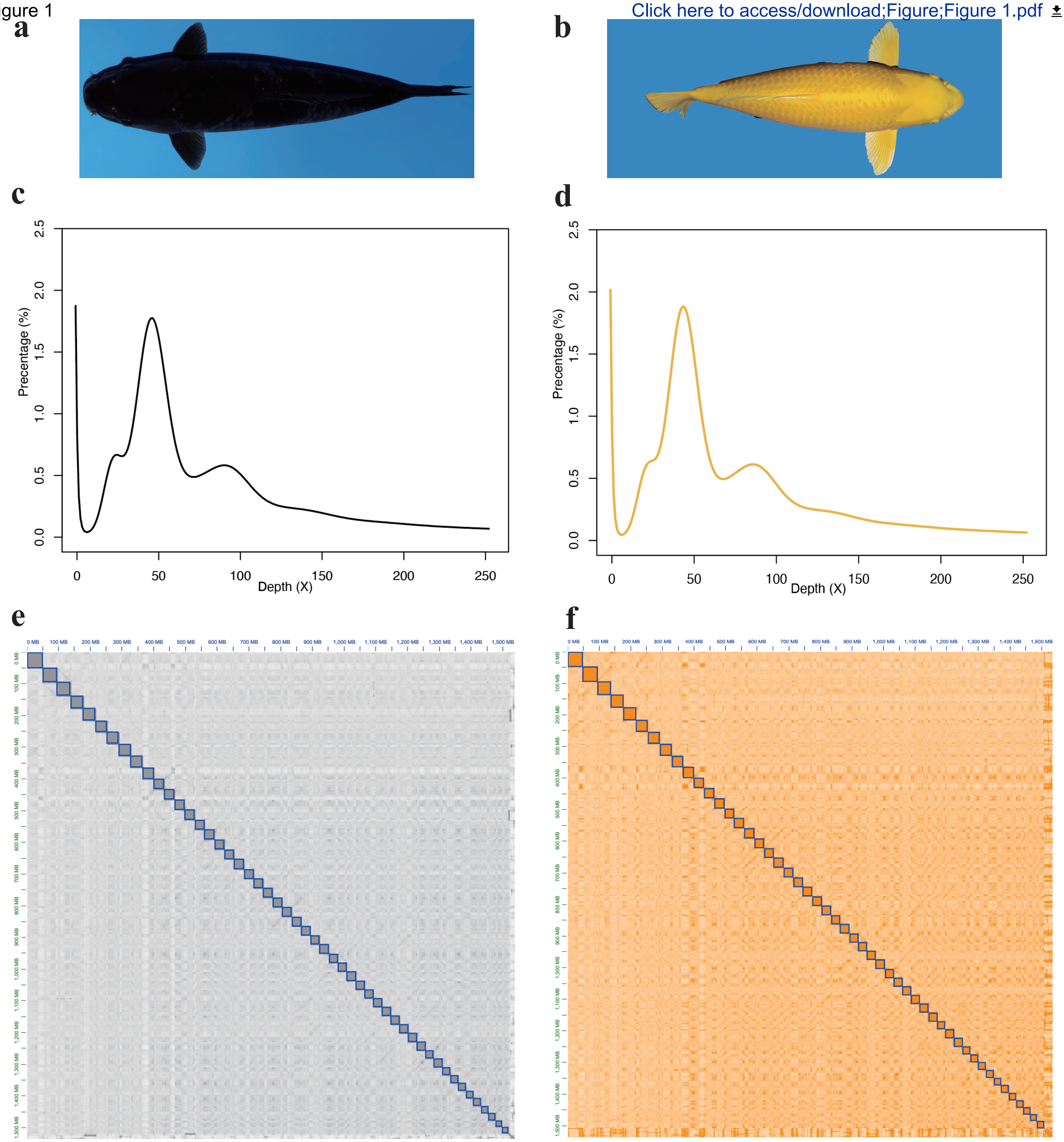

Figure 2

Click here to access/download;Figure;Figure 2.pdf

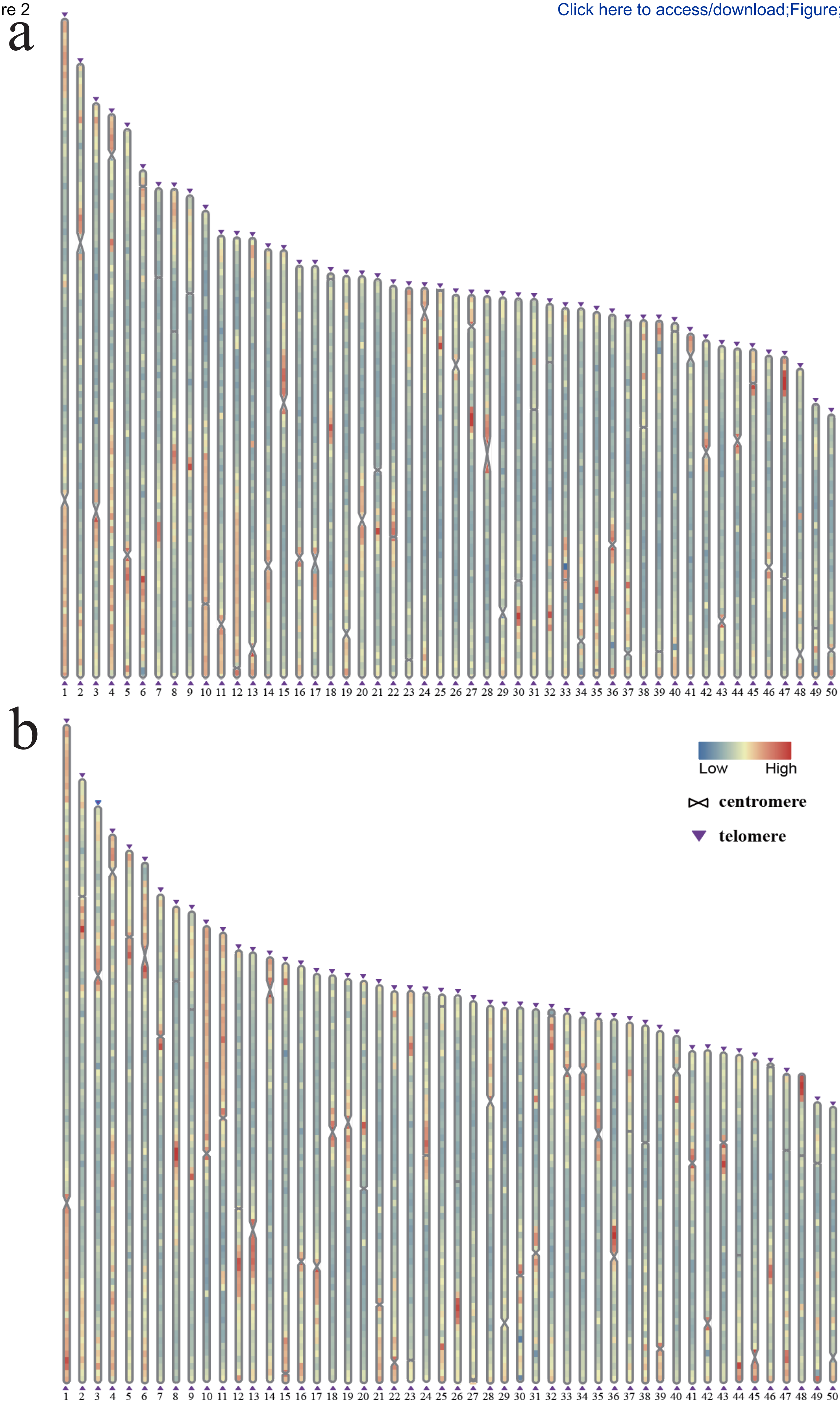

Figure 3

Click here to access/download;Figure;Figure 3new.pdf

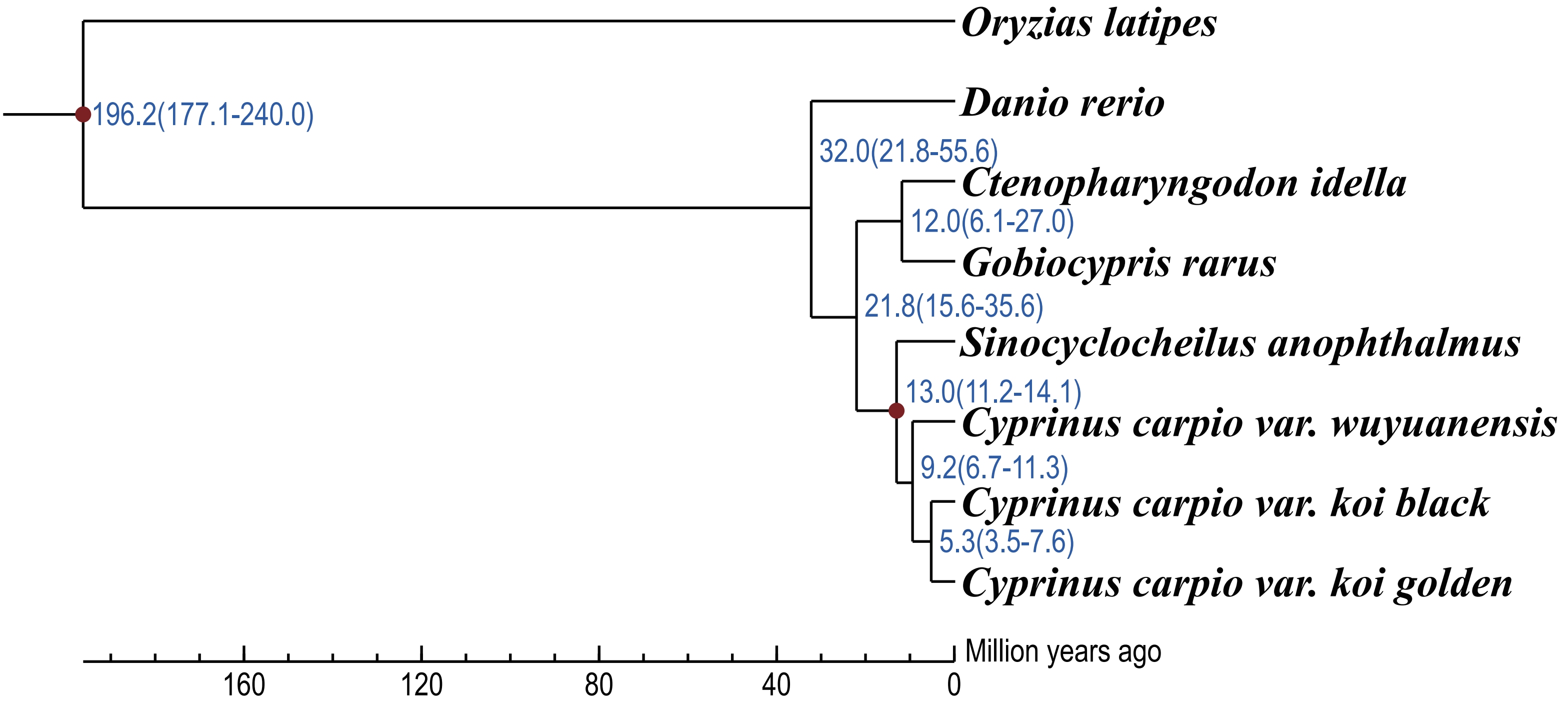

Figure 4

Click here to access/download;Figure;Figure 4new.pdf

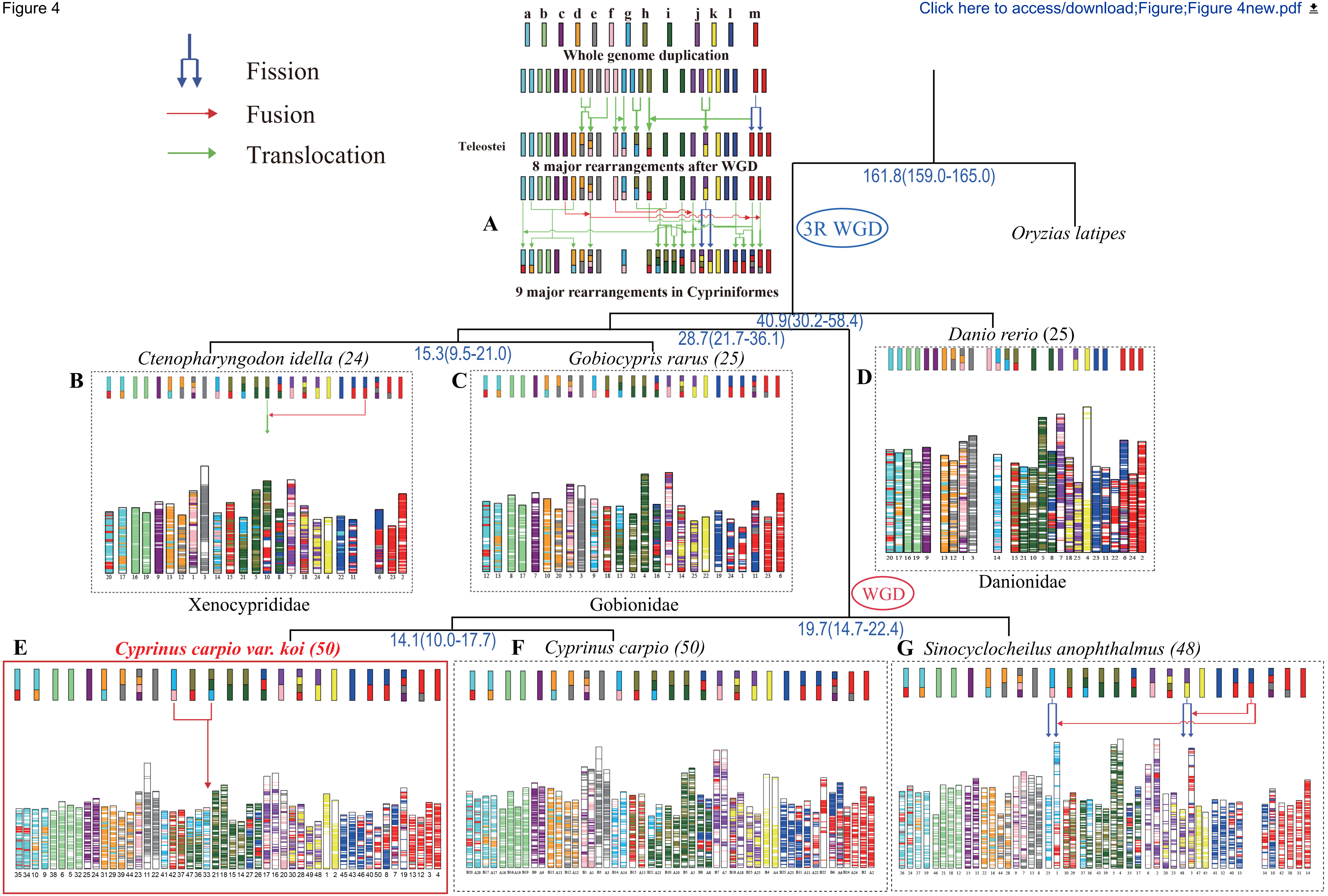

Supplementary materials

Click here to access/download
Supplementary Material
Supplementary materials 20250425new.docx

Respond letter

Click here to access/download
Supplementary Material
Review response 20250427.docx
