# Supplementary material for: Telomere-to-telomere chromosome-scale genome assemblies of black and golden koi carp variants support construction of an ancient karyotype of Cypriniformes
Source: Gigascience. 2025 Jul 26;14:giaf073. doi: 10.1093/gigascience/giaf073 (PMC13223729; doi:10.1093/gigascience/giaf073)
Supplement: giaf073_Authors_Response_To_Reviewer_Comments_original_submission [file giaf073_authors_response_to_reviewer_comments_original_submission.pdf]

Author's Response To Reviewer Comments

Dear editor and reviewers,
Thanks for your valuable comments. We have made careful revisions throughout our manuscript accordingly. Changes are highlighted in red in the revised manuscript. Point-by-point responses to your comments are provided as follows for your re-review.

Reviewer #1: In this paper, the authors conducted two telomere-to-telomere chromosome-level genome assemblies for black and golden koi carp variants of common carp (Cyprinus carpio) using PacBio HiFi, ONT, and Hi-C sequencing technologies. BUSCO results validated 99%. A total of approximately 55,000 genes were annotated. These data show similar results to previous reports. Based on phylogenetic analysis and divergence time estimation, they predicted that the two koi
carp split from each other about 8.1 million years ago. I think that the data are valuable for the researchers in this field and suitable for publication in this journal after revisions.
Response: We highly appreciate the reviewer's positive comments.

Several essential concerns should be addressed.
1. In the section introduction the authors mention as follows; "However, many important issues, such as how the ancestral chromosomes of teleost fishes evolved into those of Cypriniformes, and what changes occurred after the whole-genome duplication of ancestral chromosomes of Cypriniformes, remain largely unknown at present."
The changes after the whole-genome duplication of ancestral chromosomes of Cypriniformes were relatively well studied. The authors completely ignore previous genome reports for common carp. They should cite at least three references and discuss their differences and similarities.
Peng Xu et al., Nature Genetics volume 46, pages1212-1219 (2014)
Peng Xu et al., Nature Communications volume 10, Article number: 4625 (2019)
Jiong-Tang Li et al., Nature Genetics volume 53, pages1493-1503 (2021)
Response: Thanks for your nice advice. We removed these sentences "However, many issues, ...", and rewrote the entire Introduction according to your suggestions. We also added all references you suggested, also more refs we found at the same time, we think it can cover the historical common carp genomes now. We also added some discussion about these researches in lines 42-52.
These references are cited in our revised manuscript, see following:
1. Xu P, Zhang X, Wang X, Li J, Liu G, Kuang Y, et al. Genome sequence and genetic diversity of the common carp, Cyprinus carpio. Nature Genetics. 2014;46 11:1212-9. doi:10.1038/ng.3098.
2. Ma W, Zhu ZH, Bi XY, et al. Allopolyploidization is Not So Simple: Evidence from the Origin of the Tribe Cyprinini (Teleostei: Cypriniformes). Current molecular medicine. 2014;14
doi:10.2174/1566524014666141203101543.
3. Xu P, Xu J, Liu GJ, et al. The allotetraploid origin and asymmetrical genome evolution of the common carp. Nature Communications. 2019;10 doi:ARTN 462510.1038/s41467-019-12644-1.
4. Chen L, Li CY, Li BJ, et al. Evolutionary divergence of subgenomes in common carp provides insights into speciation and allopolyploid success. Fund Res-China. 2024;4 3:589-602. doi:10.1016/j.fmre.2023.06.011.
5. Kon T, Omori Y, Fukuta K, et al. The Genetic Basis of Morphological Diversity in Domesticated Goldfish. Current Biology. 2020;30 12:2260-+. doi:10.1016/j.cub.2020.04.034.
6. Chen ZL, Omori Y, Koren S, et al. De novo assembly of the goldfish genome and the evolution of genes after whole-genome duplication. Science Advances. 2019;5 6 doi:ARTN eaav054710.1126/sciadv.aav0547.
7. Li JT, Wang Q, Yang MD, et al. Parallel subgenome structure and divergent expression evolution of allo-tetraploid common carp and goldfish. Nature Genetics. 2021;53 10:1493-+. doi:10.1038/s41588-021-00933-9.
8. Ren L, Gao X, Cui JL, et al. Symmetric subgenomes and balanced homoeolog expression stabilize the establishment of allopolyploidy in cyprinid fish. Bmc Biol. 2022;20 1 doi:ARTN 20010.1186/s12915-022-01401-4.
9. Luo J, Chai J, Wen YL, et al. From asymmetrical to balanced genomic diversification during rediploidization: Subgenomic evolution in allotetraploid fish. Science Advances. 2020;6 22 doi:ARTN eaaz767710.1126/sciadv.aaz7677.

2. The authors should refer to the following at least three papers that investigated allotetraploid in goldfish, a species very closely related to common carp, and discuss them.
Zelin Chen et al., Science Advances 2019 Vol 5, Issue 6 DOI: 10.1126/sciadv.aav0547
Tetsuo Kon et al., Current Biology 2020 doi.org/10.1016/j.cub.2020.04.034
Jing Luo et al., Science Advances 2020 Vol 6, Issue 22 DOI: 10.1126/sciadv.aaz7677
Response: Thanks for your constructive comment. Yes, we have added these references in the revised manuscript. We also applied the divergence time of 11-13.75 Mya for Cyprinus carpio and Sinocyclocheilus species from the Jing Luo's study (Science Advances, 2020).
Reference in manuscript:
9. Luo J, Chai J, Wen YL, et al. From asymmetrical to balanced genomic diversification during rediploidization: Subgenomic evolution in allotetraploid fish. Science Advances. 2020;6 22 doi:ARTN eaaz767710.1126/sciadv.aaz7677.

3. The authors simply order the chromosome numbers by size in Supplementary Table S5. This should be adjusted to fit the three previous references (shown in comment 1). The authors should create a new supplementary table comparing the previously reported chromosomes with the chromosomes summarized in this paper.
Response: Many thanks for your good instructions. We added corresponding comparisons among black & golden koi and other two common carp references (Supplementary Table 6). We then obtained the one-to-one best-hit chromosomes between black & golden koi and C. carpio from Jiong-Tang Li's study (Nature Genetics, 2021), and reordered the chromosome numbers to be A and B subgenomes according to the references (Supplementary Tables 5).
Please see related changes in Supplementary Tables 5 and 6.

4. I reviewed the FASTA and GFF files. I recommend that you include gene symbols in the GFF file, otherwise it will be difficult to test the integrity of this reference sequence.
Response: Many thanks for your nice advice. Yes, we added gene symbols in the GFF files for both koi carps, and re-uploaded these data into the GigaScience FTP. The new gff files are named black.final.symbol.gff.gz and yellow.final.symbols.gff.gz.
For example:
scaffold_1 GeneWise mRNA 972449 1048204 62.65 + . ID=2862at7898-D42;Shift=0;
gene_symbol=ZN208;

5. The authors mention that "Based on a phylogenetic analysis and divergence time estimation, we predicted that the two koi carps split from each other about 8.1 million years ago." This is unlikely. Based on the results of six other papers (comments 1 and 2), these two variants appear to be much closer. Please review these six papers and recalculate your results.
Response: Many thanks for your constructive comments. You are right. The divergence time of 8.1 million years between two koi carps could be over-estimated. We reconstructed a divergence tree by using the following calibrated records: 180.0–251.5 million years ago (Mya) for D. rerio and O. latipes from Timetree website (https://timetree.org/home), 11.1-22.8 Mya for Cyprinus carpio and Sinocyclocheilus species [1], and 11-13.75 Mya for Cyprinus carpio and Sinocyclocheilus species [2]. The new divergence time between the two koi variants are about 5.3 Mya. See related change in lines 238-240.
Reference:
1. Li R, Wang X, Bian C, Gao Z, Zhang Y, Jiang W, et al. Whole-Genome Sequencing of Sinocyclocheilus maitianheensis Reveals Phylogenetic Evolution and Immunological Variances in Various Sinocyclocheilus Fishes. Front Genet. 2021;12:736500. doi:10.3389/fgene.2021.736500.
2. Luo J, Chai J, Wen Y, Tao M, Lin G, Liu X, et al. From asymmetrical to balanced genomic diversification during rediploidization: Subgenomic evolution in allotetraploid fish. Sci Adv. 2020;6 22:eaaz7677. doi:10.1126/sciadv.aaz7677.

Reviewer #2: The research paper entitled 'Telomere-to-telomere chromosome-scale genome assemblies of black and golden koi carp variants support construction of an ancient karyotype of Cypriniformes' by Chao Bian and collaborators reports the high-quality construction of genome assemblies for two cyprinid species using the latest sequencing technologies.
However, my opinion is that the manuscript is not adequately written and at the end the usefulness of their

research to the scientific community is not explicit.
The length of the paper is acceptable but I am confident that if you had added few sentences more to make your reasoning clearer it would be justified. However, I consider that in this form the paper needs serious restructure and rewriting and some important topics need to be addressed before resubmission.
In brief, the writing of the paper is median, the use of English is not adequate and some sentences are really hard to follow and extract the meaning. The GigaScience journal is not addressed solely to geneticists, evolutionary biologists or ichthyologists but to a much broader research audience which will surely get confused. At the discussion section, which is totally absent, you need to help more the readers to comprehend the information you provide on WGS and genome evolution in cyprinids. For example, the fact that we may also encounter hexaploid cyprinids etc?
Most manuscript's parts need serious restructuring and you may have to follow the guidelines of the journal, i.e., introduction, M&M, Results and Discussion or a combined "Results & Discussion" section.
Response: We would like to thank you for taking the time to give us valuable comments. We have made careful revisions accordingly.
1) For the usefulness of our research to the scientific community, we added a few sentences to clarify this in Introduction lines 61-67.
2) For the paper structure, since we submitted our manuscript as a Data Note. GigaScience journal recommended a Data Note paper contains the following sections: Context, Methods, Data Validation and quality control, Re-use potential. More details are here:
https://academic.oup.com/gigascience/pages/data_note. We also discussed with the editor of GigaScience journal and confirmed that the current structure is ok for publication. If you have further suggestions, please let us know, we will try our best to make it meet the journal's requirements.
3) Sorry for our poorly writing, now the manuscript has been edited by the language service from GigaScience Press, and the journal also has another round copy editing before publication, hope you are happy with it now. All changes are highlighted in the revised manuscript for your convenient reviewing.
4) For this question: "At the discussion section, which is totally absent, you need to help more the readers to comprehend the information you provide on WGS and genome evolution in cyprinids. For example, the fact that we may also encounter hexaploid cyprinids etc?" We added more paragragh in Conclusion line 268-273:
"The species from Cyprininae contain diploids (2n=2X=50 or 48), tetraploids (2n=4X=100), hexaploids (2n=6X≈150) [54] and higher polyploids (2n: 417–470) [55]. The koi carp in Cyprininae superfamily, Cyprinidae family, Cyprinoidei suborder is tetraploid (2n=4X=100). The chromosome-level genome assemblies of Cyprininae fish of different ploidies can largely help to study how the chromosome evolution in Cyprininae fish with diverse ploidies."

Mainly for the introduction section, it needs to contain the reasoning for the research conducted, past information and knowledge on the topic.
Response: Thanks for your nice advice. For the Introduction, we added 4 carp genome studies, such as the common carp (Cyprinus carpio) and goldfish (Carassius auratus), in lines 42-60.

Do the black and golden variants interbreed? If they split 8 Mya, can they be considered separate species? Obviously not since they still keep the "Cyprinus carpio var. koi" as a Linnean species name. The quantity of the data is huge but the discussion on the results is very poor and superficial with references stopping at line 205!
Response: Many thanks for your questions and constructive comments. Black and golden variants can interbreed. We are sorry for pointing out that the predicted divergence time between both koi variants, 8 Mya, could be over-estimated. In fact, we reconstructed a divergence tree by using the following calibrated records: 180.0–251.5 million years ago (Mya) for D. rerio and O. latipes from Timetree website (https://timetree.org/home), 11.1-22.8 Mya for Cyprinus carpio and Sinocyclocheilus species [1], and 11-13.75 Mya for Cyprinus carpio and Sinocyclocheilus species [2]. The new divergence time between the two kois was narrowed to about 5.3 Mya (see Figure 3).
For discussion, we focused on elevating quality of the first two T2T genome assemblies of koi carps in this study, filling the gap in the current lack of genome resources in koi carps, and laying a good reference for large-scale resequencing to examine body colors and comparative genomics of koi in the coming future. As a Data Note paper, we majorly exhibited the high quality of both genome assemblies in the result section, the discussion part is really weaker than a Research article, but we have added some more sentences in Conclusion in lines 268-298, hope these are helpful now.

Reference:
1. Li R, Wang X, Bian C, Gao Z, Zhang Y, Jiang W, et al. Whole-Genome Sequencing of Sinocyclocheilus maitianheensis Reveals Phylogenetic Evolution and Immunological Variances in Various Sinocyclocheilus Fishes. Front Genet. 2021;12:736500. doi:10.3389/fgene.2021.736500.
2. Luo J, Chai J, Wen Y, Tao M, Lin G, Liu X, et al. From asymmetrical to balanced genomic diversification during rediploidization: Subgenomic evolution in allotetraploid fish. Sci Adv. 2020;6 22:eaaz7677. doi:10.1126/sciadv.aaz7677.

Minor comments:
Lines 17-19: it needs a better background explanation. I cannot understand its "precious variability in skin color" neither the "excellent model to study chromosome changes after undergoing WGD".
Response: Thanks for your nice advice. We removed "precious variability in skin color" and rewritten this section. See more details in lines 23-25.
"Background: Koi carp, a variant of the common carp, is one of the most popular ornamental fish. Its genomic resources can help us better understand chromosome evolution and color phenotypes in cyprinid fish."

Line 29: better rephrase "of their whole genomes"
Response: Yes, it is done (in line 32). We revised this sentence to be 'Repetitive sequences occupy an estimated 636 Mb (41%) of the genomes.'.

Line 36: rephrase "which support to predict an ancient…"
Response: Yes, this sentence is rephrased in lines 36-37 as 'Our study offers genome assemblies capable of predicting an ancient karyotype of Cypriniformes,......'.

Line 61: why "on the other hand"?
Response: It was removed in the revised manuscript.

Line 62: "allotetraploid species with two diploid progenitors that diverged about 23 million years ago (Mya) and later hybridized about 12.4 Mya". The [2] is not correct at all and you should find the right one.
Lines 67-80: This last paragraph at the introduction section needs very serious restructure without duplicating the information you provide also in the abstract and the next M&M sections!
Response: Many thanks for your good advice. We thoroughly re-wrote the Introduction section and removed the previously inaccurate conclusion. (see lines 42-67)

Line 31: delete "from each other", it's by default when they split.
Line 32: either "cypriniforms" or "cypriniform species"; see also lines 38, 262, 265, 268, 289, 295, 310, 352.
Line 35: "Current data high-quality data…"
Line 47: delete "as we know, the"
Line 48: better move "especially in ornamental species" after the "overall market value" to make sense.
Line 51: rephrase "this makes it"
Line 54: better mention that miR-430 is a microRNA which is not obvious to everybody.
Line 96: delete "and then"
Line 99: "libraries were prepared with an Oxford…"
Response: We apologize for these mistakes. They were corrected in our revised manuscript.

Line 87: better also cite next to the technologies employed and the type of data produced (short vs long reads) to help readers understand why you did all that.
Response: Many thanks for your good advice. We cited a Review paper in line 78, entitled 'Advances in Whole Genome Sequencing: Methods, Tools, and Applications in Population Genomics'. This review comprehensively reported the development and iteration of genome sequencing technologies, and provided detailed comparison of three sequencing technologies of Sanger, short reads and long reads [4].
Reference: 4. Lu Y, Li M, Gao Z, Ma H, Chong Y, Hong J, et al. Advances in Whole Genome Sequencing: Methods, Tools, and Applications in Population Genomics. Int J Mol Sci. 2025;26 1 doi:10.3390/ijms26010372.

Line 104: "Hi-C Kit and DpnII enzyme" with the enzyme name written in italics.
Response: Yes, it is done (in line 91).

Line 124: delete "by' before "using" and correct it all along the document too, e.g., in lines 145, 188, 207
Response: Yes, these are all corrected.

Line 166: "...database and were aligned to our assembled…"
Line 183: change to lower case "idella"; see also line 277
Line 191: for PhyML, it's not the reference [38]
Response: These are all revised. See more details in lines 151, 149, 167, 260 and 175.

Line 194: for TimeTree, better used the respective reference (Kumar et al., 2017). It is also strange but when I also used the website and entered the two species to estimate the divergence time, it showed to me 224 Mya (180-251.5 Mya); can you please explain how you got the 140-170 Mya estimate?
Response: Sorry for the mistake. In fact, we reconstructed a divergence tree by using the following calibrated records: 180.0–251.5 million years ago (Mya) for D. rerio and O. latipes from Timetree website (https://timetree.org/home), 11.1-22.8 Mya for Cyprinus carpio and Sinocyclocheilus species [1], and 11-13.75 Mya for Cyprinus carpio and Sinocyclocheilus species [2]. The new divergence time between two kois are narrowed to about 5.3 Mya (see Figure 3).
Reference:
1. Li R, Wang X, Bian C, Gao Z, Zhang Y, Jiang W, et al. Whole-Genome Sequencing of Sinocyclocheilus maitianheensis Reveals Phylogenetic Evolution and Immunological Variances in Various Sinocyclocheilus Fishes. Front Genet. 2021;12:736500. doi:10.3389/fgene.2021.736500.
2. Luo J, Chai J, Wen Y, Tao M, Lin G, Liu X, et al. From asymmetrical to balanced genomic diversification during rediploidization: Subgenomic evolution in allotetraploid fish. Sci Adv. 2020;6 22:eaaz7677. doi:10.1126/sciadv.aaz7677.

Line 205: "teleosts" in plural; see also line 259
Line 208: better delete "those"
Line 225: I read for both species, N50 is 30.0 Mb in Table 1!
Line 226: delete "Meanwhile"; "we also obtained…"
Response: These are all revised. See more details in lines 186, 188, 191, 242, 321 and 208.

Lines 231-234: you better mention that you followed a synteny criterion for the chromosome numbering in both species, i.e., chromosome 1 in black is closer to chromosome 1 in the golden koi.
Response: Thanks for your nice advice. We added corresponding comparisons among black & golden koi and other two common carp references (Supplementary Table 6). We then obtained the one-to-one best-hit chromosomes between black & golden koi and C. carpio from Jiong-Tang Li's study (Nature Genetics, 2021), and reordered the chromosome numbers to be A and B subgenomes according to the references (Supplementary Tables 5).
Please see related changes in Supplementary Tables 5 and 6.

Lines 239-240: what does the Mercury quality value of 46.8 stand for?
Response: Sorry for not clarifying this clearly. The Merqury can estimate the base-level accuracy by comparing k-mers in the assemblies to those from the HiFi reads. Therefore, the Merqury quality value of 46.8 stands for a per-base accuracy rate for assembly to be 0.9999988. We have provided more information in line 222.
"The Merqury program indicated that the quality values of both assemblies were 46.8, equating to a per-base accuracy rate of 0.9999988."

Lines 244-245: in practice, for the amount of repetitive sequences you may better write "636.21 and 636.24 Mb" (see Table S6)
Response: Yes, it is done, thanks. See the corrections in line 227.
"We predicted that the black and golden koi carp genomes contain 636.21 and 636.24 Mb of repetitive sequences."

Line 270: Figure 4 in the manuscript text (lines 318-326) seems to be not the same with the one uploaded at the end of the pdf file: see for example Cyprinus carpio wuyuanensis et letters A to G missing!
Response: Sorry for our mistake. We submitted a corrected PDF file of Figure 4 in our revised manuscript.

Line 280: rephrase "one paired ancestral chromosomes were fused into two other chromosomes from different origins"
Response: Yes, it is done. We revised this sentence as 'Two fusion events and two fission events were occurred in chromosomes of Sinocyclocheilus species' in lines 262-263.

Lines 284-295: the Conclusion part needs a serious restructure without duplicating the information provided so far in the abstract, the introduction, and the results sections.
Response: Many thanks for your nice advice. Yes, we re-wrote the Conclusion section in lines 268-304 of our revised manuscript.

Line 303: "both genome sequencings".
Supplementary Table 10: there is no such reference in the manuscript; in the last column, better use "Haploid Chromosome Number" as a header.
Response: Thanks for your detailed instructions. Yes, we corrected these issues in our revised manuscript. See more details in line 312 and the new Table S11.

Editorial Manager®

Cookie Preference Center

We use cookies which are necessary to make our site work. We may also use additional cookies to analyze, improve and personalize our content and your digital experience. For more information, see our Cookie Policy.

You may choose not to allow some types of cookies. However, blocking some types may impact your experience of our site and the services we are able to offer. See the different category headings below to find out more or change your settings.

Allow all

Manage Consent Preferences

Strictly Necessary Cookies

Always active

These cookies are necessary for the website to function and cannot be switched off in our systems. They are usually only set in response to actions made by you which

amount to a request for services, such as setting your privacy preferences, logging in or filling in forms. You can set your browser to block or alert you about these cookies, but some parts of the site will not then work.

Functional Cookies

These cookies enable the website to provide enhanced functionality and personalisation. They may be set by us or by third party providers whose services we have added to our pages. If you do not allow these cookies then some or all of these services may not function properly.

Performance Cookies

These cookies allow us to count visits and traffic sources so we can measure and improve the performance of our site. They help us to know which pages are the most and least popular and see how visitors move around the site.

Targeting Cookies

These cookies may be set through our site by our advertising partners. They may be used by those companies to build a profile of your interests and show you relevant adverts on other sites. If you do not allow these cookies, you will experience less targeted advertising.

Cookie List

Clear

Apply Cancel

Consent Leg.Interest

Confirm my choices

Powered by onetrus
