# Supplementary material for: Telomere-to-telomere chromosome-scale genome assemblies of black and golden koi carp variants support construction of an ancient karyotype of Cypriniformes
Source: Gigascience. 2025 Jul 26;14:giaf073. doi: 10.1093/gigascience/giaf073 (PMC13223729; doi:10.1093/gigascience/giaf073)
Supplement: giaf073_Reviewer_1_Report_Original_Submission — Yoshihiro Omori -- 1/23/2025 [file giaf073_reviewer_1_report_original_submission.pdf]

Reviewer Report

Title: Telomere-to-telomere chromosome-scale genome assemblies of black and golden koi carp variants support construction of an ancient karyotype of Cypriniformes

Version: Original Submission **Date:** 1/23/2025

Reviewer name: Yoshihiro Omori

Reviewer Comments to Author:

In this paper, the authors conducted two telomere-to-telomere chromosome-level genome assemblies for black and golden koi carp variants of common carp (Cyprinus carpio) using PacBio HiFi, ONT, and Hi-C sequencing technologies. BUSCO results validated 99%. A total of approximately 55,000 genes were annotated. These data show similar results to previous reports. Based on phylogenetic analysis and divergence time estimation, they predicted that the two koicarp split from each other about 8.1 million years ago. I think that the data are valuable for the researchers in this field and suitable for publication in this journal after revisions. Several essential concerns should be addressed.1. In the section introduction the authors mention as follows; "However, many important issues, such as how the ancestral chromosomes of teleost fishes evolved into those of Cypriniformes, and what changes occurred after the whole-genome duplication of ancestral chromosomes of Cypriniformes, remain largely unknown at present."The changes after the whole-genome duplication of ancestral chromosomes of Cypriniformes were relatively well studied. The authors completely ignore previous genome reports for common carp. They should cite at least three references and discuss their differences and similarities.Peng Xu et al., Nature Genetics volume 46, pages1212-1219 (2014)Peng Xu et al., Nature Communications volume 10, Article number: 4625 (2019)Jiong-Tang Li et al., Nature Genetics volume 53, pages1493-1503 (2021)2. The authors should refer to the following at least three papers that investigated allotetraploid in goldfish, a species very closely related to common carp, and discuss them.Zelin Chen et al., Science Advances 2019 Vol 5, Issue 6 DOI: 10.1126/sciadv.aav0547Tetsuo Kon et al., Current Biology 2020 doi.org/10.1016/j.cub.2020.04.034Jing Luo et al., Science Advances 2020 Vol 6, Issue 22 DOI: 10.1126/sciadv.aaz76773. The authors simply order the chromosome numbers by size in Supplementary Table S5. This should be adjusted to fit the three previous references (shown in comment 1). The authors should create a new supplementary table comparing the previously reported chromosomes with the chromosomes summarized in this paper.4. I reviewed the FASTA and GFF files. I recommend that you include gene symbols in the GFF file, otherwise it will be difficult to test the integrity of this reference sequence.5. The authors mention that "Based on a phylogenetic analysis and divergence time estimation, we predicted that the two koi carps split from each other about 8.1 million years ago." This is unlikely. Based on the results of six other papers (comments 1 and 2), these two variants appear to be much closer. Please review these six papers and recalculate your results.

Level of Interest

Please indicate how interesting you found the manuscript: Choose an item.

Quality of Written English

Please indicate the quality of language in the manuscript: Choose an item.

Declaration of Competing Interests

Please complete a declaration of competing interests, considering the following questions:

- Have you in the past five years received reimbursements, fees, funding, or salary from an organisation that may in any way gain or lose financially from the publication of this manuscript, either now or in the future?
- Do you hold any stocks or shares in an organisation that may in any way gain or lose financially from the publication of this manuscript, either now or in the future?
- Do you hold or are you currently applying for any patents relating to the content of the manuscript?
- Have you received reimbursements, fees, funding, or salary from an organization that holds or has applied for patents relating to the content of the manuscript?
- Do you have any other financial competing interests?
- Do you have any non-financial competing interests in relation to this paper?

If you can answer no to all of the above, write 'I declare that I have no competing interests' below. If your reply is yes to any, please give details below.

I declare that I have no competing interests.

I agree to the open peer review policy of the journal. I understand that my name will be included on my report to the authors and, if the manuscript is accepted for publication, my named report including any attachments I upload will be posted on the website along with the authors' responses. I agree for my report to be made available under an Open Access Creative Commons CC-BY license (http://creativecommons.org/licenses/by/4.0/). I understand that any comments which I do not wish to be included in my named report can be included as confidential comments to the editors, which will not be published.

Choose an item.

To further support our reviewers, we have joined with Publons, where you can gain additional credit to further highlight your hard work (see: https://publons.com/journal/530/gigascience). On publication of this paper, your review will be automatically added to Publons, you can then choose whether or not to claim your Publons credit. I understand this statement.

Yes Choose an item.
