# Supplementary material for: Telomere-to-telomere chromosome-scale genome assemblies of black and golden koi carp variants support construction of an ancient karyotype of Cypriniformes
Source: Gigascience. 2025 Jul 26;14:giaf073. doi: 10.1093/gigascience/giaf073 (PMC13223729; doi:10.1093/gigascience/giaf073)
Supplement: giaf073_Reviewer_2_Report_Original_Submission — Costas Tsigenopoulos -- 2/6/2025 [file giaf073_reviewer_2_report_original_submission.pdf]

Reviewer Report

Title: Telomere-to-telomere chromosome-scale genome assemblies of black and golden koi carp variants support construction of an ancient karyotype of Cypriniformes

Version: Original Submission **Date:** 2/6/2025

Reviewer name: Costas Tsigenopoulos

Reviewer Comments to Author:

The research paper entitled 'Telomere-to-telomere chromosome-scale genome assemblies of black and golden koi carp variants support construction of an ancient karyotype of Cypriniformes' by Chao Bian and collaborators reports thehigh-quality construction of genome assemblies for two cyprinid species using the latest sequencing technologies.However, my opinion is that the manuscript is not adequately written and at the end the usefulness of their research to the scientific community is not explicit.The length of the paper is acceptable but I am confident that if you had added few sentences more to make your reasoning clearer it would be justified. However, I consider that in this form the paper needs serious restructure and rewriting and some important topics need to be addressed before resubmission.In brief, the writing of the paper is median, the use of English is not adequate and some sentences are really hard to follow and extract the meaning. The GigaScience journal is not addressed solely to geneticists, evolutionary biologists or ichthyologists but to a much broader research audience which will surely get confused. At the discussion section, which is totally absent, you need to help more the readers to comprehend the information you provide on WGS and genome evolution in cyprinids. For example, the fact that we may also encounter hexaploid cyprinids etc?Most manuscript's parts need serious restructuring and you may have to follow the guidelines of the journal, i.e., introduction, M&M, Results and Discussion or a combined "Results & Discussion" section. Mainly for the introduction section, it needs to contain the reasoning for the research conducted, past information and knowledge on the topic. Do the black and golden variants interbreed? If they split 8 Mya, cab they be considered separate species? Obviously not since they still keep the "Cyprinus carpio var. koi" as a Linnean species name. The quantity of the data is huge but the discussion on the results is very poor and superficial with references stopping at line 205!Minor comments:Lines 17-19: it needs a better background explanation. I cannot understand its "precious variability in skin color" neither the "excellent model to study chromosome changes after undergoing WGD".Line 29: better rephrase "of their whole genomes"Line 31: delete "from each other", it's by default when they split.Line 32: either "cypriniforms" or "cypriniform species"; see also lines 38, 262, 265, 268, 289, 295, 310, 352.Line 35: "Current data high-quality data…"Line 36: rephrase "which support to predict an ancient…"Line 47: delete "as we know, the"Line 48: better move "especially in ornamental species" after the "overall market value" to make sense.Line 51: rephrase "this makes it"Line 54: better mention that miR-430 is a microRNA which is not obvious to everybody.Line 61: why "on the other hand"?Line 62: "allotetraploid species with two diploid progenitors that diverged about 23 million years ago (Mya) and later hybridized about 12.4 Mya". The [2] is not correct at all and you should find the right one.Lines 67-80: This last paragraph at the introduction section needs very serious restructure without duplicating the information you provide also

in the abstract and the next M&M sections!Line 87: better also cite next to the technologies employed and the type of data produced (short vs long reads) to help readers understand why you did all that.Line 96: delete "and then"Line 99: "libraries were prepared with an Oxford…"Line 104: "Hi-C Kit and DpnII enzyme" with the enzyme name written in italics.Line 124: delete "by' before "using" and correct it all along the document too, e.g., in lines 145, 188, 207Line 166: "...database and were aligned to our assembled…"Line 183: change to lower case "idella"; see also line 277Line 191: for PhyML, it's not the reference [38]Line 194: for TimeTree, better used the respective reference (Kumar et al., 2017). It is also strange but when I also used the website and entered the two species to estimate the divergence time, it showed to me 224 Mya (180-251.5 Mya); can you please explain how you got the 140-170 Mya estimate?Line 205: "teleosts" in plural; see also line 259Line 208: better delete "those"Line 225: I read for both species, N50 is 30.0 Mb in Table 1!Line 226: delete "Meanwhile"; "we also obtained…"Lines 231-234: you better mention that you followed a synteny criterion for the chromosome numbering in both species, i.e., chromosome 1 in black is closer to chromosome 1 in the golden koi.Lines 239-240: what does the Mercury quality value of 46.8 stand for?Lines 244-245: in practice, for the amount of repetitive sequences you may better write "636.21 and 636.24 Mb" (see Table S6)Line 270: Figure 4 in the manuscript text (lines 318-326) seems to be not the same with the one uploaded at the end of the pdf file: see for example Cyprinus carpio wuyuanensis et letters A to G missing!Line 280: rephrase "one paired ancestral chromosomes were fused into two other chromosomes from different origins"Lines 284-295: the Conclusion part needs a serious restructure without duplicating the information provided so far in the abstract, the introduction, and the results sections.Line 303: "both genome sequencings".Supplementary Table 10: there is no such reference in the manuscript; in the last column, better use "Haploid Chromosome Number" as a header.

Level of Interest

Please indicate how interesting you found the manuscript: Choose an item.

Quality of Written English

Please indicate the quality of language in the manuscript: Choose an item.

Declaration of Competing Interests

Please complete a declaration of competing interests, considering the following questions:

- Have you in the past five years received reimbursements, fees, funding, or salary from an organisation that may in any way gain or lose financially from the publication of this manuscript, either now or in the future?
- Do you hold any stocks or shares in an organisation that may in any way gain or lose financially from the publication of this manuscript, either now or in the future?
- Do you hold or are you currently applying for any patents relating to the content of the manuscript?

- Have you received reimbursements, fees, funding, or salary from an organization that holds or has applied for patents relating to the content of the manuscript?
- Do you have any other financial competing interests?
- Do you have any non-financial competing interests in relation to this paper?

If you can answer no to all of the above, write 'I declare that I have no competing interests' below. If your reply is yes to any, please give details below.

I declare that I have no competing interests

I agree to the open peer review policy of the journal. I understand that my name will be included on my report to the authors and, if the manuscript is accepted for publication, my named report including any attachments I upload will be posted on the website along with the authors' responses. I agree for my report to be made available under an Open Access Creative Commons CC-BY license (http://creativecommons.org/licenses/by/4.0/). I understand that any comments which I do not wish to be included in my named report can be included as confidential comments to the editors, which will not be published.

Choose an item.

To further support our reviewers, we have joined with Publons, where you can gain additional credit to further highlight your hard work (see: https://publons.com/journal/530/gigascience). On publication of this paper, your review will be automatically added to Publons, you can then choose whether or not to claim your Publons credit. I understand this statement.

Yes Choose an item.
